# Dynamic roughening of cities driven by multiplicative noise

**DOI:** 10.1038/s41467-026-74456-4

**Published:** 2026-06-25

**Authors:** Martin Hendrick, Gabriele Manoli

**Affiliations:** Laboratory of Urban and Environmental Systems, École Polytechnique Fédérale de Lausanne, Lausanne, Switzerland

**Keywords:** Statistical physics, thermodynamics and nonlinear dynamics, Environmental sciences

## Abstract

The evolution of urban landscapes is rapidly altering the surface of our planet. Yet our understanding of the urbanization phenomenon remains far from complete. A fundamental challenge is to describe spatiotemporal changes in the built environment, both vertical and horizontal. In this work, we model global building-height dynamics as a zero-dimensional geometric Brownian motion (GBM): multiplicative noise produces stochastic fluctuations around a drift linked to economic growth. To account for intra-city correlations, we extend the GBM with spatial coupling, revealing how local interactions effectively mitigate noise-driven fluctuations and shape urban morphology. In the continuum limit, the spatial model reduces to the Kardar-Parisi-Zhang (KPZ) equation, and roughness exponents estimated from data fall within the KPZ range for most cities. Our results indicate that multiplicative noise, moderated by local coupling, governs the evolution of urban roughness, placing city growth within a well-established statistical-physics framework.

## Abstract (web summary)

This study shows that cities grow upward through random, self-amplifying processes moderated by local interactions, linking urban form and economic growth to universal patterns from statistical physics.

## Introduction

Cities worldwide are experiencing rapid and heterogeneous growth, driven by economic expansion, demographic change, and technological development. High-resolution global datasets show that between 1985 and 2015, urban land cover increased by nearly 10,000 km^2^ per year, with the fastest expansion occurring in China, India, and Africa^1^. This large-scale urbanization of the Earth surface puts increasing pressure on natural ecosystems and creates sustainability challenges both locally and globally^2^. Hence, despite progress in our understanding of urban development at the macroscopic level^3,4^, there is an urgent need for quantitative models that capture the detailed spatiotemporal dynamics of urban structure in order to anticipate change, guide urban planning, and inform decision-makers with robust scientific information^5^.

Urban expansion is driven by a small set of recurring forces whose relative importance varies across regions and time. On the demand side, cities expand as urban populations grow (via natural increase, internal migration, and the reclassification/absorption of settlements into urban areas) and as economic development raises per-capita demand for housing and space^6^. Specifically, a global meta-analysis found that urban land expansion is systematically related to both urban population growth and GDP-per-capita growth, with marked regional differences (e.g., stronger income effects in some contexts and stronger demographic effects in others)^7^. In the standard urban-economics view, outward spatial growth is the joint outcome of a growing population, rising incomes (which increase land consumption), and falling commuting costs^8^. Transportation technologies, especially highway and railway infrastructure, reduce generalized travel costs and thereby facilitate suburbanization and low-density outward growth^9,10^. Finally, land-use regulation, fragmented governance, fiscal incentives, and the availability of developable fringe land can promote scattered “leapfrog” development, while geography and infrastructure shape where and how expansion occurs^11^. Beyond horizontal land take, many cities also accommodate growth through vertical expansion (densification via taller buildings). In standard urban economics, rising demand for central locations bids up land values and developers respond by substituting capital for land (adding floor space by building higher) until the marginal revenue from additional space is balanced by the increasing marginal costs of height (structure, foundations, fire safety, and intra-building transport)^12^.

Despite this variety of social, economic, political, and technological forces that shape the evolution of urban areas and the wide spectrum of geographical and historical constraints, empirical research has uncovered striking regularities in the structure and dynamics of global cities, such as Zipf’s law for city size distributions^13,14^ and urban scaling laws^3^ for various city properties - including mean building height^15^. Classical urban economics models, including the Alonso–Muth–Mills model^16^ and central place theory^17^, provide equilibrium descriptions of urban spatial structure, while economic geography frameworks incorporate increasing returns and trade costs^18^. However, these approaches often treat cities as point-like objects or assume static monocentric structures, limiting their ability to describe the actual spatiotemporal evolution of cities.

In parallel, statistical physics and complexity science have offered dynamic and spatially explicit models. Fractal analyses and diffusion-limited aggregation models reproduce features of urban morphology^19–21^, while correlated percolation models describe urban sprawl and merging^22^. Reaction-diffusion frameworks have also been used to describe the horizontal expansion of urban areas^23,24^, including the co-evolution of population and transport networks over multi-decadal timescales^10^. Recent work has linked urban growth fronts to kinetic roughening: while some cities fall into Kardar-Parisi-Zhang (KPZ) or Edwards-Wilkinson universality classes^25^, others seem to be inconsistent with KPZ and are better captured by alternative dynamical scaling ansatz^26^. Stochastic models of city size evolution^4,27^, capture deviations from Zipf’s law due to random shocks and finite-time effects, and cross-diffusion models have addressed interactions of density and housing prices at the intra-city level^28^.

Many of these urban growth models are based on multiplicative rather than additive noise - in contrast to standard surface growth models. Multiplicative noise has a long history in urban growth and landscape evolution — from Gibrat’s law^29^ to random-growth explanations of Zipf’s law for city sizes, economies^4,27,30,31^ and geomorphology^32^. More generally, multiplicative noise encapsulates a wide variety of phase-transition phenomena and generates a rich zoo of universality classes^33^. In socio-economic contexts, the same mechanism governs how resources concentrate or disperse^34^.

Although some studies in the economics literature addresses cities’ vertical expansion^35^, most of the aforementioned physics-based research on urban growth focuses on horizontal sprawl only. Yet, recent observations indicate a global shift towards building upward^36^. Here we address this knowledge gap by introducing a physics-based framework — based on a minimalist stochastic model with multiplicative noise — that jointly reproduces vertical and horizontal growth in world cities and quantifies intra-urban volumetric dynamics. We start from a zero-dimensional Geometric Brownian Motion (GBM), which encapsulates local stochastic proportional growth in an analytically tractable way. We then introduce spatial coupling by adding an interaction term between neighboring sites, creating a spatially extended system. Using a mean-field approximation, we study the emergence of distinct growth regimes characterized by the mean and variance of building heights. Finally, by taking the continuum limit, we arrive at a stochastic partial differential equation connected to the KPZ universality class^37^ via the Hopf–Cole transformation. The deterministic growth rate of our model is found to be directly linked to the local Gross Domestic Product (GDP), connecting economic productivity to the dynamics of urban structure. Our theoretical framework thus directly couples economic productivity to the spatial growth of cities, demonstrating its connection to the KPZ equation and the theory of growing interfaces in the physical and biological domains, from particle deposition to bacterial growth^38^.

## Results

As a first step, we analysed urban vertical growth through the lens of geometric Brownian motion (Eq. (2); see Methods), a canonical model for multiplicative processes in which increments are proportional to the current state. Its logarithmic growth rate, defined for each city as *γ* = *μ* − *σ*^2^/2, collapses the two GBM parameters (deterministic drift *μ* and noise amplitude *σ*) into a single measure that is readily comparable across cities, countries, and time (Eq. (4); see Methods).

**Fig. 1:**
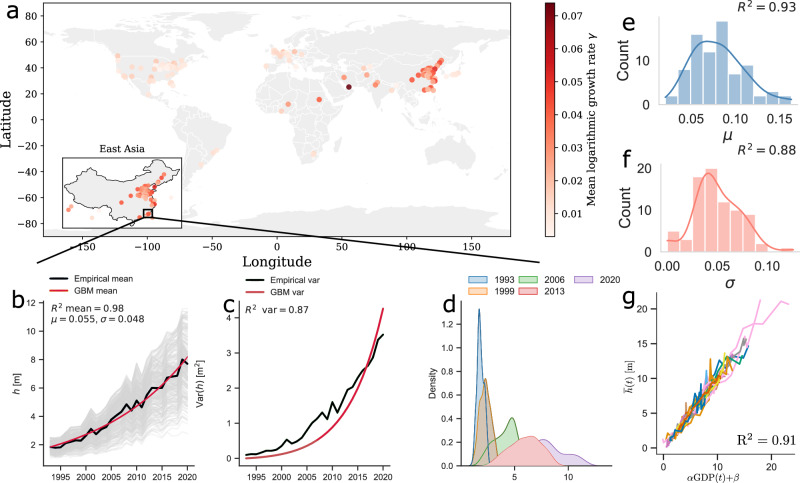
Geometric Brownian Motion (GBM) growth of world cities. **a** World map of analysed cities colored by the mean logarithmic building-height growth rate *γ*; inset highlights East Asia. Guangzhou, China, is shown as an illustrative case (highlighted by the black box in the inset). **b**–**d** GBM-data comparison for Guangzhou: **b** per-pixel building heights (gray) with empirical and GBM mean height overlaid (Eq. (7)); **c** variance of heights, empirical vs GBM (Eq. (10)); **d** kernel-density estimates of the height distribution for five benchmark years. **e**, **f** GBM parameter estimates for all East Asian cities analyzed where *R*^2^ refers to empirical mean (**f**) and variance (**g**) compared to GBM. **g** Temporal lienar relation between city mean height $\overline{h}$ and city Gross Domestic Product (GDP) (*α*, *β* constant determined for each city; one line represent one city). Basemap in panel **a**: Natural Earth Admin 0--Countries, 1:110m cultural vectors.

Applying the formalism to building height (*h*) observations from a sample of 500 metropolitan areas over the period 1993–2020, we find that high logarithmic growth rates cluster in China and South-East Asia, mirroring the region’s rapid economic ascent (Fig. 1a). In contrast, for example, U.S. and European cities exhibit markedly lower *γ* values. In addition, we find that the logarithmic growth rate is independent of city size, implying a size-independent growth law consistent with Gibrat-type regularity (Supplementary Fig. 1).

Focusing on individual cities helps clarify the underlying mechanisms. Guangzhou exemplifies a fast-growing Chinese center: its empirical building-height trajectory closely matches GBM predictions, in which the height at each location follows a GBM path with constant parameters for the city. This holds for both the mean $\overline{h}(t)$ and the variance Var(*h*) (*R*^2^ ≈ 0.9; Fig. 1b and c). We extended this analysis to the 116 Chinese cities and surrounding areas (Fig. 1e and f), yielding a pair of parameters (*μ*, *σ*) for each city and consistently good *R*^2^ values for both the mean and variance fits. Remarkably, we observed a linear relationship over time between mean building height and city GDP, indicating that the intrinsic city growth rate *μ* is directly tied to GDP growth (Fig. 1g).

By contrast, New York City (Supplementary Fig. 3; or Phoenix for a less extreme example provided in Supplementary Fig. 4), exemplifies a mature USA city that deviates from GBM predictions. In particular, the variance deviates because substantial height dispersion was already present in 1993 and subsequent growth approaches a saturation plateau where fluctuations dominate (Supplementary Fig. 6; a behavior that can be reproduced by extending the GBM model to the stochastic Fisher-Kolmogorov equation, see Eq. (18) and Methods). Nevertheless, GDP continues to track the mean height reliably for most of the USA cities (Supplementary Fig. 5), underscoring that economic output reflects average vertical expansion even in late-stage urban growth dynamics.

**Fig. 2:**
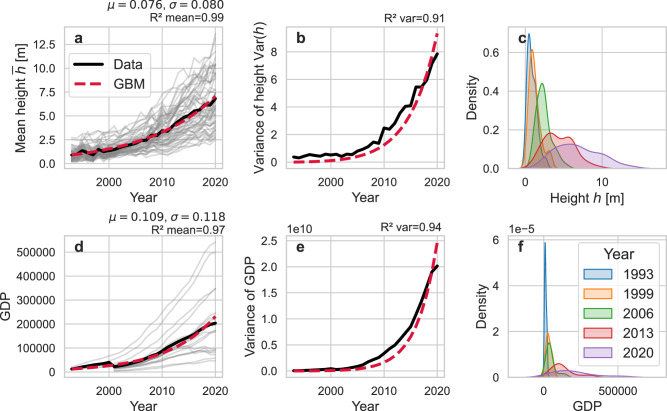
City-level GBM behavior for China. **a** Time series of the mean building height $\overline{h}(t)$ for each city (gray) and the national average (black), compared with the Geometric Brownian Motion (GBM) fit (red). **b** Corresponding height variance ${\rm{Var}}\left(h(t)\right)$ and its GBM prediction. **c** Kernel-density estimates of the height distribution for five benchmark years (1993-2020). **(d)** National-average Gross Domestic Product (GDP) (black) with GBM fit (red), GDP for each city (gray; city level GDP data are only available for the main Chinese cities^78^). **e** Variance of GDP and GBM prediction. **f** KDEs of the GDP distribution for the same benchmark years. Inset boxes list the fitted GBM parameters (*μ*, *σ*) and coefficients of determination *R*^2^ for mean and variance. The analysis extends the earlier intra-urban study to the inter-city scale.

This intra-urban toy model is also generalizable to the national scale. For China, a single drift-volatility pair reproduces the temporal evolution of the country-average building height (Fig. 2a–c). The same approach captures the temporal dynamics of city GDP (Fig. 2d–f), with drift and volatility comparable to those inferred for country-average and intra-urban building-height dynamics (Fig. 1, see also Supplementary Fig. 6 for the United States as a contrasting case). Taken together, these results support the self-similarity of urban growth across scales, as previously noted by^31^.

Despite GBM's effectiveness in reproducing city- and national-scale building-height dynamics observed in fast-growing cities (large *γ*), it does not account for spatial correlations within individual cities. To overcome this limitation, we extend the GBM model by adding local interactions between neighboring locations resulting in a spatio-temporal stochastic growth model (see also Methods):

1 $${\rm{d}}{h}_{i}=\mu {h}_{i}\,{\rm{d}}t+\sigma {h}_{i}\,{\rm{d}}{W}_{i}(t)+k\mathop{\sum }\limits_{{i}^{{\prime} }\in {\mathcal{N}}(i)}({h}_{i{\prime} }-{h}_{i})\,{\rm{d}}t,$$

where *h*_*i*_(*t*) is the building height at site *i*, *t* is time, *W*_*i*_ is the standard Brownian motion, *k* is the coupling strength which controls the intensity of local spatial interaction, and ${\mathcal{N}}(i)$ denotes the neighbors of site *i*. This approach is a spatially embedded and local version of various models previously studied in the literature^4,34,39^, and it constitutes a stochastic and parsimonious variant of the reaction-diffusion model ^10^.

**Fig. 3:**
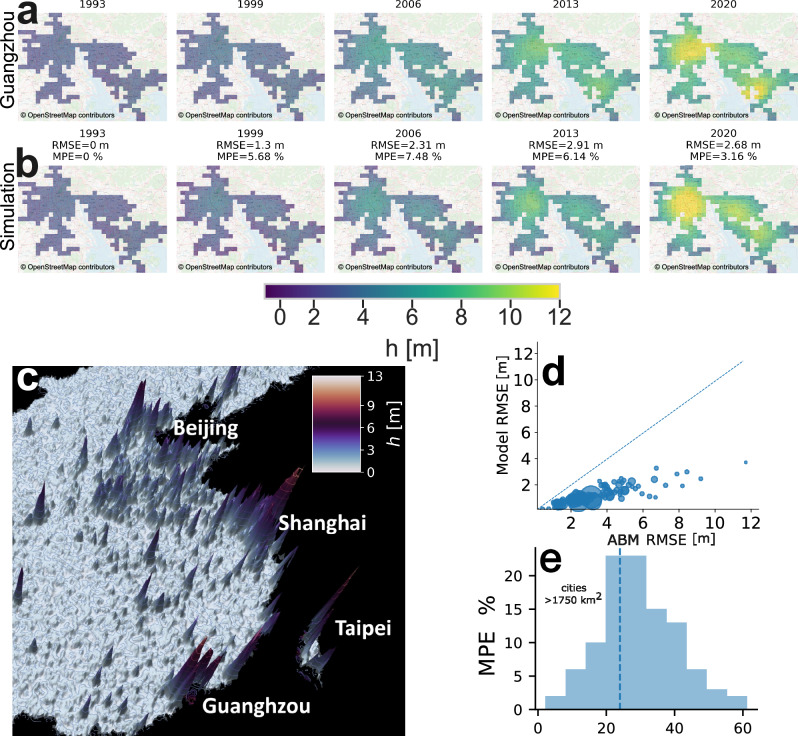
Geometric Brownian Motion (GBM) with local interactions simulation. Results of the model in Eq. (1), initialized in 1993 with parameters (*μ*, *σ*, *k*) optimized to minimize the overall Root Mean Squared Error (RMSE). Guangzhou building-height data (**a**) are compared with model simulations (**b**). Panel (**c**) shows the simulated final state (year 2020) for all cities in China and neighboring regions. Panel (**d**) summarizes model performance by comparing per-city RMSE values with those of an Arithmetic Brownian motion baseline. Dot size is proportional to the total city area. **e** reports the Mean absolute Percentage Error (MPE) of the fitted model (Eq. (1)); for the largest cities, the MPE is below 25%. Background map data in panels (**a**, **b**): © OpenStreetMap contributors, available under the Open Database License;^82^.

To evaluate the predictive performance of Eq. (1), we performed numerical simulations seeded with the 1993 building-height spatial distribution (see Methods for details). Across Chinese cities, the simulations reproduce the observed spatiotemporal dynamics well (Fig. 3), as reflected by the Root Mean Squared Error (RMSE) and Mean Absolute Percentage Error (MPE) values. These results should, however, be interpreted cautiously because the initial conditions already encode much of the spatial structure, effectively acting as quenched disorder. Consistent with this, calibration selects a relatively small noise amplitude *σ*, which minimizes the mismatch between simulations and observations (Supplementary Fig. 2). As a baseline, we compared our model against an Arithmetic Brownian motion (ABM) null model — i.e., a constant-drift process with additive Brownian noise. The fitted interaction model yields low percentage errors for the largest cities, with MPE below 25% (Fig. 3d, e). Smaller cities tend to have higher MPE because lower typical heights (smaller denominators) and a narrower dynamic range make percentage-based errors more sensitive to absolute deviations and noise.

**Fig. 4:**
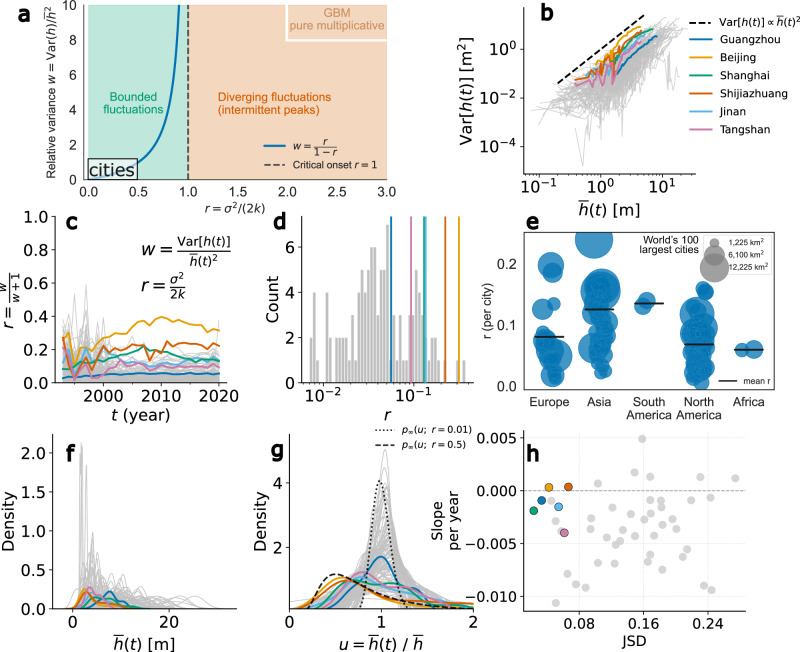
Growth regimes. **a** Regime diagram showing the competition between multiplicative noise and mean-field coupling in the stochastic growth model; **b** temporal evolution of variance versus mean; **c** time evolution of the relative fluctuation ratio *r*(*t*) = *w*(*t*)/(*w*(*t*) + 1); **d** distribution of *r* from Eq. (11) (*r* = *lim*_*t*→*∞*_*r*(*t*) in panel (c)). **e** Empirical value of *r* computed via Eq. (11) for cities worldwide. **f** Probability density functions (PDFs) of building heights computed from 5km-resolution data for 2020; **g** for the same cities, PDFs of the normalized height *u*, together with the theoretical PDF from Eq. (12); **h** Jensen-Shannon divergence (JSD; base 2) between the empirical PDFs of *u* in the last and previous year along with the annual slope of the JSD relative to the fitted stationary inverse-gamma distribution (negative indicates convergence) for cities larger then 1250 km^2^. Lower-left indicates strongest convergence; Panels (b-d and f-h) show results for East Asian cities, cities are represented by a gray line (or a dot in panel **h**), except for several example cities highlighted in color.

By employing a mean-field approximation (Eq. (6); see Methods), we can then characterize distinct growth regimes dependent on the balance between multiplicative noise and local spatial coupling, represented by the dimensionless parameter *r* = *σ*^2^/(2*k*) (see Methods). The regime diagram in Fig. 4a illustrates how existing urban morphologies emerge from the dynamic interplay between noise-driven stochastic fluctuations and local interaction-driven damping. Consistent with this picture, the intra-urban variance of building height scales with the city-average height (Fig. 4b; Eq. (11)). In addition the relative fluctuation $w(t)=\,{\rm{Var}}\,[h(t)]/{\overline{h}}^{2}(t)$) provides a practical estimator of *r* in the long-time limit for each city (see Fig. 4c–e and Methods).

Under the same mean-field approximation (Eq. (6)), the stationary probability density *p*_*∞*_(*u*), of the normalized height $u=h/{\overline{h}}$ can be derived (see Eq. (12) in the Methods section). This stationary law depends only on *r* (Eq. (9)), implying that, in this limit, all city-specific details such as shape or initial conditions become irrelevant. Fig. 4f and g show the empirical distributions of building height for Chinese cities and their normalized counterparts; for reference, curves of *p*_*∞*_(*u*) are plotted for two illustrative *r* values. To assess convergence toward *p*_*∞*_(*u*), the annual trend (slope) of the Jensen-Shannon divergence (JSD;^40^) between the empirical PDF of *u* and *p*_*∞*_(*u*) (with *r* inferred as above) is estimated. Figure 4h juxtaposes the year 2020 JSD with this annual slope: cities in the lower-left quadrant (small 2020 JSD and negative slope) provide the strongest evidence of convergence toward the theoretical stationary distribution. Notably, large cities such as Guangzhou, Beijing, and Shijiazhuang are already close to stationarity and show little additional convergence over 1993–2020, indicating they were close to the stationary regime from the outset.

**Fig. 5:**
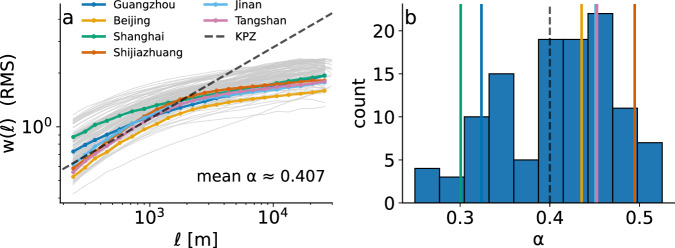
Multi-scale roughness and Kardar-Parisi-Zhang (KPZ)-type scaling across the study cities. **a** Root-mean-square roughness $w(\ell )=\sqrt{{\langle {(\phi -{\langle \phi \rangle }_{\ell })}^{2}\rangle }_{\ell }}$ versus box size *ℓ* on log--log axes. Thin gray curves show all cities; colored lines highlight example cities. The roughness exponent *α* is estimated from the pre-plateau regime. A dashed black reference line (labeled KPZ) indicates *w*(*ℓ*) ∝ *ℓ*^0.4^. **b** Distribution of *α* across cities; vertical markers denote the highlighted cases. Roughness exponent computed from the 30-m resolution data from^41^ for the year 2020.

Finally, we bridge our framework for urban growth modeling with the well-established literature on the physics of growing surfaces. Applying the Hopf-Cole transformation to the continuum limit of our model yields the KPZ equation (see Eq. (17) and Methods), which is a standard model for non-equilibrium interface growth and kinetic roughening^37^. We empirically explore this theoretical link using high-resolution urban surface height data from Chinese cities in 2020^41^, under the assumption of saturation conditions, consistent with the approach in ref. ^25^ but for the log-transformed height field. As illustrated in Fig. 5, we computed the local width *w*_*ϕ*_(*ℓ*) of the Hopf-Cole transformed surface $\phi=\ln (h)$ for each cities for different box size *ℓ*. We found an empirical roughness scaling *w*_*ϕ*_(*ℓ*) ∝ *ℓ*^*α*^ distributed around *α* ≈ 0.4. This result is consistent, for most of the studied cities, with (2 + 1)-dimensional KPZ predictions^42^, although temporal constraints in data availability prevent explicit determination of KPZ dynamic exponents.

## Discussion

A guiding principle of this work is to match model complexity to the information content of the data. The global building-height product resolves cities at a coarse spatial scale (roughly 5 × 5 km^2^), so the empirical object is a coarse-grained height field *h*(**x**, *t*) rather than a microscopic interface with identifiable deposition events. At this resolution, fitting fully microscopic surface-growth models (e.g., Restricted Solid-On-Solid, Eden, Asymmetric Simple-Exclusion Process^43^) would require an essentially arbitrary mapping from site-level rules to pixel-averaged heights and, in practice, embedding large simulations within a city-by-city likelihood. More importantly, many distinct microscopic mechanisms can generate indistinguishable coarse-grained statistics, making inference at the rule level weakly identifiable. We therefore adopted the opposite strategy: we focused on robust observables directly accessible in the data (log-returns, growth of moments, spatial roughness, and correlations) and identified the simplest analytically tractable stochastic evolution equation that reproduces them, using it as an effective description of urban vertical and horizontal growth at the resolved scale.

From a stochastic-growth perspective, modeling building height as a positive accumulated field subject to multiplicative noise is natural. In contrast to classical surface-growth equations (EW/KPZ-type), where additive noise injects fluctuations of fixed absolute amplitude directly into *h*, urban development is more plausibly driven by shocks that are effectively proportional to the current stock (relative changes in development intensity, investment, demand, or regulatory pressure). This choice yields scale-dependent fluctuations with a built-in positivity constraint (i.e., no ad hoc reflecting boundary at *h* = 0).

In the Supplementary Information (Supplementary Note), we further assess the robustness of our GBM framework by systematically comparing alternative stochastic models and alternative choices of “clock”. Specifically, we compared the GBM model to a simple null hypothesis, an Arithmetic Brownian Motion corresponding to a linear trend with constant (additive) noise, and we repeated the inference using three clocks: calendar time, total population, and log population. In urban science, population is commonly adopted as a natural clock to relate diverse urban quantities (e.g., GDP, road length, patents/crime, built area) through cross-sectional scaling relations^26,44,45^. Across the ensemble of cities, Akaike Information Criterion (AIC;^46^) model selection shows that GBM with calendar time provides the best overall description of height dynamics, while population-based clocks perform systematically worse, thereby supporting calendar time as the appropriate dynamical clock and GBM as a meaningful improvement over the null model.

This finding is consistent with the distinction emphasized by ref. ^47^ between cross-sectional scaling relations (where population acts as a size variable) and within-city temporal dynamics, for which using population as a clock can confound intensive time trends with demographic change and lead to unstable or biased temporal estimates. Moreover, population capacity depends on the vertical built form, so using population as a clock implies indexing one endogenous state variable by another, rather than by an external (and more natural) time parameter. Consistently, in the Supplementary Note, we showed that for individual cities both mean height and population grow approximately exponentially in calendar time, so that the apparent longitudinal relation ${\bar{H}}_{c}\propto {N}_{c}^{{\beta }_{c}^{{\rm{traj}}}}$ is purely kinematic, with exponent ${\beta }_{c}^{{\rm{traj}}}={\mu }_{c}/{\eta }_{c}$, i.e., simply the ratio of the height and population growth rates. This longitudinal exponent is therefore conceptually distinct from the familiar cross-sectional scaling exponent measured across cities at a fixed date, further supporting calendar time, rather than population, as the appropriate dynamical clock for within-city growth.

In general, our analysis shows that the vertical growth and internal structure of cities emerge from a delicate balance between multiplicative noise, which amplifies local fluctuations (a process called fluctuation-amplification by Bettencourt^31^), and spatial interactions, which diffuse those fluctuations across neighboring sites (Fig. 4). This mechanism offers an alternative to the migration terms commonly used in population-exchange models of interacting cities to prevent pure localization^4,27,39^, achieving stabilization through fluctuation–diffusion in the physical fabric rather than through exogenous flows.

Once local interactions are coarse-grained to the national scale — treating each city as a single point represented by its time-averaged mean building height — their stabilizing effects largely cancel out and the resulting aggregate trajectory of average building heights is well captured by a pure geometric Brownian motion (Eq. (2)). Strikingly, the same GBM description applies to city GDP, and the mean building height $\overline{h}(t)$ remains tightly correlated with economic output (Fig. 2).

At finer resolution, our spatially-extended model (Eq. (1)) reproduces the observed variance scaling (${\rm{Var}}[h(t)]\propto {\overline{h}}^{2}(t)$; Fig. 1b) and morphology of individual Chinese cities (Fig. 3), confirming that local interaction terms are essential for describing realistic intra-urban structures. This multi-scale construction shows that our framework remains coherent and predictive from neighborhoods to entire countries. This empirical and analytical results provide an explicit example of “self-similar growth" mechanisms that apply across scale (as introduced by^31^).

A practical outcome of these results is the empirical validation of building height as a proxy for the spatial distribution of economic activity. The observed tight $\overline{h}$–GDP coupling (Fig. 1g) provides evidence for the stochastic dynamics of economic growth in time-space as proposed by^48^]. It also suggests that remote-sensing height products can be deployed to monitor sub-national economic dynamics in data-scarce regions. Moreover, the dimensionless control parameters *r* = *σ*^2^/(2*k*) and *γ* = *μ* − *σ*^2^/2 (see Methods, Eq. (9) and Eq. (4)) provide parsimonious indicators of where a city falls on the spectrum from noise-dominated intermittent growth to diffusion-dominated near-deterministic expansion.

Our model, in the mean-field approximation, admits an analytical expression for the stationary distribution of normalized height (Eq. (12); empirical validation shows in Fig. 4). This stationary law depends only on *r* and any dependence on initial conditions vanishes asymptotically. Consequently, all sufficiently mature cities (i.e., those close to stationarity) that share the same *r* exhibit the same universal stationary distribution. This result provides an example of how universal behaviors emerge from the space-time dynamics of cities, providing a theoretical basis for recent work on urban structure and metabolism^49–51^, which finds that intra-urban properties collapse onto universal distributions once appropriately rescaled.

Because our observations are coarse-grained, the parameters of Eq. (1) should be interpreted as effective quantities: they summarize, at the resolved scale, the net outcome of many microscopic mechanisms (urban planning, financing, technology, regulation, geography, and infrastructure) rather than mapping one-to-one to a single causal driver. Within this reduced-form view, the drift *μ* sets the typical rate of vertical intensification (a proxy for the average strength of incentives to add floor space, shaped by demand, land values, productivity and profitability), while the multiplicative noise amplitude *σ* quantifies the strength of relative shocks to local development intensity (capturing boom–bust episodes, heterogeneous investment and timing, policy uncertainty, or other multiplicative fluctuations). The coupling strength *k* controls the spatial relaxation of height gradients through the diffusive term *k*∇^2^*h*: large *k* corresponds to stronger spatial integration and faster redistribution of local perturbations (e.g., via transport connectivity, shared infrastructure, and market arbitrage), whereas small *k* implies weaker coupling and more persistent local heterogeneity.

These effective parameters also admit a direct qualitative connection to empirical typologies of urban growth. Mahtta et al.^52^ classify urban pixels using a four-dimensional descriptor (initial horizontal state, initial vertical state, and the changes Δ*H* and Δ*V*) and identify five generic modes (stabilized, outward, mature upward, budding outward, and upward & outward^52^). Eq. (1) provides a minimal dynamical mapping onto these typologies which can be recovered by varying (*μ*, *k*, *σ*) together with the initial condition (see Supplementary Fig. 15). Specifically, *μ* primarily controls vertical intensification (shifting mass toward larger Δ*V*), *k* promotes lateral propagation/smoothing and thus horizontal spread (larger Δ*H*), and *σ* increases intermittency and patchiness (favoring fragmented, “budding” outward patterns).

In the continuum limit, our multiplicative-noise growth model (Eq. (1)) naturally connects to KPZ physics after a log transform: while the building-height field *h* evolves with multiplicative fluctuations, the transformed field $\phi=\ln h$ follows an additive-noise KPZ-like dynamics (Eq. (17)). This motivates measuring roughness for *ϕ*, rather than for *h*. Using the 30 m dataset^41^ (the 1993–2020 series^36^ is too coarse for this purpose), we quantify roughness via the local width *w*_*ϕ*_(*ℓ*) by tiling each height map with square boxes of side *ℓ* and computing within-box fluctuations (see Supplementary Method), yielding *w*_*ϕ*_(*ℓ*) ~ *ℓ*^*α*^. We find *α* values clustered around the KPZ expectation across most cities. We emphasize that this KPZ-like scaling pertains to $\phi=\ln h$, not directly to *h* itself, in contrast to^25^.

In the KPZ literature one often instead estimates *α*_global_ from the global saturated width ${w}_{{\rm{sat}}}(L) \sim {L}^{{\alpha }_{{\rm{global}}}}$ after establishing saturation at a system size *L*; for cities, however, a robust definition of *L* is hindered by finite, irregular, and heterogeneous domains (multiple centres, coastlines, discontinuities, and masking), and sparse temporal sampling makes saturation difficult to assess consistently across the sample. For these reasons we focus on local scaling (as also in ref. ^25^). While local and global roughness exponents can differ under anomalous roughening^53^, the simplest interpretation consistent with our data does not require invoking such additional complexity. Although the current four-epoch sampling is too sparse to test full dynamic Family–Vicsek scaling^54^, the consistency of *α* supports the idea that urban skylines may share universal roughening statistics with non-equilibrium growth; annual high-resolution height maps would enable a decisive test.

Overall, this work anchors the observed dynamic roughening of cities to a general theoretical framework. However, our findings must be interpreted in light of several limitations.

First, our model is most accurate for fast-growing, expanding cities that have not yet exhausted their vertical capacity. Mature metropolises, such as those in the United States, show clear deviations in building-height variance behavior from geometric Brownian motion (GBM), with an early onset of height dispersion followed by a plateau (Supplementary Fig. 6). This behavior is more naturally captured by the stochastic Fisher-Kolmogorov equation (see Methods; Eq. (18)) and Supplementary Fig. 12), which generalizes the heat equation used here with a carrying capacity *K* and produces a fluctuating saturated state. sFKPP connects directly to established urban-growth modeling practice through its logistic reaction term, which is the canonical coarse-grained representation of density-dependent growth with saturation. Logistic (S-shaped) dynamics have long been used in urban science to describe constrained expansion across scales, from aggregate urbanization trajectories that tend to accelerate and then plateau as structural limits are approached, to spatial models where local growth slows as a site-specific capacity is filled^55,56^. In this interpretation, the carrying capacity *K* is not a literal hard limit but an effective (coarse-grained) vertical capacity: it summarizes the bundle of constraints that progressively inhibit further densification, including economic feasibility, infrastructure and accessibility, construction technology, and planning or regulatory ceilings. A key advantage of this formulation is that it generalizes naturally to heterogeneous environments via a spatially varying capacity *K*(*x*), allowing the model to encode place-specific constraints, e.g., zoning and height limits, protected or flood-prone zones, discontinuous land availability, or differential provision of services—so that saturation emerges locally rather than uniformly across the city^56^. Looking ahead, an interesting perspective would be to develop a fully micro-founded description of city-wide expansion fronts: in such a framework, *K* (or *K*(*x*)) would be derived explicitly from underlying land and vertical-development constraints, and the resulting theory would predict not only the existence of invasion fronts but also their shape and propagation speed across heterogeneous urban space.

Second, our empirical analysis is confined largely to East-Asian cities; longer time series (covering periods of significant growth) for European and U.S. cities, and the inclusion of additional emerging regions, would be required to confirm the cross-regional validity of our approach. Third, it would be interesting to also take into account population density: explicitly modeling coupled population–building height dynamics could reveal how horizontal and vertical growth interact and disentangle key differences in residential/workplace occupancy^57^.

Finally, in this study, we compare the measured roughness exponents primarily to the KPZ universality class, but several other well-studied kinetic-roughening universality classes exist in 2+1 dimensions, which can yield similar exponents *α*. In particular, in the presence of quenched disorder, driven depinning problems define distinct “quenched” universality classes: the quenched KPZ (qKPZ) class at depinning has been reported with *α* ≃ 0.49 and *β* ≃ 0.42 in 2 + 1d^58^, values that fall within our observed range. For completeness, other canonical 2 + 1d classes yield markedly different roughening behavior: the linear Edwards–Wilkinson equation (KPZ without the nonlinearity) is marginal in 2 + 1d and produces logarithmic roughening (effectively *α* = 0 with *z* = 2)^59^, whereas conserved surface-diffusion dynamics of Mullins–Herring type yields *α* = 1 and *z* = 4^60^. A quenched Edwards–Wilkinson (QEW) depinning class is often characterized by a larger roughness exponent in *d* = 2 (typically reported around *α* ≃ 0.75)^61^. KPZ with more exotic noise term as also been studied as temporally^62^ and spacially^63^ correlated noise leading to further modifications of the original universality class. Higher-resolution spatiotemporal data spanning longer time windows will be needed to distinguish KPZ from alternative (including quenched) universality classes and to identify possible crossovers

Looking forward, it will be fruitful to generalize the proposed mathematical framework to heterogeneous diffusion constants that reflect transport infrastructure, to incorporate non-Gaussian construction shocks, and to test whether the building height field indeed satisfies full KPZ universality once extended space-time data become available. Such developments would deepen our understanding of how economic forces, planning regulations, and stochasticity ultimately shape the three-dimensional structure of modern cities. This knowledge will be crucial to anticipate - and eventually regulate - future housing dynamics, building material stocks, embodied carbon emissions, and urban-induced modifications of local microclimates^64–67^.

## Methods

### Theoretical framework

We model the evolution of building height *h*(*t*) at each spatial location as a stochastic multiplicative process. Initially, we consider heights described by geometric Brownian motion^68^:

2 $${\rm{d}}h=\mu h\,{\rm{d}}t+\sigma h\,{\rm{d}}W(t),$$

where *μ* represents deterministic drift linked directly to local GDP growth (Fig. 1g), *σ* characterizes multiplicative noise amplitude, and *W*(*t*) denotes standard Brownian motion. The PDF of *h*(*t*) with initial condition *h*(0) = *h*_0_ is the log-normal distribution

3 $$p(h(t))=\frac{1}{h(t)\,\sigma \sqrt{2\pi t}}\exp \left[-\frac{{\left(\ln(h(t)/{h}_{0})-(\mu -\frac{1}{2}{\sigma }^{2})t\right)}^{2}}{2{\sigma }^{2}t}\right].$$

For convenience, we define the effective drift

4 $$\gamma \,\equiv \,\mu -{\sigma }^{2},$$

when *γ* > 0 the drift of lnh(t) is strictly positive (see e.g.^31^), ensuring that every sample path are growing in the long run. This model provides an analytically tractable representation of proportional stochastic growth and is widely used across different domains (from finance^69^ to hydrology^70^ and city science^31^).

To introduce realistic spatial structures, we extend the GBM model onto a two-dimensional lattice, incorporating spatial interactions via diffusive coupling between neighboring sites. The height *h*_*i*_(*t*) at lattice site *i* evolves according to:

5 $${\rm{d}}{h}_{i}=\mu {h}_{i}\,{\rm{d}}t+\sigma {h}_{i}\,{\rm{d}}{W}_{i}(t)+k\mathop{\sum }\limits_{i{\prime} \in {\mathcal{N}}(i)}({h}_{i{\prime} }-{h}_{i})\,{\rm{d}}t,$$

where coupling strength *k* controls the intensity of local spatial interaction, and ${\mathcal{N}}(i)$ denotes the neighbors of site *i*.

To gain analytical insight, we adopt a mean-field approximation, replacing explicit neighbor interactions with coupling to the mean height $\overline{h}(t)={\mathbb{E}}[h(t)]$. Thus, the mean-field dynamics reduce to^34^:

6 $${\rm{d}}h=(\mu -k)h\,{\rm{d}}t+k\overline{h}(t)\,{\rm{d}}t+\sigma h\,{\rm{d}}W(t).$$

Therefore, the mean is (see Supplementary Method for details):

7 $$\overline{h}(t)=\overline{h}(0){e}^{\mu t};$$

where $\overline{h}(t)$ also corresponds to the mean behavior of uncoupled GBM. It follows that the variance is (for *k* ≠ 0):

8 $${\rm{Var}}[h(t)]=\frac{r}{1-r}\overline{h}{(t)}^{2}\left(1-{e}^{-2k(1-r)t}\right),$$

with *r* an adimensional parameter:

9 $$r={\sigma }^{2}/(2k),$$

governing the system behavior. When *k* = 0 we recover the variance of the GBM process:

10 $${\rm{Var}}[h(t)]=\overline{h}{(t)}^{2}\left({e}^{{\sigma }^{2}t}-1\right).$$

For *k* > 0, three regimes can be distinguished according to the value of *r*. When *r* < 1, coupling dominates over noise. In this case, the variance grows proportionally to ${\overline{h}}^{2}$, so that the relative variance approaches a finite asymptotic value:

11 $$w(t):=\frac{{\rm{Var}}[h(t)]}{{\overline{h}}^{2}}\,\mathop{\longrightarrow}\limits_{t \to \infty}\,\frac{r}{1-r}.$$

This regime provides a good characterization of the cities considered in this study, as shown in Figs. 1b and 4b. By contrast, when *r* > 1, noise dominates over coupling, leading to GBM-like behavior. The variance then grows faster than ${\overline{h}}^{2}$, and the relative variance becomes unbounded, indicating an instability driven by noise fluctuations. The marginal case *r* = 1 separates these two regimes. At criticality, the variance grows as $t\,\overline{h}{(t)}^{2}$.

These different regimes are summarized in the diagram reported in Fig. 4a (finite time and finite size generic simulation illustrating these regimes are provided in the Supplementary Fig. 7).

The exact Fokker–Planck equation associated with Eq. (6) is not analytically solvable (this is even more true for the non-approximated model in Eq. (1)). However, the normalized variable $u: \!\!=h/\overline{h}$ admits a stationary PDF *p*_*∞*_ in the mean-field approximation that is (See Supplementary Method and^34^):

12 $${p}_{\infty }(u)=\frac{{(1/r)}^{1+1/r}}{\Gamma \left(1+\frac{1}{r}\right)}\,{u}^{-\left(2+\frac{1}{r}\right)}\,\exp \left(-\frac{1}{r\,u}\right).$$

The characteristic relaxation time *τ* for the distribution of the normalized variable *u* to reach *p*_*∞*_(*u*) is given by

$$\tau=\frac{1}{2k(1-r)}\,(r < 1).$$

This timescale emerges from the exponential relaxation of the relative variance $w(t)={\rm{Var}}[h(t)]/{\overline{h}}^{2}$ to its asymptotic value *r*/(1 − *r*) (see Eq. (11)). Physically, *τ* quantifies how quickly spatial coupling (*k*) restrains noise-induced fluctuations (*σ*) to establish a stable relative inequality pattern among cities. When *r* → 1, the relaxation diverges, marking the transition to a noise-dominated regime where no stationary distribution exists.

Note that the tail of the distribution can be approximated as a Pareto power:

13 $${P}_{\infty }(u)\approx {u}^{-2+1/r}.$$

We can express the PDF of *h* as:

14 $$p(h(t))=\frac{1}{\overline{h}(t)}\,p\left(\frac{h(t)}{\overline{h}(t)}\right).$$

If the normalized process *u*(*t*) has reached stationarity, the family {*p*(*h*(*t*))}_*t*_ becomes self-similar: its shape is fixed (given by *p*_*∞*_) while its scale evolves as $\overline{h}(t)$. Before stationarity, however, the shape remains time-dependent, and self-similarity holds only asymptotically:

15 $$p(h(t))\mathop{\longrightarrow}\limits_{t \to \infty}\frac{1}{\overline{h}(t)}\,p_\infty\!\left(\frac{h}{\overline{h}(t)}\right).$$

This stationary distribution of the normalized field *u*(*t*) provides a simple mechanism for the emergence of universal (time-invariant) probability density functions from a dynamical model, a behavior reported and observed in various studies^4,49–51^.

To understand urban morphology at larger scales, we study the continuum limit of the spatially coupled GBM. In this limit, the model follow the stochastic heat equation with multiplicative noise (SHE)^71,72^:

16 $${\partial }_{t}h=D{\nabla }^{2}h+\mu h+\sigma h\eta ({\bf{x}},t),$$

where *D* = *ka*^2^ (for small lattice spacing *a* → 0) denotes an effective diffusion coefficient and *η*(**x**, *t*) is Gaussian white noise. Applying the Hopf-Cole transformation $h({\bf{x}},t)=\exp [\phi ({\bf{x}},t)]$^73^, SHE converts to the Kardar-Parisi-Zhang equation^37^:

17 $${\partial }_{t}\phi=D{\nabla }^{2}\phi+\frac{\lambda }{2}{(\nabla \phi )}^{2}+\mu+\sigma \eta ({\bf{x}},t),$$

with canonical nonlinearity *λ* = 2*D* (the drift term *μ* can be absorbed by redefining the field *ϕ* through a Galilean transformation). This explicitly connects our urban growth model to the KPZ universality class, thereby grounding urban spatiotemporal dynamics within a rigorous statistical physics framework. The KPZ equation was initially developed to describe growth through random deposition and diffusion; it characterizes a universality class thought to include a wide range of models and physical systems^43^.

It should be emphasized that the KPZ correspondence concerns the transformed field $\phi=\ln h$ induced by our multiplicative-noise dynamics, and does not imply that the height field *h* itself follows a standard additive-noise KPZ equation. This distinction is supported by a simple temporal diagnostic. For a 2 + 1D KPZ interface with drift, one expects

$${\mathbb{E}}[h(t)]\simeq {v}_{\infty }t,\quad\,{\rm{Var}}(h(t))\propto {t}^{2\beta },\,\quad \beta \approx 0.24\,{\rm{in}}\; ({\rm{2}}\;+\; 1){\rm{d}}\,,$$

so that the squared coefficient of variation behaves as

$$\frac{{\rm{Var}}(h(t))}{{\mathbb{E}}{[h(t)]}^{2}}\,\simeq \,\frac{C\,{t}^{2\beta }}{{({v}_{\infty }t)}^{2}}\,\propto {t}^{-1.52},$$

i.e., additive-noise KPZ dynamics predict a rapid decay of relative fluctuations in *h*. Empirically, however, we observe that ${\rm{Var}}(h(t))/{\mathbb{E}}{[h(t)]}^{2}$ approaches a positive constant as *t* increases (Fig. 4c). This behavior is consistent with multiplicative (GBM-like) fluctuations for *h*, and it motivates formulating KPZ-type roughening at the coarse-grained scale in terms of the logarithmic field $\phi=\ln h$ rather than the raw height field *h*.

Beyond the stochastic heat equation, the stochastic Fisher-Kolmogorov-Petrovsky-Piscunov (sFKPP;^74^) equation offers a natural generalization for systems that approach a finite carrying capacity *K*. Indeed, a soft saturation scale *K* can be introduced via a logistic drift by generalizing the drift parameter *μh* by *μh*(1 − *h*/*K*). In two spatial dimensions, it reads

18 $${\partial }_{t}h=D{\nabla }^{2}h+\mu \,h\left(1-\frac{h}{K}\right)+\sigma \,h\,\eta ({\bf{x}},t),$$

where the logistic term drives the system toward a fluctuating plateau at *h* ≈ *K*. This regime is conceptually well suited to model mature, land-limited cities that are close to their maximum vertical capacity (Supplementary Fig. 12 shows a monocentric numerical simulation of sFKPP). The deterministic FKPP equation is well known to generate reaction–diffusion invasion fronts with an asymptotic traveling-wave profile. For sufficiently steep initial conditions, it selects a pulled speed set by the linear growth rate and diffusion. The multiplicative noise in the sFKPP perturbs these fronts: it produces stochastic fluctuations in the front location and, for compact-support-type initial data, selects an asymptotic propagation speed *c*(*σ*). Here, *c*(0) denotes the deterministic FKPP speed (the *σ* → 0 limit), and it holds that *c*(*σ*)≤*c*(0)^74,75^. In this sense, the noise renormalizes (and typically reduces) the effective front speed while inducing front wandering around the mean drift. In our simulations, we initialize several “seed” centres of development (high *h*) embedded in a low-height noisy background. Consequently, the dynamics naturally produces multiple interacting fronts whose expansion, merger, and stabilization capture the growth and coalescence of urban subcentres.

### Dataset description

We used the global, 1567 cities dataset of^36^, which merges observations from three C-band Ku-band radar scatterometers (ERS-1/2, QuikSCAT, ASCAT) with Landsat-derived settlement fractions to approximate fractional building cover and volumetric backscatter. Annual composites are provided on a 0.05^∘^ × 0.05^∘^ grid ( ≈ 5km resolution) for 1993–2020, that gives two complementary proxies of vertical development: (i) microwave backscatter, correlated with built volume, and (ii) settlement built-up fraction, correlated with planimetric expansion. City boundaries follow the Morphological Urban Area (MUA) polygons from^76^, as adopted to define 1567 urban areas across 151 countries. Each MUA polygon was overlaid with a 0.05^∘^ lat/lon grid, and all grid cells contained within or intersecting the polygon were assigned to the city. Grid cells with ≥50% open water were masked using the Global 1-km Consensus Land Cover dataset, and MUAs lacking required inputs were excluded.

To calibrate and homogenize the radar-optical series over Chinese cities, we employed the 30 m China Multi-Temporal Built-up Height product (CMTBH-30^41^). CMTBH-30 provides per-pixel mean building height for the benchmark years 2005, 2010, 2015, and 2020, derived with the MTBH-Net algorithm that uses GEDI lidar returns, Landsat spectral indices, ALOS/PALSAR backscatter, and change metrics (overall RMSE < 6.5 m).

Gross Domestic Product (GDP) and population for U.S. metropolitan statistical areas (MSAs) are taken from the Federal Reserve Bank of St.Louis economic database^77^. Corresponding prefecture-level GDP and population series for China are obtained from the National Bureau of Statistics of China^78^.

### Data processing

The C-/Ku-band backscatter are converted in decibels to unitless power-return ratio^36^ on the native 0.05^∘^ grid using May composites only, remove non-positive values and incomplete metadata, and re-index time so that *t* = 0 is the first observation for each city. Satellites cover partially overlapping periods (ERS 1993-2000, QuikSCAT 1999-2009, ASCAT 2007-2021).

To place all satellites on a common scale, we perform a per-pixel intercalibration with QuikSCAT as the reference. For pixels observed by ASCAT and QuikSCAT in 2007-2009, we estimate a pixel-specific affine mapping on the log scale using only the overlap years and apply it to all ASCAT years. Because the ERS-QuikSCAT overlap (1999–2000) is very short, we use a per-pixel intercept-only alignment for ERS, which preserves ERS pixel trends. We refer the reader to the Supplementary Method for further details.

For China, we convert the power-return ratio to building height using the CMTBH-30 product (30 m;^41^). We first aggregate CMTBH-30 to the 0.05^∘^ analysis grid by averaging all 30 m pixels within each cell, producing a gridded benchmark of building height for 2020 (see also Supplementary Fig. 9). For each city, we relate the 2020 May power-return ratio to the aggregated 2020 building height across all grid cells with both datasets available, using a simple city-specific linear calibration. The fitted city-level coefficients are then applied to the full time series of May power-return ratios to obtain annual building heights, in meters, for each year in the study period.

For cities outside China, we retain the reconstructed May power-return series ${h}_{c,i,t}^{\,{\rm{May}}}$ as the vertical-development proxy without converting to meters. This is appropriate because our GBM-based inference targets relative temporal change (log-drift *γ*) and volatility *σ*, both of which are invariant to multiplicative rescaling of *h*.

### Numerical simulations and calibration

We simulate Eq. (1) on a discrete 2D grid of cells whose values represent building heights. We used the Euler–Maruyama scheme^79^:

19 $${h}_{i}^{t+\Delta t}={h}_{i}^{t}+\mu \,{h}_{i}^{t}\,\Delta t+\sigma \,{h}_{i}^{t}\,\sqrt{\Delta t}\,{\xi }_{i}^{t}+k\,\Delta t\mathop{\sum }\limits_{j\in {\mathcal{N}}(i)}\left({h}_{j}^{t}-{h}_{i}^{t}\right).$$

with i.i.d. standard normal shocks ${\xi }_{i}^{t} \sim {\mathcal{N}}(0,1)$ and ${\mathcal{N}}(i)$ is the 8-nearest neighborhood. Heights are initialized from the first observation year (1993), ${h}_{i}^{{t}_{0}}={H}_{i}^{{\rm{obs}}}(1993)$.

Cities are simulated on fixed spatial domains, and cells outside the mapped urban extent are treated as inactive via a boolean mask. These cells are thus excluded from neighborhood sums in Eq. (19) and all loss calculations; this is equivalent to imposing no-flux boundary conditions along the domain perimeter.

For each city, parameters (*μ*, *σ*, *k*) are estimated by minimizing the mean RMSE between simulations and observations across all available years. For any candidate (*μ*, *σ*, *k*) we run *R* stochastic replicas and compare the ensemble mean ${\bar{h}}_{i}^{t}=\frac{1}{R}{\sum }_{r=1}^{R}{h}_{i}^{t,(r)}$ to the data over the active set Ω:

20 $${{\rm{RMSE}}}_{t}={\left(\frac{1}{| \Omega | }\mathop{\sum }\limits_{i\in \Omega }{\left({\bar{h}}_{i}^{t}-{H}_{i}^{{\rm{obs}}}(t)\right)}^{2}\right)}^{1/2},\,{\mathcal{L}}(\mu,\sigma,k)=\frac{1}{T}\mathop{\sum }\limits_{t=1}^{T}{{\rm{RMSE}}}_{t}.$$

We minimize ${\mathcal{L}}$ subject to *σ* ≥ 0 and *k* ≥ 0 using a coarse grid search followed by a local derivative-free optimizer based on a quasi-Newton method called Limited-memory BFGS^80^. Error metrics are computed only over active (non-masked) cells.

### Reporting summary

Further information on research design is available in the Nature Portfolio Reporting Summary linked to this article.

## Supplementary information

Supplementary Information

Reporting Summary

Transparent Peer Review file

## Data Availability

The radar–optical urban development dataset used in this study is publicly available from the global 1,567-city products described by^81^ and^36^. These data include the C-/Ku-band scatterometer backscatter time series from ERS-1/2, QuikSCAT and ASCAT, together with Landsat-derived settlement fraction estimates on a 0.05^∘^ × 0.05^∘^ grid. Morphological Urban Area boundaries are available from^76^. The open-water mask was derived from the Global 1-km Consensus Land Cover dataset. Building-height calibration for Chinese cities used the CMTBH-30 product of^41^. Gross Domestic Product and population data for U.S. metropolitan statistical areas were obtained from the Federal Reserve Bank of St. Louis database^77^. Prefecture-level Gross Domestic Product and population data for China were obtained from the National Bureau of Statistics of China^78^. All datasets used in this study are publicly accessible through the sources cited above.

## References

[1] Liu, X. et al. High-spatiotemporal-resolution mapping of global urban change from 1985 to 2015. *Nat. Sustainability* **3**, 564–570 (2020).

[2] Grimm, N. B. et al. Global change and the ecology of cities. *science* **319**, 756–760 (2008). 18258902 10.1126/science.1150195

[3] Bettencourt, L. M., Lobo, J., Helbing, D., Kühnert, C. & West, G. B. Growth, innovation, scaling, and the pace of life in cities. *Proc. Natl. Acad. Sci.* **104**, 7301–7306 (2007). 17438298 10.1073/pnas.0610172104 PMC1852329

[4] Verbavatz, V. & Barthelemy, M. The growth equation of cities. *Nature* **587**, 397–401 (2020). 33208958 10.1038/s41586-020-2900-x

[5] Barthelemy, M. The statistical physics of cities. *Nat. Rev. Phys.* **1**, 406–415 (2019).

[6] United Nations. World urbanization prospects 2025: Summary of results. Tech. Rep., UN DESA/POP/2025/TR/ No. 12, New York. Population Division, Department of Economic and Social Affairs (2025).

[7] Seto, K. C., Fragkias, M., Güneralp, B. & Reilly, M. K. A meta-analysis of global urban land expansion. *PloS one* **6**, e23777 (2011). 21876770 10.1371/journal.pone.0023777 PMC3158103

[8] Brueckner, J. K. Urban sprawl: Diagnosis and remedies. *Int. regional Sci. Rev.* **23**, 160–171 (2000).

[9] Baum-Snow, N. Did highways cause suburbanization? * Q. J. Econ.* **122**, 775–805 (2007).

[10] Capel-Timms, I., Levinson, D., Lahoorpoor, B., Bonetti, S. & Manoli, G. The angiogenic growth of cities. *J. R. Soc. Interface* **21**, 20230657 (2024). 38565159 10.1098/rsif.2023.0657 PMC10987239

[11] Burchfield, M., Overman, H. G., Puga, D. & Turner, M. A. Causes of sprawl: A portrait from space. * Q. J. Econ.* **121**, 587–633 (2006).

[12] Ahlfeldt, G. M. & Barr, J. The economics of skyscrapers: A synthesis. *J. Urban Econ.* **129**, 103419 (2022).

[13] Gabaix, X. Zipf’s law for cities: an explanation. * Q. J. Econ.* **114**, 739–767 (1999).

[14] Rozenfeld, H. D., Rybski, D., Gabaix, X. & Makse, H. A. The area and population of cities: New insights from a different perspective on cities. *Am. Economic Rev.* **101**, 2205–2225 (2011).

[15] Schläpfer, M., Lee, J. & Bettencourt, L. Urban skylines: building heights and shapes as measures of city size. *arXiv preprint arXiv:1512.00946* (2015).

[16] Alonso, W. *Location and land use: Toward a general theory of land rent* (Harvard University Press, 1964).

[17] Fischer, K. Central places: The theories of von Thünen, christaller, and lösch. In *Foundations of location analysis*, 471–505 (Springer, 2011).

[18] Krugman, P. *The self-organizing economy* (John Wiley & Sons, 1996).

[19] Batty, M. & Longley, P. A. The fractal simulation of urban structure. *Environ. Plan. a* **18**, 1143–1179 (1986).

[20] Frankhauser, P.*La fractalité des structures urbaines* (Anthropos, 1994).

[21] Batty, M. Cities as fractals: simulating growth and form. In *Fractals and chaos*, 43–69 (Springer, 1991).

[22] Makse, H. A. et al. Modeling urban growth patterns with correlated percolation. *Phys. Rev. E* **58**, 7054 (1998).

[23] Raimbault, J. Calibration of a density-based model of urban morphogenesis. *PloS one* **13**, e0203516 (2018). 30188949 10.1371/journal.pone.0203516 PMC6126859

[24] Tirico, M., Balev, S., Dutot, A. & Olivier, D. Morphogenesis of complex networks: a reaction diffusion framework for spatial graphs. In *International Conference on Complex Networks and their Applications*, 769–781 (Springer, 2018).

[25] Najem, S., Krayem, A., Ala-Nissila, T. & Grant, M. Kinetic roughening of the urban skyline. *Phys. Rev. E* **101**, 050301 (2020). 32575232 10.1103/PhysRevE.101.050301

[26] Marquis, U., Artime, O., Gallotti, R. & Barthelemy, M. Universal roughness and the dynamics of urban expansion. *Phys. Rev. Lett.* **135**, 187403 (2025). 41247965 10.1103/rlkq-t36n

[27] Reia, S. M., Rao, P. S. C., Barthelemy, M. & Ukkusuri, S. V. Spatial structure of city population growth. *Nat. Commun.* **13**, 5931 (2022). 36209135 10.1038/s41467-022-33527-y PMC9547901

[28] Jin, M., Wang, L., Ge, F. & Yan, J. Detecting the interaction between urban elements evolution with population dynamics model. *Sci. Rep.* **13**, 12367 (2023). 37524780 10.1038/s41598-023-38979-w PMC10390572

[29] Gibrat, R. Les inégalités économiques, librairie du recueil sirey, paris. *What makes firms grow in developing countries* **169** (1931).

[30] Eeckhout, J. Gibrat’s law for (all) cities. *Am. Economic Rev.* **94**, 1429–1451 (2004).

[31] Bettencourt, L. M. Urban growth and the emergent statistics of cities. *Sci. Adv.* **6**, eaat8812 (2020). 32875099 10.1126/sciadv.aat8812 PMC7438098

[32] Bonetti, S. & Porporato, A. On the dynamic smoothing of mountains. *Geophys. Res. Lett.* **44**, 5531–5539 (2017).

[33] Munoz, M. A. Multiplicative noise in non-equilibrium phase transitions: A tutorial https://arxiv.org/abs/cond-mat/0303650 (2003).

[34] Bouchaud, J.-P. & Mézard, M. Wealth condensation in a simple model of economy. *Phys. A: Stat. Mech. its Appl.* **282**, 536–545 (2000).

[35] Barr, J. & Luo, J. Growing skylines: The economic determinants of skyscrapers in china. * J. real. estate Financ. Econ.* **63**, 210–248 (2021).

[36] Frolking, S., Mahtta, R., Milliman, T., Esch, T. & Seto, K. C. Global urban structural growth shows a profound shift from spreading out to building up. *Nature Cities* 1–12 (2024).

[37] Kardar, M., Parisi, G. & Zhang, Y.-C. Dynamic scaling of growing interfaces. *Phys. Rev. Lett.* **56**, 889 (1986). 10033312 10.1103/PhysRevLett.56.889

[38] Wakita, J. -i, Itoh, H., Matsuyama, T. & Matsushita, M. Self-affinity for the growing interface of bacterial colonies. *J. Phys. Soc. Jpn.* **66**, 67–72 (1997).

[39] Bernard, M., Bouchaud, J. P., & Le Doussal, P. Mean-field theory for heterogeneous random growth with redistribution. *Phys. Rev. E*. **113**, L032101 (2026). 10.1103/nchz-flkj 41998918

[40] Lin, J. Divergence measures based on the shannon entropy. *IEEE Trans. Inf. theory* **37**, 145–151 (2002).

[41] Chen, P. et al. Characterizing dynamics of built-up height in china from 2005 to 2020 based on gedi, landsat, and palsar data. *Remote Sens. Environ.* **325**, 114776 (2025).

[42] Pagnani, A. & Parisi, G. Numerical estimate of the kardar-parisi-zhang universality class in (2+ 1) dimensions. *Phys. Rev. E* **92**, 010101 (2015). 10.1103/PhysRevE.92.010101 26274100

[43] Barabási, A.-L. & Stanley, H. E. *Fractal concepts in surface growth* (Cambridge University Press, 1995).

[44] Batty, M. The size, scale, and shape of cities. *science* **319**, 769–771 (2008). 18258906 10.1126/science.1151419

[45] Bettencourt, L. M. The origins of scaling in cities. *science* **340**, 1438–1441 (2013). 23788793 10.1126/science.1235823

[46] Cavanaugh, J. E. & Neath, A. A. The akaike information criterion: Background, derivation, properties, application, interpretation, and refinements. *Wiley Interdiscip. Rev.: Computational Stat.* **11**, e1460 (2019).

[47] Bettencourt, L. et al. The interpretation of urban scaling analysis in time. *J. Royal Society Interface* **17** (2020). 10.1098/rsif.2019.0846 PMC7061707 32019469

[48] Gozzi, F. & Leocata, M. A stochastic model of economic growth in time-space. *SIAM J. Control Optim.* **60**, 620–651 (2022).

[49] Hendrick, M., Rinaldo, A. & Manoli, G. A stochastic theory of urban metabolism. *Proc. Natl. Acad. Sci. USA* **122**, e2501224122 (2025). 40789025 10.1073/pnas.2501224122 PMC12377725

[50] Strano, E. et al. The scaling structure of the global road network. *R. Soc. open Sci.* **4**, 170590 (2017). 29134071 10.1098/rsos.170590 PMC5666254

[51] La Porta, C. A. & Zapperi, S. Urban scaling functions: Emission, pollution and health. *J. Urban Health* **101**, 752–763 (2024). 38997534 10.1007/s11524-024-00888-2 PMC11329451

[52] Mahtta, R., Mahendra, A. & Seto, K. C. Building up or spreading out? typologies of urban growth across 478 cities of 1 million+. *Environ. Res. Lett.* **14**, 124077 (2019).

[53] Ramasco, J. J., López, J. M. & Rodríguez, M. A. Generic dynamic scaling in kinetic roughening. *Phys. Rev. Lett.* **84**, 2199 (2000). 11017243 10.1103/PhysRevLett.84.2199

[54] Vicsek, T. & Family, F. Dynamic scaling for aggregation of clusters. *Phys. Rev. Lett.* **52**, 1669 (1984).

[55] Mulligan, G. F. Reprint of: Revisiting the urbanization curve. *Cities* **32**, S58–S67 (2013).

[56] Whiteley, T. D., Avitabile, D., Siebers, P.-O., Robinson, D. & Owen, M. R. Modelling the emergence of cities and urban patterning using coupled integro-differential equations. *J. R. Soc. Interface* **19**, 20220176 (2022). 35506210 10.1098/rsif.2022.0176 PMC9065963

[57] Batista e Silva, F. et al. Uncovering temporal changes in Europe’s population density patterns using a data fusion approach. *Nat. Commun.* **11**, 4631 (2020). 32934205 10.1038/s41467-020-18344-5 PMC7493994

[58] Tajuelo-Valbuena, Á., Trujillo-Mulero, J., Meléndez, J. J., Cuerno, R. & Ruiz-Lorenzo, J. J. Exponents and front fluctuations in the quenched Kardar-Parisi-Zhang universality class of one and two-dimensional interfaces. *arXiv preprint arXiv:2512.15366* (2025). 10.1103/zs28-q7nb 42141619

[59] Majaniemi, S., Ala-Nissila, T. & Krug, J. Kinetic roughening of surfaces: Derivation, solution, and application of linear growth equations. *Phys. Rev. B* **53**, 8071 (1996). 10.1103/physrevb.53.8071 9982265

[60] Darvish, E. & Masoudi, A. A. Kinetic surface roughening for the mullins–herring equation. *J.**Mathematical Phys.* **50** (2009).

[61] Makse, H. A., Buldyrev, S., Leschhorn, H. & Stanley, H. E. The pinning paths of an elastic interface. *Europhys. Lett.* **41**, 251 (1998).

[62] Katzav, E. & Schwartz, M. Kardar-parisi-zhang equation with temporally correlated noise: A self-consistent approach. *Phys. Rev. E—Stat., Nonlinear, Soft Matter Phys.* **70**, 011601 (2004). 10.1103/PhysRevE.70.011601 15324059

[63] Janssen, H., Täuber, U. C. & Frey, E. Exact results for the kardar-parisi-zhang equation with spatially correlated noise. * Eur. Phys. J. B-Condens. Matter Complex Syst.* **9**, 491–511 (1999).

[64] Kamath, H. G. et al. Global building heights for urban studies (ut-globus) for city-and street-scale urban simulations: Development and first applications. *Sci. Data* **11**, 886 (2024). 39147835 10.1038/s41597-024-03719-w PMC11327349

[65] Manoli, G. et al. Magnitude of urban heat islands largely explained by climate and population. *Nature* **573**, 55–60 (2019). 31485056 10.1038/s41586-019-1512-9

[66] Li, C., Pradhan, P., Chen, G., Kropp, J. P. & Schellnhuber, H. J. Carbon footprint of the construction sector is projected to double by 2050 globally. *Commun. Earth Environ.* **6**, 831 (2025). 41163641 10.1038/s43247-025-02840-x PMC12559003

[67] Zhang, C. et al. Building material stock drives embodied carbon emissions and risks future climate goals in China. *Nature Climate Change* 1–8 (2026).

[68] Ross, S. M. Variations on Brownian motion. *Introduction to Probability Models* 612–614 (2014).

[69] Farida Agustini, W., Affianti, I. R. & Putri, E. R. Stock price prediction using geometric brownian motion. In *Journal of Physics: Conference Series*, vol. 974, 012047 (IOP Publishing, 2018).

[70] Lefebvre, M. Geometric Brownian motion as a model for river flows. *Hydrological Process.* **16**, 1373–1381 (2002).

[71] Bertini, L. & Cancrini, N. The two-dimensional stochastic heat equation: renormalizing a multiplicative noise. *J. Phys. A: Math. Gen.* **31**, 615 (1998).

[72] Hu, Y. Some recent progress on stochastic heat equations. *Acta Mathematica Sci.* **39**, 874–914 (2019).

[73] Hairer, M. Solving the KPZ equation. *Annals of mathematics* 559–664 (2013).

[74] Doering, C. R., Mueller, C. & Smereka, P. Interacting particles, the stochastic fisher–kolmogorov–petrovsky–piscounov equation, and duality. *Phys. A: Stat. Mech. its Appl.* **325**, 243–259 (2003).

[75] Conlon, J. G. & Doering, C. R. On travelling waves for the stochastic fisher–kolmogorov–petrovsky–piscunov equation. *J. Stat. Phys.* **120**, 421–477 (2005).

[76] Taubenböck, H. et al. A new ranking of the world’s largest cities—do administrative units obscure morphological realities? (2019).

[77] Federal Reserve Economic Data. https://fred.stlouisfed.org/. Accessed: 2025-07-28 (2025).

[78] National bureau of statistics of china. https://www.stats.gov.cn/english/. Accessed: 2025-07-28 (2025).

[79] Kloeden, P. E., Platen, E. & Schurz, H. *Numerical solution of SDE through computer experiments* (Springer Science & Business Media, 2012).

[80] Liu, D. C. & Nocedal, J. On the limited memory bfgs method for large scale optimization. *Math. Program.* **45**, 503–528 (1989).

[81] Frolking, S. et al. A global urban microwave backscatter time series data set for 1993–2020 using ers, quikscat, and ascat data. *Sci. Data* **9**, 88 (2022). 35296666 10.1038/s41597-022-01193-w PMC8927099

[82] OpenStreetMap contributors. Planet dump retrieved from https://planet.osm.org. https://www.openstreetmap.org (2017).

