# Supplementary material for: Dynamic roughening of cities driven by multiplicative noise
Source: Nat Commun. 2026 Jun 25;17:7974. doi: 10.1038/s41467-026-74456-4 (PMC13448524; doi:10.1038/s41467-026-74456-4)
Supplement: Supplementary file 1 [file 41467_2026_74456_MOESM1_ESM.pdf]

Supplementary Information: Dynamic roughening of cities driven by multiplicative noise

Martin Hendrick[1,*] and Gabriele Manoli[1,*]

[1]Laboratory of Urban and Environmental Systems, École Polytechnique Fédérale de Lausanne, Lausanne, Switzerland

[*]Corresponding authors: gabriele.manoli@epfl.ch and martin.hendrick@epfl.ch

List of Supplementary Items

Supplementary Note **1**

Urban morphological properties . . . 1

GBM compared to null model and the choice of the clock . . . 3

Supplementary Method **6**

Stationary PDF of the spatially coupled GBM in the mean-field approximation . . . 6

Inter-satellite bias correction . . . 9

Distance to a Theoretical Stationary Law via Jensen–Shannon Divergence 11

Local-width roughness on a log–height field with masking and pre-plateau fitting . . . 12

Supplementary Figures **14**

Supplementary Note

Urban morphological properties

This section briefly documents additional morphological properties generated by Eq. 1, beyond the properties discussed in the main text.

1. **Typical radial decay of building height from a Central Business District.** Urban economics and empirical skyline studies typically report height profiles that peak at the main economic centre(s) and decrease with distance. In a simple monocentric numerical setting, we reproduce this stylized fact by initializing a single central "seed" (a localized height perturbation) on top of small background fluctuations and evolving Eq. 1. The resulting long-time configuration concentrates the tallest values near the seed, and the radially averaged profile $\overline{h}(d)$ decays smoothly with the distance d to the centre (Fig. 13). The same mechanism extends naturally to polycentric configurations (two or more seeds), yielding flatter radial averages when distances are measured to a global reference point, consistent with the intuition that multiple centres broaden the distribution of tall structures [1, 2].

2. **Height–height correlations (Batty-type long-range decay).** To connect with spatial correlation analyses of urban skylines, we compute a two-point (pair) correlation function $g(R)$ from the stationary height field, following the point-pattern approach used for London in [3]. Concretely, we threshold the simulated field at a level τ (mimicking the methodology used in [3] to select the "tallest" subset) and evaluate $g(R)$ on the resulting exceedance set using an annulus-based estimator. Over an intermediate range of distances, we observe an effective power-law decay $g(R) \sim R^{-\gamma}$, with γ of the same order as reported empirically, while the fitted exponent depends on the threshold τ and finite-size effects (Fig. 14). This demonstrates that Eq. 1 can generate scale-free spatial organization of tall structures compatible with the long-range correlation phenomenology reported in [3].

3. **Spanning city growth typologies (upward vs. outward).** Mahtta et al. [4] classify urban pixels in a four-dimensional descriptor (initial horizontal state, initial vertical state, and their changes ΔH and ΔV) and identify five generic growth modes: stabilized, outward, mature upward, budding outward, and upward & outward. This link this work with the approach developed in this paper, we explore how this typology classification relates with our model Eq. 1.

To quantify local vertical intensification versus horizontal broadening in a way that is robust to large dynamic ranges and minimizes cross-talk between the two, we work on a transformed intensity field $a_t(\mathbf{x}) = \log\big(1 + \max(h_t(\mathbf{x}), 0)\big)$ (at $t \in \{0, T\}$). The vertical component is defined pointwise as

$$\Delta V(\mathbf{x}) \;=\; a_T(\mathbf{x}) - a_0(\mathbf{x}), \tag{1}$$

so that positive values directly indicate local increases in height (or built mass), while the $\log(1 + \cdot)$ transform prevents a small number of extreme peaks from dominating the signal. For horizontal spreading, rather than thresholding (which can spuriously respond to uniform vertical scaling), we measure redistribution by normalizing intensity into a probability-like mass distribution

$$p_t(\mathbf{x}) \;=\; \frac{a_t(\mathbf{x})}{\sum_{\mathbf{y}} a_t(\mathbf{y}) + \varepsilon}, \tag{2}$$

and comparing local neighborhood shares of this mass. Let $\mathcal{N}_r[\cdot]$ denote the periodic $(2r+1)\times(2r+1)$ neighborhood mean (with wrap-around boundaries). We then define

$$\Delta H(\mathbf{x}) \;=\; \mathcal{N}_r[p_T](\mathbf{x}) - \mathcal{N}_r[p_0](\mathbf{x}). \tag{3}$$

This "mass-normalized neighborhood-share change" captures broadening/sprawl as a shift of mass from the core into surrounding neighborhoods (typically negative ΔH in the core and positive ΔH in the periphery), while being (approximately) invariant to purely vertical rescaling $a_T \approx c\, a_0$, for which $p_T = p_0$ and hence $\Delta H \approx 0$. Together, ΔV isolates local intensification and ΔH isolates spatial reallocation/broadening, enabling a clean bivariate mapping into upward, outward, and mixed growth regimes. While the local fields $\Delta H(\mathbf{x})$ and $\Delta V(\mathbf{x})$ resolve where broadening and intensification occur, for typology assignment it is often useful to summarize each simulation by a city-level trajectory in the same bivariate plane. We therefore define global, bounded scalars that preserve the interpretation of the local construction. Vertical change is taken as the relative increase in total transformed intensity $S_t = \sum_{\mathbf{x}} a_t(\mathbf{x})$, yielding $\Delta V_{\text{glob}}(t) = (S_t - S_0)/(S_t + S_0 + \varepsilon)$, which tracks overall intensification (built "volume") independently of spatial arrangement. Horizontal change is computed from the mass-normalized distribution $p_t(\mathbf{x}) = a_t(\mathbf{x})/(\sum_{\mathbf{y}} a_t(\mathbf{y}) + \varepsilon)$: after applying the neighborhood operator $\mathcal{N}_r$ to set the spatial scale, we summarize dispersion by an effective area $A_{\text{eff}}(t) = \big(\sum_{\mathbf{x}} \mathcal{N}_r[p_t](\mathbf{x})^2\big)^{-1}$, and define $\Delta H_{\text{glob}}(t) = (A_{\text{eff}}(t) - A_{\text{eff}}(0))/(A_{\text{eff}}(t) + A_{\text{eff}}(0) + \varepsilon)$. This yields a stable two-dimensional trajectory $(\Delta H_{\text{glob}}(t), \Delta V_{\text{glob}}(t))$ that cleanly distinguishes

outward (increasing dispersion), upward (increasing total intensity), and mixed growth over time, enabling direct comparison with typological classes and facilitating regime mapping as parameters (μ, k, σ) vary, see Fig. 16 and Fig. 15. This results shows that, Eq. 1 provides a minimal dynamical mapping onto this typology: the drift μ primarily controls vertical intensification, the coupling k promotes lateral propagation/smoothing (hence horizontal spread), and the multiplicative noise σ increases intermittency and patchiness. By varying (μ, k, σ) and the initial condition (nascent seed vs. already-developed core), simulations span regimes that correspond qualitatively to Mahtta et al.'s classes, including "mature upward" (large μ, limited spread), "outward" (strong spread with weak intensification), and mixed "upward & outward" behaviour when intensification and propagation are both strong.

4. **Heterogeneous parameters in space and time (toy zoning via $k(\mathbf{x}, t)$).** Eq. 1 generalizes straightforwardly to space- (and time-) dependent parameters, e.g.

$$\partial_t h(\mathbf{x}, t) = \mu(\mathbf{x}, t)\, h(\mathbf{x}, t) + \nabla \cdot \big(k(\mathbf{x}, t) \nabla h(\mathbf{x}, t)\big) + \sigma(\mathbf{x}, t)\, h(\mathbf{x}, t)\, \eta(\mathbf{x}, t).$$

   As an illustrative example, we consider a piecewise-constant $k(\mathbf{x})$ that partitions the domain into "districts" with distinct coupling strengths (interpretable as different zoning/market integration or infrastructure connectivity). Under identical (μ, σ), heterogeneous $k(\mathbf{x})$ produces visibly distinct skyline textures across districts (Fig. 17), showing how local policy-like heterogeneity can be represented without changing the core stochastic growth mechanism.

GBM compared to null model and the choice of the clock

In this appendix we compare a family of stochastic models for building height dynamics across cities using the Akaike Information Criterion [5]. For each city we analysed all available building trajectories and fit several continuous-time models under different choices of "clock":

- calendar time t,
- total population P,
- logarithm of population $\log P$.

Model classes. We consider the following stochastic specifications for height H:

- **GBM (time / population / log-population).** Geometric Brownian motion with drift and volatility defined with respect to the chosen clock X:

$$dH = \mu H \, dX + \sigma H \, dW,$$

  yielding Gaussian increments in log-height, $\Delta \log H \sim \mathcal{N}((\mu - \frac{1}{2}\sigma^2) \, \Delta X, \; \sigma^2 \Delta X)$.

- **ABM (time / population / log-population).** Arithmetic Brownian motion in levels:

$$dH = \mu \, dX + \sigma \, dW,$$

  so that $\Delta H \sim \mathcal{N}(\mu \, \Delta X, \; \sigma^2 \Delta X)$. This serves as a simple "null model" with linear drift and additive noise, without any multiplicative structure.

- **GBM with additive noise (time / population / log-population).** A hybrid model with GBM-like drift but additive noise:

$$dH = \mu H \, dX + \sigma \, dW,$$

  implying $\Delta H \sim \mathcal{N}(\mu H \, \Delta X, \; \sigma^2 \Delta X)$.

For each city and each model we construct increments across all buildings (using the appropriate clock $X \in \{t, P, \log P\}$), estimate (μ, σ) by maximum likelihood, and record the maximized log-likelihood $\ell_{\text{city, model}}$.

AIC and ΔAIC **per city.** The Akaike Information Criterion (AIC) for a given city and model is

$$\text{AIC}_{\text{city, model}} = 2k - 2 \, \ell_{\text{city, model}},$$

where k is the number of free parameters ($k = 2$ for all models considered here). For each city we identify the best-fitting model as the one with the smallest AIC, and define

$$\Delta\text{AIC}_{\text{city, model}} = \text{AIC}_{\text{city, model}} - \min_{\text{model'}} \text{AIC}_{\text{city, model'}}.$$

By construction, the best model in each city has $\Delta\text{AIC} = 0$, and larger ΔAIC indicate worse penalized fit relative to the city-specific optimum.

Results: time as best clock and GBM as best model. Figure 18 summarizes the distribution of ΔAIC across cities for all models and clocks. Two main conclusions emerge:

1. **Time is the best clock.** Models that use calendar time t as the underlying clock (GBM, ABM, and GBM with additive noise in time) systematically dominate those that use $\log P$ or P as clock. The time-based models are the AIC winners, and their ΔAIC distribution is concentrated near zero, while log-population and especially population clocks exhibit substantially larger ΔAIC, indicating poorer fit.

2. **GBM in time is the preferred stochastic specification.** Within the group of time-based models, GBM in time is the best model and has the lowest ΔAIC, suggesting that multiplicative, scale-proportional fluctuations in height provide a better description of the dynamics than purely additive noise. The ABM-in-time specification, interpreted as a simple null model with linear drift and constant additive variance, and the GBM with additive noise in time form the next-best group and serve as useful benchmarks to highlight the additional explanatory power of the GBM structure.

Taken together, these results support the use of calendar time as the natural clock for urban height dynamics, and they justify treating building heights as approximately following a geometric Brownian motion in time, rather than being driven primarily by population-based clocks or additive-noise-only models.

Longitudinal exponents are purely kinematic and differ from cross-sectional scaling exponents. Empirically, for each city c we observe that both mean height and population grow approximately exponentially in calendar time (Figure 19),

$$\bar{H}_c(t) \simeq H_{0,c}\, e^{\mu_c t}, \qquad N_c(t) \simeq N_{0,c}\, e^{\eta_c t},$$

with city-specific growth rates μ_c (for height) and η_c (for population). Eliminating time t along the trajectory of a single city then yields

$$\bar{H}_c(t) \propto N_c(t)^{\beta_c^{\mathrm{traj}}}, \qquad \beta_c^{\mathrm{traj}} = \frac{\mu_c}{\eta_c}.$$

The longitudinal exponent β_c^{traj} is thus a purely kinematic quantity: it is just the ratio of the city's height and population growth rates.

By contrast, the familiar cross-sectional scaling exponent β_{between} at a given date t_0 is defined as the slope of the regression of $\log \bar{H}_c(t_0)$ on $\log N_c(t_0)$ across cities,

$$\beta_{\text{between}}(t_0) = \frac{\mathrm{Cov}_c\big(\log N_c(t_0),\, \log \bar{H}_c(t_0)\big)}{\mathrm{Var}_c\big(\log N_c(t_0)\big)}.$$

Thus $\beta_{\text{between}}(t_0)$ is a property of the joint distribution of initial conditions and growth rates $(H_{0,c}, N_{0,c}, \mu_c, \eta_c)$ across cities at time t_0, and in general it is not equal to any particular city's ratio μ_c/η_c (nor to a simple average of these ratios). In other words, the longitudinal "trajectory" exponent β_c^{traj} and the cross-sectional scaling exponent β_{between} are fundamentally different objects: the former encodes the kinetics of individual city growth in calendar time, while the latter summarizes how cities of different size compare to each other at a given time slice. Figure 20 shows this β_{between} vs β_c^{traj} differences.

Supplementary Method

Stationary PDF of the spatially coupled GBM in the mean-field approximation

Stochastic mean-field model. We consider

$$\begin{aligned} \mathrm{d}h(t) &= \big[(\mu - k)h(t) + k\, m(t)\big]\,\mathrm{d}t + \sigma\, h(t)\,\mathrm{d}W_t, \\ m(t) &:= \mathbb{E}[h(t)], \quad h(0) = h_0. \end{aligned} \tag{4}$$

with deterministic drift μ, multiplicative noise amplitude $\sigma > 0$, and constant $k > 0$. Taking expectations in (4) yields

$$\dot{m}(t) = \mu\, m(t), \qquad m(t) = m_0\, \mathrm{e}^{\mu t}, \quad m_0 := m(0). \tag{5}$$

Centering transform and reduced SDE. Define the normalized process $u(t) := h(t)/m(t)$. Itô's lemma gives

$$\mathrm{d}u(t) = -k\big(u(t) - 1\big)\,\mathrm{d}t + \sigma\, u(t)\,\mathrm{d}W_t, \qquad u(0) = u_0 := h_0/m_0, \tag{6}$$

so that $h(t) = m(t)\,u(t)$ and, for any $x > 0$,

$$p_{h(t)}(x) = \frac{1}{m(t)}\, p_{u(t)}\left(\frac{x}{m(t)}\right). \tag{7}$$

A strong solution of (6) is

$$u(t) = \exp\left[(-k - \tfrac{1}{2}\sigma^2)t + \sigma W_t\right] \left(u_0 + k \int_0^t \exp\left[(k + \tfrac{1}{2}\sigma^2)s - \sigma W_s\right] \mathrm{d}s \right). \tag{8}$$

Thus the finite-time law of $u(t)$ is an affine functional of an exponential functional of Brownian motion, for which no elementary closed-form PDF is available in general. Below we provide exact moments and the stationary law.

Fokker–Planck equations and zero-flux stationarity. Let $p_h(x,t)$ denote the density of $h(t)$. The Fokker–Planck equation associated with (4) is

$$\begin{aligned} \partial_t p_h(x,t) &= -\partial_x\Big([(\mu - k)x + k\, m(t)]\, p_h(x,t) \Big) \\ &\quad + \frac{1}{2}\,\partial_{xx}\Big(\sigma^2 x^2\, p_h(x,t)\Big), \\ \text{with} \quad m(t) &= \int_0^\infty x\, p_h(x,t)\, \mathrm{d}x. \end{aligned} \tag{9}$$

For the normalized variable $u(t)$, the density $p(u,t)$ satisfies

$$\partial_t p(u,t) = -\partial_u\big(-k(u-1)\, p(u,t)\big) + \frac{1}{2}\,\partial_{uu}\big(\sigma^2 u^2\, p(u,t)\big). \tag{10}$$

Writing (10) as a continuity equation $\partial_t p + \partial_u J = 0$ defines the probability flux

$$\begin{aligned} J(u,t) &= a(u)\, p(u,t) - \tfrac{1}{2}\,\partial_u\big(b^2(u)\, p(u,t)\big), \\ a(u) &= -k(u-1), \quad b(u) = \sigma u. \end{aligned} \tag{11}$$

Derivation of the zero-flux stationary density. Setting $J(u) = 0$ in (11) yields

$$\begin{aligned} \partial_u\big(b^2(u)\, p(u)\big) &= 2a(u)\, p(u) \\ \Rightarrow \partial_u \ln\big(b^2(u)\, p(u)\big) &= \frac{2a(u)}{b^2(u)} \\ &= -\frac{2k}{\sigma^2}\left(\frac{1}{u} - \frac{1}{u^2}\right). \end{aligned} \tag{12}$$

Integrating gives

$$\begin{aligned} b^2(u)\, p(u) &= C\, u^{-2k/\sigma^2} \exp\left(-\frac{2k}{\sigma^2}\,\frac{1}{u}\right) \\ \Rightarrow \quad p_\infty(u) &= \frac{C}{\sigma^2}\, u^{-2-2k/\sigma^2} \exp\left(-\frac{2k}{\sigma^2}\,\frac{1}{u}\right). \end{aligned} \tag{13}$$

With $r := \sigma^2/(2k)$ and normalization, this becomes

$$p_\infty(u) = \frac{(1/r)^{\,1+1/r}}{\Gamma(1+1/r)}\, u^{-(2+1/r)} \exp\!\left(-\frac{1}{r\,u}\right), \qquad u > 0, \tag{14}$$

i.e. an inverse-gamma distribution with shape $1 + 1/r$ and scale $1/r$.

Moments and regime parameter. Let $\mathbb{E}[u_0] = 1$ (i.e. $\mathbb{E}[h_0] = m_0$). From Itô on u^2,

$$\begin{aligned} \frac{\mathrm{d}}{\mathrm{d}t}\,\mathbb{E}[u^2(t)] &= (-2k + \sigma^2)\,\mathbb{E}[u^2(t)] + 2k, \\ \Rightarrow \quad \mathbb{E}[u^2(t)] &= \mathrm{e}^{-2k(1-r)t}\left(\mathbb{E}[u_0^2] - \frac{1}{1-r}\right) + \frac{1}{1-r}, \end{aligned} \tag{15}$$

for $r \neq 1$ (and $\mathbb{E}[u^2(t)] = \mathbb{E}[u_0^2] + 2kt$ when $r = 1$). Hence, with $\mathbb{E}[u(t)] = 1$,

$$\mathrm{Var}[u(t)] = \begin{cases} \dfrac{r}{1-r}\left(1 - \mathrm{e}^{-2k(1-r)t}\right), & r \neq 1, \mathbb{E}[u_0^2] = 1, \\ 2kt, & r = 1,\ \mathbb{E}[u_0^2] = 1. \end{cases} \tag{16}$$

Regimes in terms of $r = \sigma^2/(2k)$.

- Subcritical noise ($r < 1$). The second moment converges: $\mathrm{Var}[u(t)] \to r/(1-r)$ as $t \to \infty$; the normalized process $u(t)$ converges in distribution to (14), which has finite variance. Consequently, $h(t) = m(t)\,u(t)$ has a "shape-stable" distribution whose scale grows like $m(t) = m_0\,\mathrm{e}^{\mu t}$.

- Critical noise ($r = 1$). The variance grows linearly, $\mathrm{Var}[u(t)] = 2kt$, and the invariant density (14) exists but has infinite variance (shape parameter $= 2$).

- Supercritical noise ($r > 1$). The second moment explodes exponentially,

$$\mathrm{Var}[u(t)] = \frac{r}{r-1}\left(\mathrm{e}^{\,2k(r-1)t} - 1\right), \tag{17}$$

  and the invariant density (14) has infinite variance (but remains normalizable). In this regime, tails are heavy enough that finite-time moments of order ≥ 2 eventually become dominated by rare events.

Inter-satellite bias correction

We convert C-/Ku-band backscatter σ° (dB) to the unitless power-return ratio h on the native 0.05° grid, as reported in [6], using only May composites to minimize seasonality:

$$h_{c,i,s,t}^{\text{May}} \;=\; 10^{\,\sigma^\circ_{c,i,s,t,\text{May}}/10},$$

where c indexes cities, i grid cells, s satellites, and t calendar years (1993–2020). Time is referenced to the city-specific first observation, $t \leftarrow \text{year} - t_0^{(c)}$, with $t_0^{(c)} = \min\{\text{year in city } c\}$. We analyze log-intensity $x_{i,s,t}^{(c)} = \log h_{i,s,t}^{(c)}$. The three satellites cover partially overlapping periods:

$$\text{ERS: 1993–2000}, \qquad \text{QSCAT: 1999–2009}, \qquad \text{ASCAT: 2007–2021},$$

yielding overlap windows ERS $\leftrightarrow$ QSCAT : 1999–2000 and QSCAT $\leftrightarrow$ ASCAT : 2007–2009. We take QSCAT as the reference, denoted $s^\star = \text{QSCAT}$.

Per-pixel overlap intercalibration (ASCAT↔QSCAT). For each city c and pixel i observed by ASCAT and QSCAT in the overlap years $\mathcal{T}_{i,\text{ASCAT}\leftrightarrow s^\star} = \{2007, 2008, 2009\}$, we use a pixel-specific affine relation on the log scale

$$x_{i,\text{ASCAT},t}^{(c)} \;=\; a_{i,\text{ASCAT}}^{(c)} \;+\; b_{i,\text{ASCAT}}^{(c)}\, x_{i,s^\star,t}^{(c)} \;+\; u_{i,t}^{(c)}, \qquad t \in \mathcal{T}_{i,\text{ASCAT}\leftrightarrow s^\star}.$$

Here, $a_{i,\text{ASCAT}}^{(c)}$ (intercept) and $b_{i,\text{ASCAT}}^{(c)}$ (slope) are unknown pixel-specific parameters, and $u_{i,t}^{(c)}$ is the residual error. We estimate $a_{i,\text{ASCAT}}^{(c)}$ and $b_{i,\text{ASCAT}}^{(c)}$ by ordinary least squares on the overlap years, and denote the estimates by $\widehat{a}_{i,\text{ASCAT}}^{(c)}$ and $\widehat{b}_{i,\text{ASCAT}}^{(c)}$.

Per-pixel intercept alignment (ERS↔QSCAT). Because the ERS–QSCAT overlap (1999–2000) is short, we align ERS to QSCAT at the pixel level using an intercept-only shift:

$$x_{i,\text{ERS},t}^{(c)} \;=\; a_{i,\text{ERS}}^{(c)} \;+\; x_{i,s^\star,t}^{(c)} \;+\; v_{i,t}^{(c)}, \qquad t \in \{1999, 2000\}.$$

Here, $a_{i,\text{ERS}}^{(c)}$ is an unknown pixel-specific intercept (level offset) and $v_{i,t}^{(c)}$ is the residual error. We estimate $a_{i,\text{ERS}}^{(c)}$ on the available overlap year(s); with one year it reduces to the log-difference $x_{i,\text{ERS},t}^{(c)} - x_{i,s^\star,t}^{(c)}$, and with two years it is the average of those differences. The estimate is denoted by $\widehat{a}_{i,\text{ERS}}^{(c)}$.

Applying per-pixel calibration. The estimated per-pixel mappings are applied to all years to place each satellite on the QSCAT scale:

$$\log \tilde{h}^{(c)}_{i,s,t} = \begin{cases} x^{(c)}_{i,s^\star,t}, & s = s^\star \ \text{(QSCAT)}, \\ \left(x^{(c)}_{i,\text{ASCAT},t} - \widehat{a}^{(c)}_{i,\text{ASCAT}}\right) \big/ \widehat{b}^{(c)}_{i,\text{ASCAT}}, & s = \text{ASCAT}, \\ x^{(c)}_{i,\text{ERS},t} - \widehat{a}^{(c)}_{i,\text{ERS}}, & s = \text{ERS}. \end{cases} \quad (18)$$

Validation of the inter-calibration We provide several figures to illustrate and validate the intercalibration of the multi-satellite backscatter record from [6] (that we refers as the radar data).

Figure 8 contrasts raw and calibrated time series for Guangzhou. Before calibration, satellite segments occupy distinct level regimes with short overlap windows. For each satellite s, panel (a) reports the within-window γ_s and the average over segments $\overline{\gamma}_{\text{uncalib}} = (\gamma_{\text{ers}} + \gamma_{\text{quikscat}} + \gamma_{\text{ascat}})/3$ as a rough 1993–2020 approximation; this closely matches the calibrated city-wide value γ_{calib} (for 1993-2020 window) obtained after intercalibration in panel (b). Panel (c) compares $\overline{\gamma}_{\text{uncalib}}$ to γ_{calib} across all cities, showing that intercalibration has little effect on γ and mainly harmonizes levels across satellites while preserving growth rates.

Figure 9 illustrate the coarsening and dataset comparability with [7] that we use for comparison of the radar data. We plotted the 30 m building height for Beijing, the same product aggregated to our analysis grid, and the radar power-return at that grid. The coarsening step preserves the large-scale spatial patterns, and the radar field at the coarser resolution exhibits correspondence with the aggregated building heights.

Figure 10 evaluates γ robustness. Panel (a) compares γ estimated on 1993–2020 against γ estimated on 2001–2020 (both after intercalibration). The close agreement indicates that excluding the ERS segment—which has a very short overlap with QuikSCAT—does not alter growth-rate inference. Panel (b) compares γ from 2005–2020 (radar, calibrated) against γ computed from the 30 m dataset [7] coarsened to 0.05° (see Figure 9). Despite differing data sources and temporal sampling (2005, 2010, 2015, 2020 for the 30 m product), the city-level growth ranges are similar, with good concordance for larger cities (area $> 1250\,\text{km}^2$, approximately > 50 grid cells). Panel (c) shows the same comparison using radar γ from 1993–2020, yielding consistent conclusions.

Together, these indicate that the intercalibration produces stable, cross-satellite city trajectories and that the inferred growth rates are consistent with an independent, high-resolution benchmark.

Distance to a Theoretical Stationary Law via Jensen–Shannon Divergence

Let $h_{c,y,i}$ denote the height for observation i in city c and year y. To isolate distributional shape from level changes, we normalize heights within each city–year:

$$u_{c,y,i} \;=\; \frac{h_{c,y,i}}{\bar{h}_{c,y,\cdot}}.$$

Under the stationary hypothesis (Eq.14), the normalized random variable U follows an inverse–gamma law that depends on a single parameter $r > 0$,

$$p_\infty(u \mid r) = \frac{(1/r)^{\,1+1/r}}{\Gamma\!\left(1+\frac{1}{r}\right)}\, u^{-\left(2+\frac{1}{r}\right)}\, \exp\!\left(-\frac{1}{r\,u}\right), \qquad u > 0, \tag{19}$$

i.e., $U \sim \mathrm{InvGamma}(\alpha, \beta)$ with $\alpha = 1 + 1/r$ and $\beta = 1/r$.

Estimating the stationary reference. For each city c, we estimate $\hat{r}_c$ from the last available year $y_{\max}(c) = 2020$ by maximum likelihood, equivalently fitting $U \sim \mathrm{InvGamma}(\alpha_c, \beta_c)$ with the reparametrization $\alpha_c = 1+1/\hat{r}_c$ and $\beta_c = 1/\hat{r}_c$. This yields a time–invariant theoretical stationary density $p_\infty(\cdot \mid \hat{r}_c)$ used as reference for that city.

Empirical year-specific distributions. For each city–year (c, y) we form an empirical distribution of U on a shared grid of bins (per city): the bin range is set to the pooled 1–99th percentiles of $\{u_{c,y,i}\}_y$, and the number of bins is chosen by the Freedman–Diaconis rule [8]. Let $\mathbf{P}_{c,y} = \{p_{c,y,k}\}_k$ be the empirical bin probabilities (histogram counts normalized so that $\sum_k p_{c,y,k} = 1$). Let $\mathbf{Q}_c = \{q_{c,k}\}_k$ be the corresponding bin probabilities obtained by integrating the fitted density, $q_{c,k} = \int_{\mathrm{bin}\ k} p_\infty(u \mid \hat{r}_c)\,\mathrm{d}u$ (computed from the inverse–gamma CDF).

Jensen–Shannon divergence (JSD). We quantify the dissimilarity between the empirical year-y distribution and the stationary reference using the base-2 Jensen–Shannon divergence [9]:

$$\mathrm{JSD}_2(\mathbf{P}_{c,y} \,\|\, \mathbf{Q}_c) \;=\; \tfrac{1}{2}\,\mathrm{KL}_2\!\big(\mathbf{P}_{c,y} \,\|\, \mathbf{M}_{c,y}\big) \;+\; \tfrac{1}{2}\,\mathrm{KL}_2\!\big(\mathbf{Q}_c \,\|\, \mathbf{M}_{c,y}\big), \quad \mathbf{M}_{c,y} = \tfrac{1}{2}(\mathbf{P}_{c,y} + \mathbf{Q}_c),$$

where KL_2 uses $\log_2$. Thus $\mathrm{JSD}_2 \in [0, 1]$, with 0 indicating identical distributions.

Trend and short-horizon diagnostics. For each city we obtain a time series $d_{c,y} = \mathrm{JSD}_2(\mathbf{P}_{c,y} \| \mathbf{Q}_c)$ over years. To summarize convergence toward the stationary law, we estimate a linear trend s_c (slope per year) of $d_{c,y}$ vs. y ; a negative slope indicates decreasing divergence (convergence).

Local-width roughness on a log–height field with masking and pre-plateau fitting

Log transform and validity mask. Starting from a height field $h(x, y)$, we analyze the log–height field

$$\phi(x,y) \;=\; \log\bigl(h(x,y)+\varepsilon\bigr) \;-\; \min_{(i,j)\in\mathcal{V}} \log\bigl(h_{ij}+\varepsilon\bigr) \;+\; \delta,$$

with a small offset $\varepsilon > 0$ (chosen a priori; $\varepsilon = 0.01\,\mathrm{median}\{h > 0\}$) and a tiny shift $\delta > 0$ ($\delta = 10^{-6}$) to make $\phi > 0$ on valid pixels. Pixels where $h + \varepsilon \le 0$ are treated as holes (set to 0 in memory but excluded by the mask). The set of valid pixels is

$$\mathcal{V} = \{(x, y) : \; h(x, y) + \varepsilon > 0, \;\, h \text{ finite}\}.$$

All statistics below are computed exclusively over $\mathcal{V}$. Because the local mean is subtracted inside each box (see below), the global constant shift $-\min\log(h+\varepsilon)+\delta$ does not affect the local-width values.

Local-width. For a square box of side ℓ centered at (x, y), let $B_\ell(x, y) \subset \mathcal{V}$ be the set of valid pixels in the box. The local width on ϕ is the RMS after subtracting the local mean:

$$w_\ell^2(x,y) = \frac{1}{|B_\ell(x,y)|} \sum_{(i,j)\in B_\ell(x,y)} \Bigl(\phi_{ij} - \bar\phi_\ell(x,y)\Bigr)^2,$$

$$\bar\phi_\ell(x,y) = \frac{1}{|B_\ell(x,y)|} \sum_{(i,j)\in B_\ell(x,y)} \phi_{ij}.$$

Boxes are accepted only if their coverage exceeds a threshold,

$$\frac{|B_\ell(x,y)|}{\ell^2} \;\ge\; \texttt{min\_coverage} \;\; \text{(set to 0.8)},$$

otherwise they are discarded. The scale-aggregated width is the across-box average

$$w(\ell) = \bigl\langle w_\ell(x,y) \bigr\rangle_{\text{boxes at size } \ell}.$$

We estimate $w(\ell)$ on a geometric ladder $\ell \in \{\ell_0,\ \ell_0 r,\ \ell_0 r^2, \dots\}$ with ratio $r \approx 1.25$, from `min_box` up to `max_box` (default $\simeq L/4$, where L is the smaller image side). For each ℓ we also record the sample standard deviation across boxes, $s_w(\ell)$, and the number of contributing boxes, $n(\ell)$.

Pre-plateau extraction of the roughness exponent α (see Fig.11 for an illustration). For a self-affine field, the local width scales as

$$w(\ell)\ \sim\ \ell^{\alpha} \qquad (a \ll \ell \ll L),$$

where a is the pixel size and L the system size. At large ℓ finite-size effects induce a plateau. We therefore fit only the pre-plateau band via:

1. **Log–log variables:** $x_i = \log \ell_i$, $y_i = \log w(\ell_i)$ for scales with $w(\ell_i) > 0$ and $n(\ell_i) > 0$.
2. **Plateau detection by local slopes:** compute a sliding OLS slope s_i of y vs. x over w points (set $w = 5$). The plateau onset index i^* is the first position where two consecutive slopes satisfy $s_i < s_{\text{flat}}$ and $s_{i+1} < s_{\text{flat}}$ (default $s_{\text{flat}} = 0.3$). The fit band is $[i_0, i_1]$ with i_0 skipping the smallest scales (in this study we used `ignore_first`$= 0$ i.e. we do not skip the first box) and $i_1 = \max(i^* - 1,\ i_0 + \texttt{min\_points} - 1)$. If no plateau is detected, fit up to the largest scale; require at least `min_points` (set to 6).
3. **Weighted least squares:** on $[i_0, i_1]$, estimate

   $$y = \alpha\, x + b + \varepsilon$$

   by WLS with weights

   $$w_i\ \propto\ \frac{n(\ell_i)}{[s_w(\ell_i)/w(\ell_i)]^2},$$

   i.e., inverse-variance weighting in $\log w$ scaled by the number of boxes. Report $\hat{\alpha}$ and its standard error from the WLS covariance.

Supplementary Figures

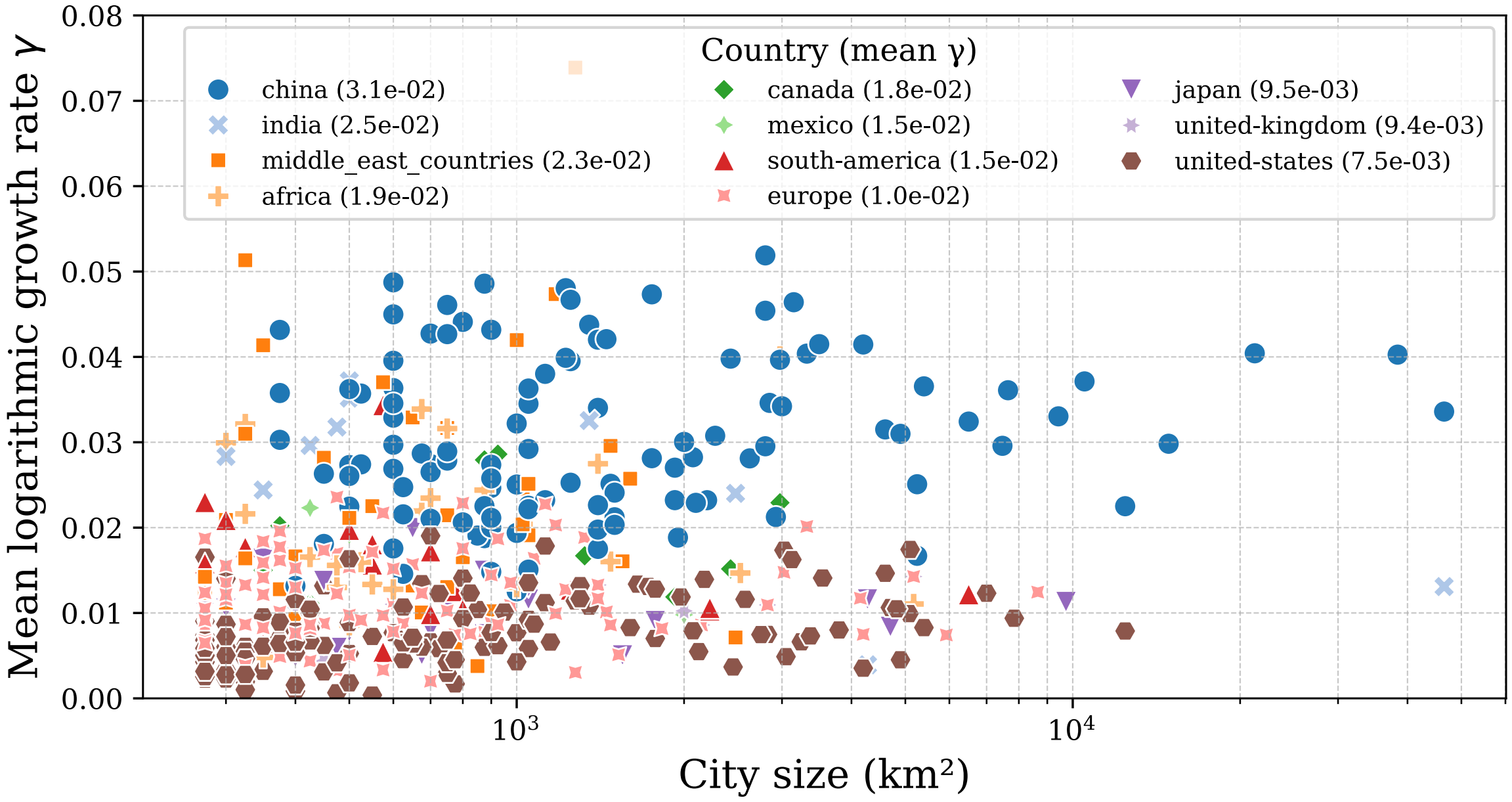

Supplementary Figure 1: Mean logarithmic growth rate γ (Eq. 4) as a function of city size across all cities in the sample; points are colored by country/region.

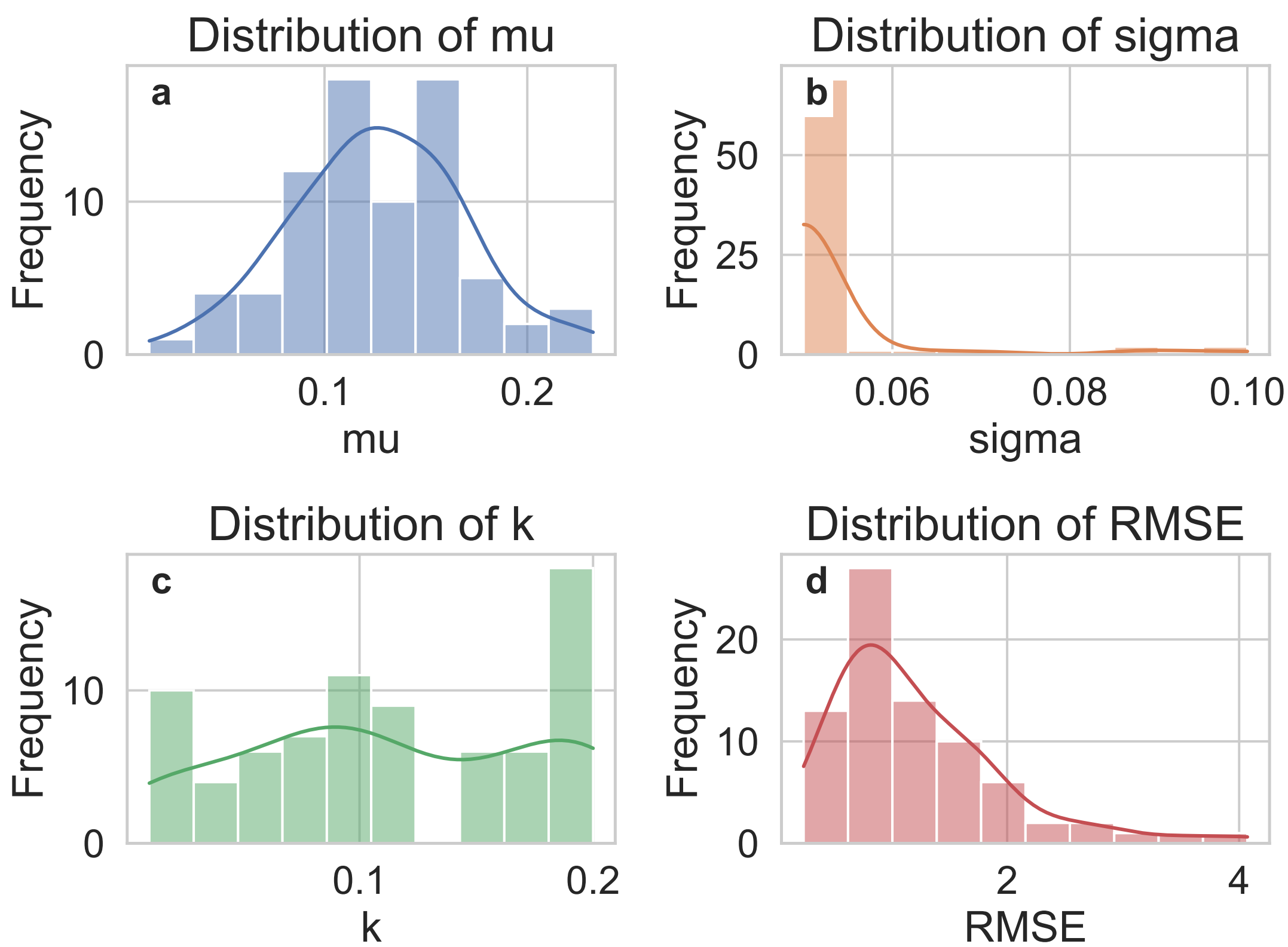

Supplementary Figure 2: Distributions of the calibrated parameters and simulation error across Chinese cities: (a) growth rate μ (b) volatility σ(c) interaction strength k, and (d) RMSE between simulated and observed trajectories.

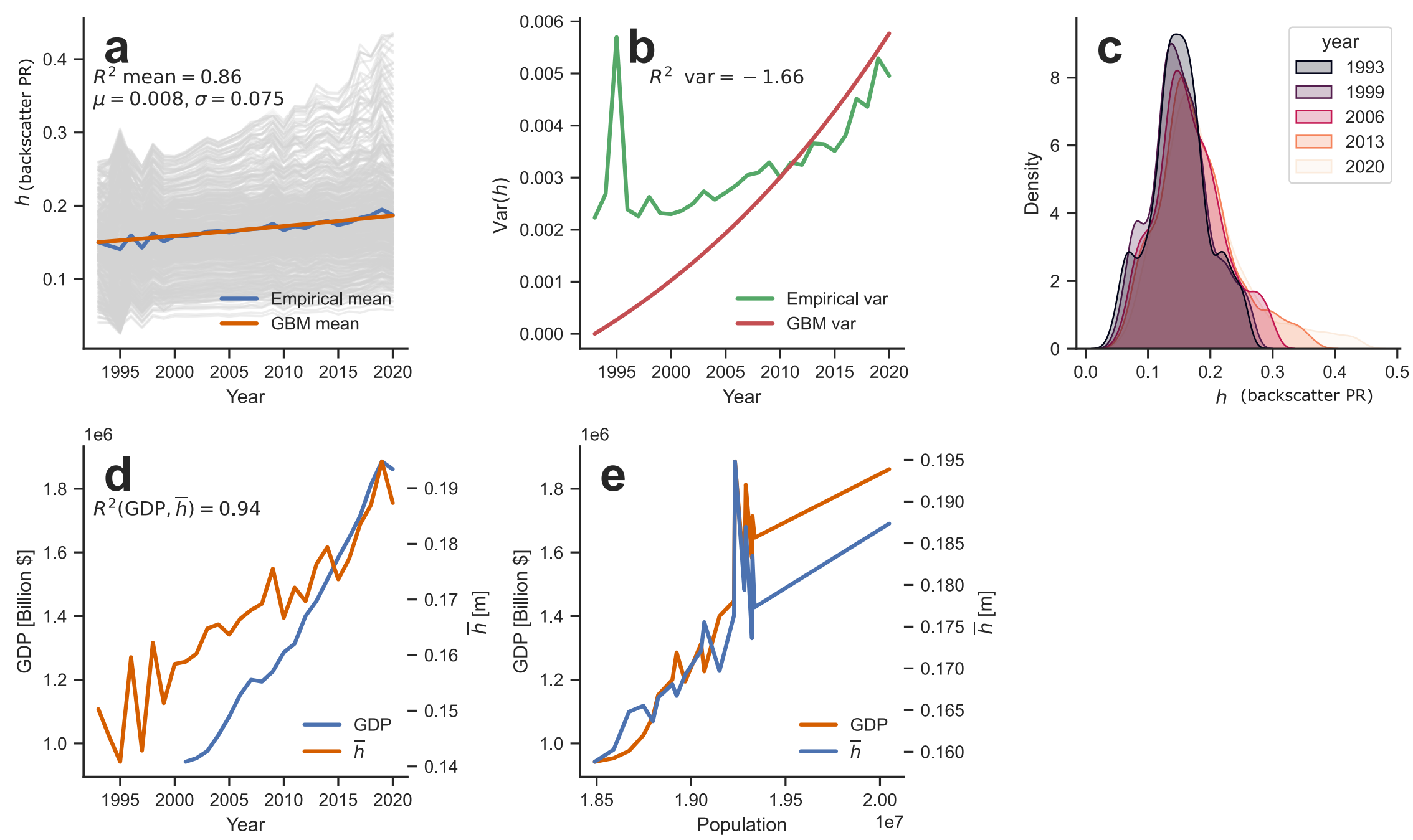

Supplementary Figure 3: New-York city: Comparison of empirical building–height statistics with the geometric Brownian-motion (GBM) model for each city. **(a)** Mean of pixel height $h(t)$; **(b)** height variance $\mathrm{Var}(h)$; **(c)** kernel-density estimates of height for five benchmark years; **(d)** co-evolution of GDP and mean height; **(e)** GDP versus population with height overlay. Shaded grey lines show pixel buildings height, coloured lines show city means (blue) and GBM predictions (orange/red). In-panel boxes list fitted GBM parameters and the corresponding coefficients of determination R^2.

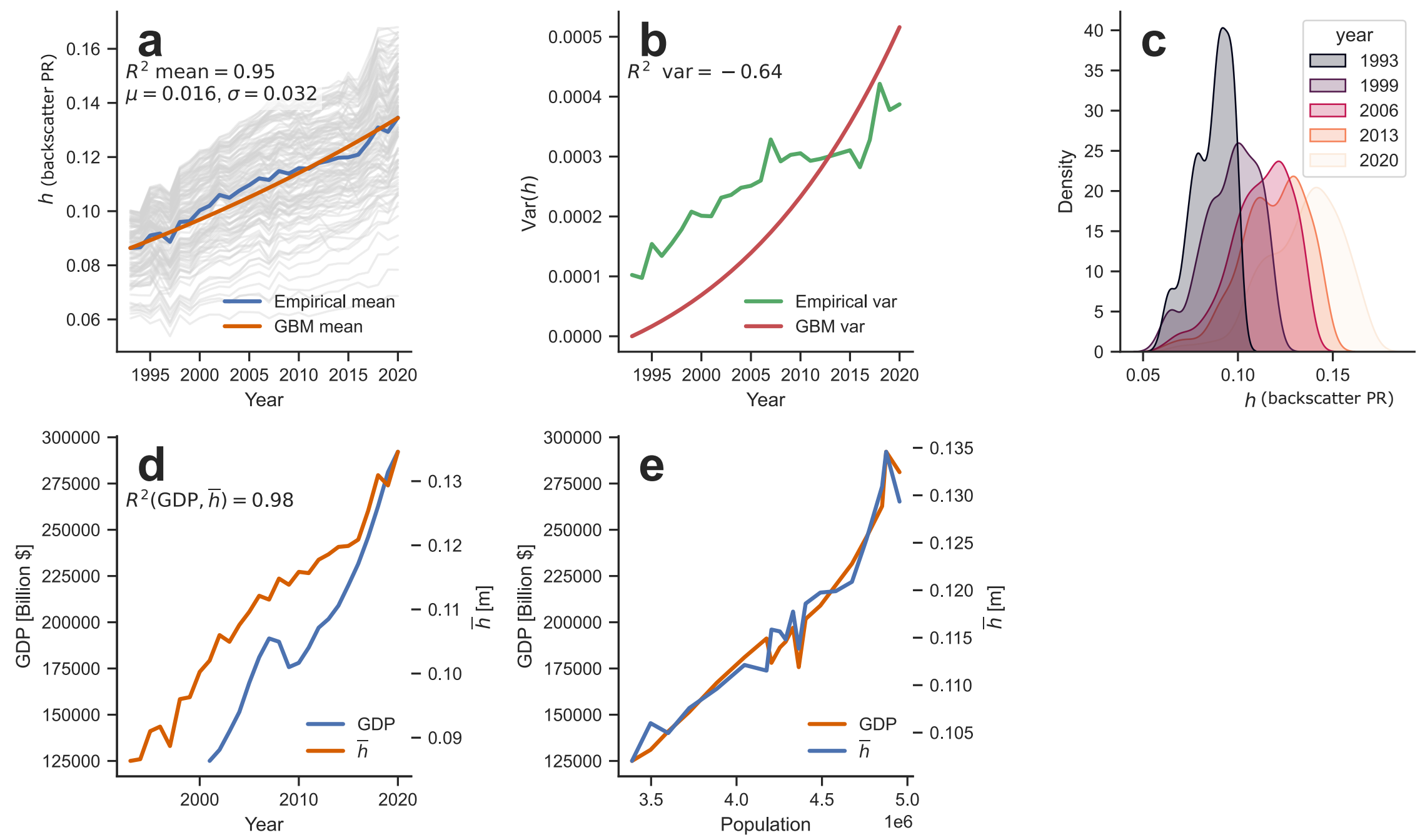

Supplementary Figure 4: Phoenix: Comparison of empirical building–height statistics with the geometric Brownian-motion (GBM) model for each city. **(a)** Mean height $h(t)$; **(b)** height variance $\mathrm{Var}(h)$; **(c)** kernel-density estimates of height for five benchmark years; **(d)** co-evolution of GDP and mean height; **(e)** GDP versus population with height overlay. Shaded grey lines show pixel buildings height, coloured lines show city means (blue) and GBM predictions (orange/red). In-panel boxes list fitted GBM parameters and the corresponding coefficients of determination R^2.

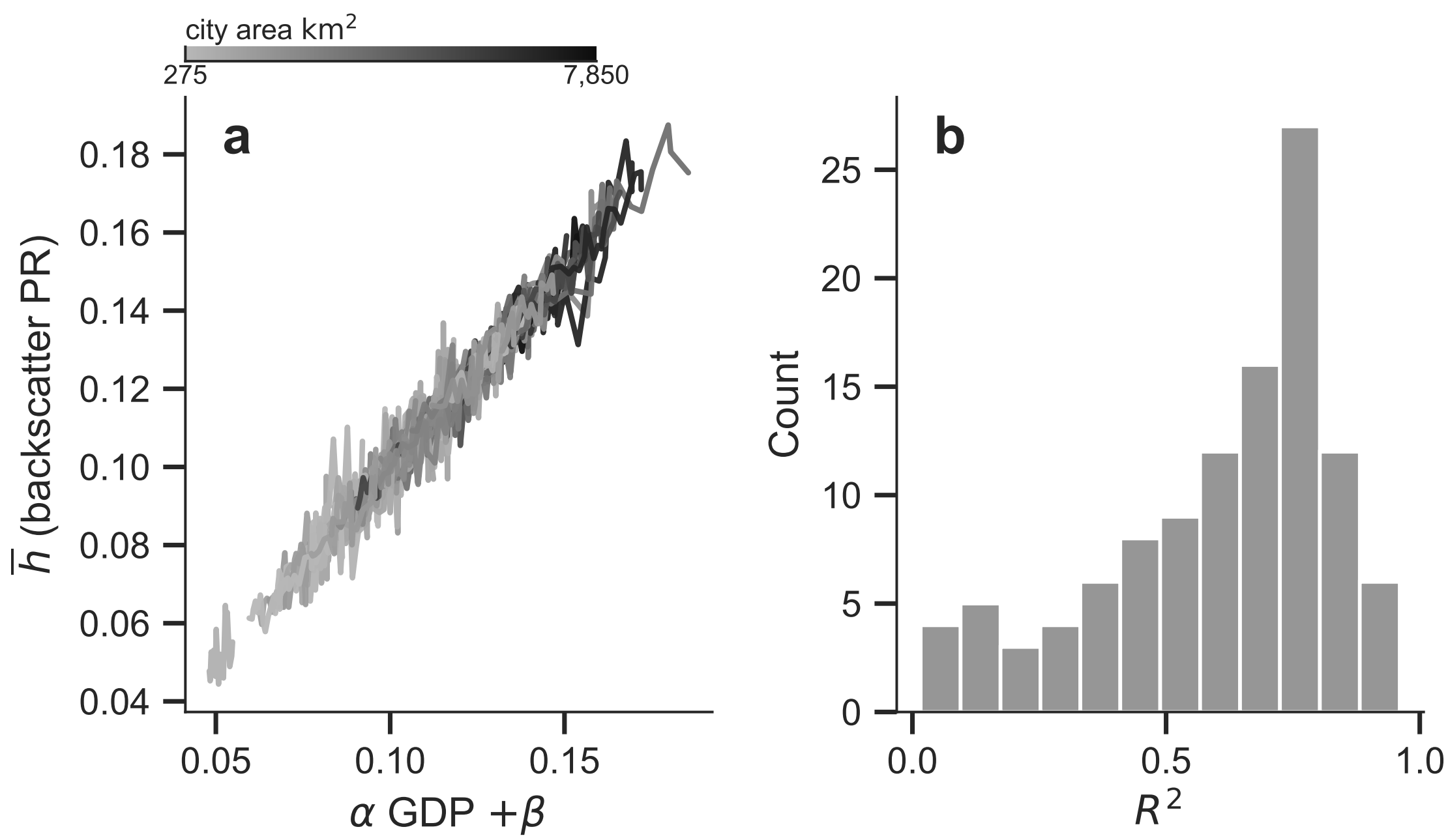

Supplementary Figure 5: **(a)** Mean USA city height express as linear linear combination of city GDP.**(b)** Coefficient of determination per city.

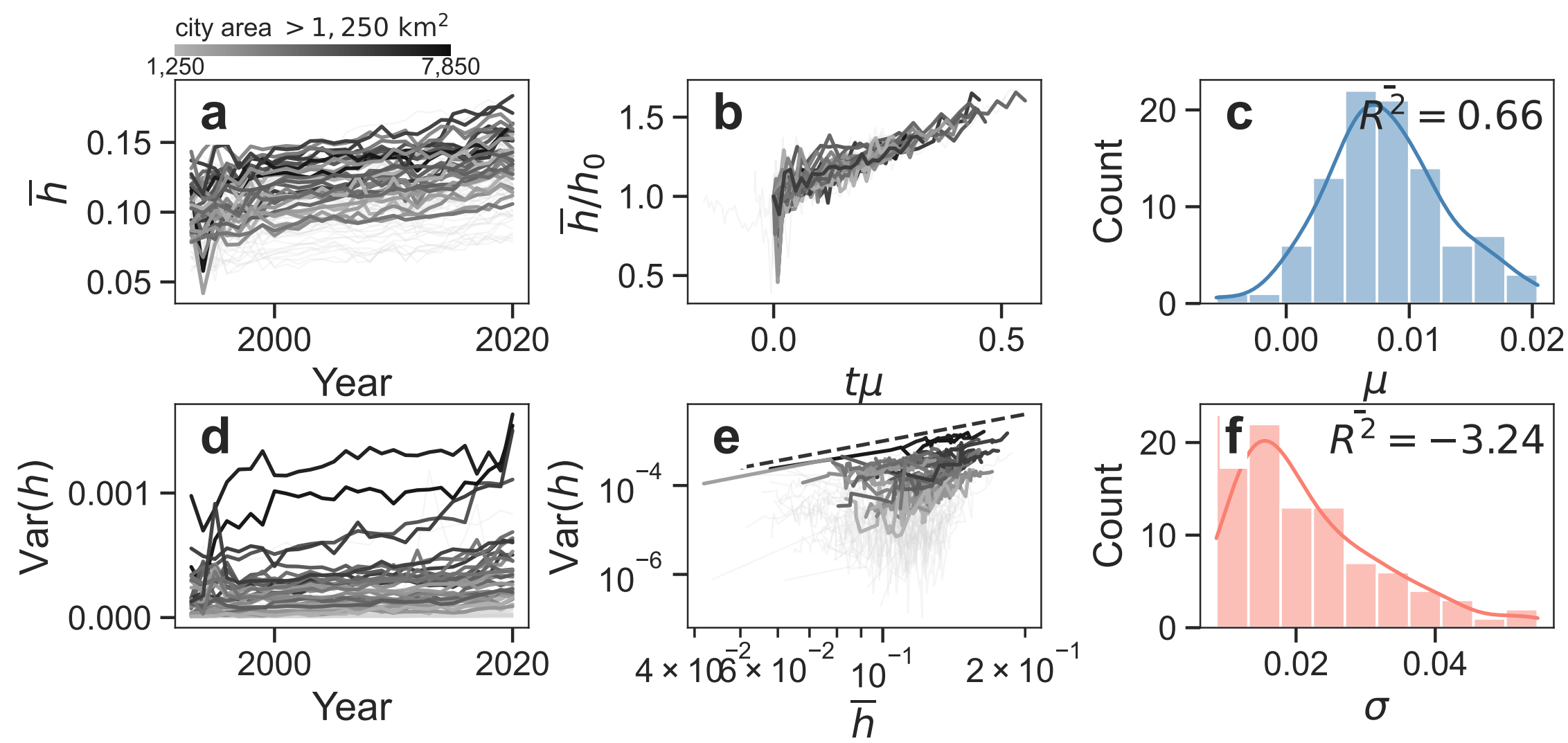

Supplementary Figure 6: Largest USA Cities: (a) Empirical mean building height $\overline{h}(t)$ for each city. (b) Same trajectories rescaled by their drift term ($t\mu$). (c) Distribution of estimated drift coefficients. (d) Empirical variance of building heights over time. (e) Relationship between variance and mean height. (f) Distribution of estimated volatility values.

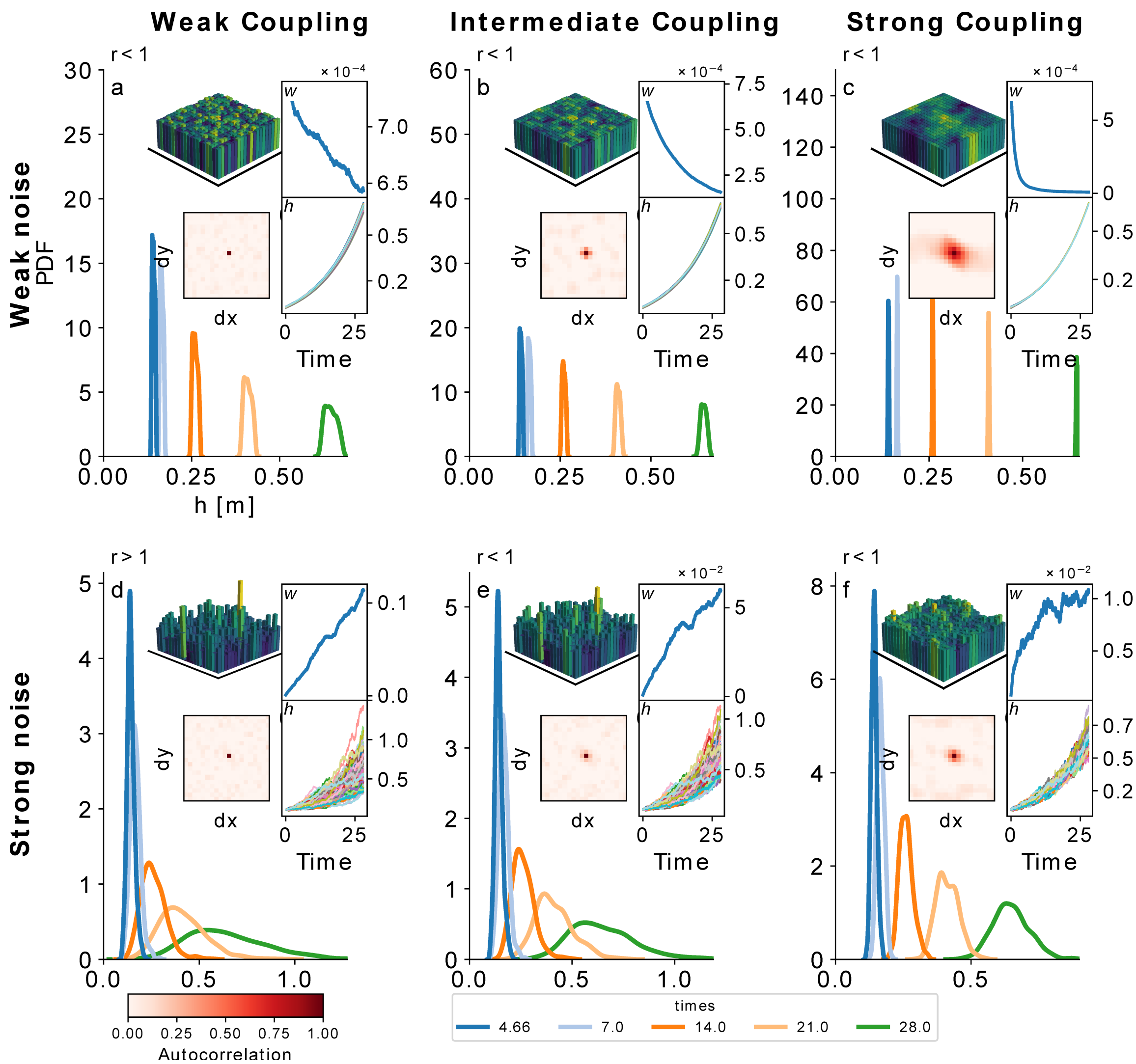

Supplementary Figure 7: Synthetic height fields generated by the stochastic growth model (Eq.1) for six parameter regimes (weak noise (a-c), strong noise (d-f); Main panels: kernel–density estimates of the height probability density at four times. Insets (per panel, top→bottom/left→right) show: final 3-D height surface, 20×20-pixel autocorrelation of the same surface, time series of the relative variance $w(t) = \mathrm{Var}(h)/\overline{h}^2$, and trajectories of 100 randomly sampled pixels $h(t)$.

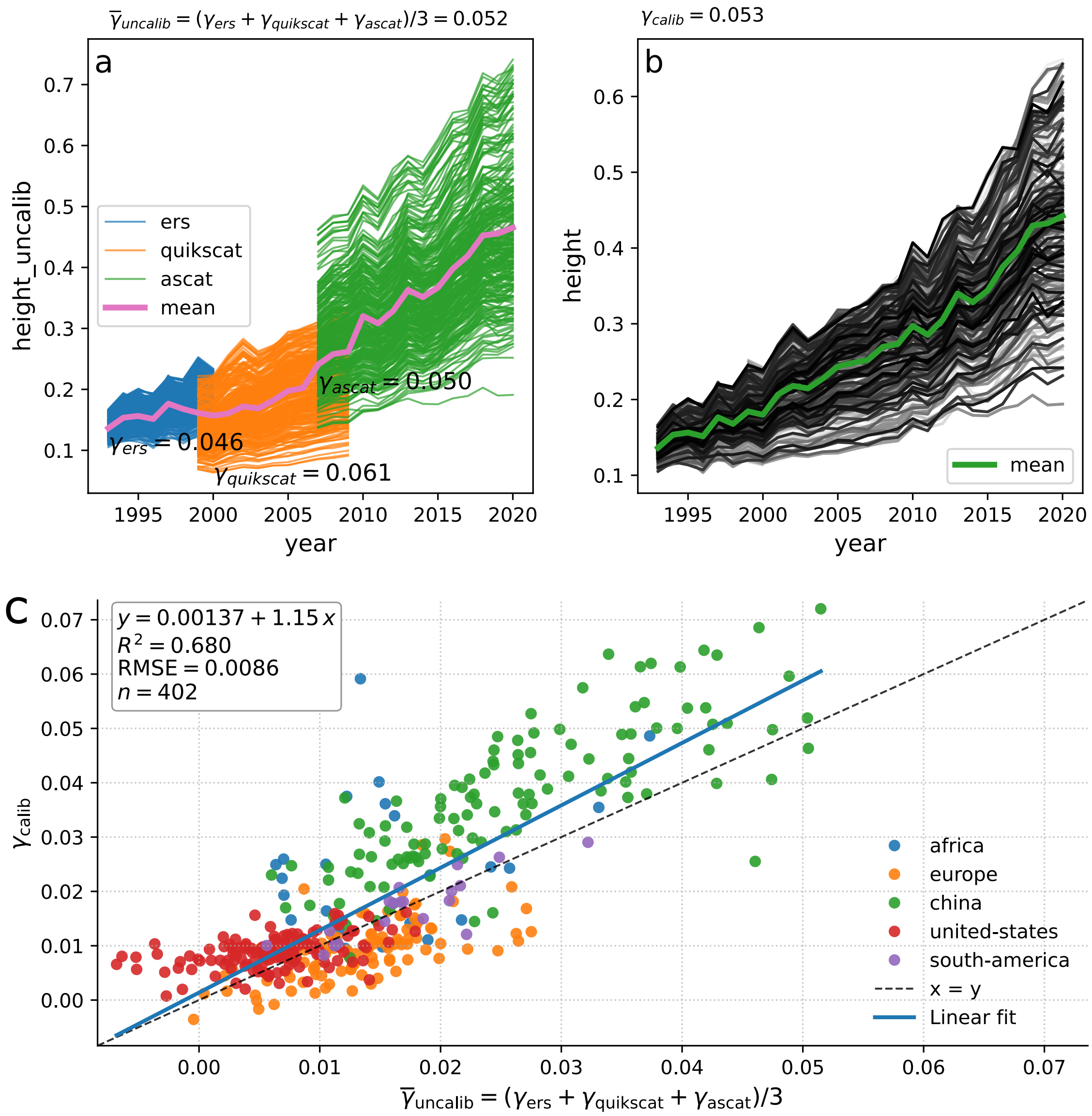

Supplementary Figure 8: Guangzhou intercalibration illustation. **(a)** Backscatter power-return ratio versus time before intercalibration. For each satellite segment we report the within-window γ_{calib} and the average of the three segment $\overline{\gamma}_{\text{uncalib}}$ as an approximation 1993-2020. **(b)** Pixel time series after intercalibration. The harmonized series forms a continuous trajectory and supports a stable city-wide slope over 1993–2020. **(c)** Global comparison of $\overline{\gamma}_{\text{uncalib}}$ versus γ_{calib} across cities, indicating that intercalibration has minimal impact on γ while aligning satellite levels.

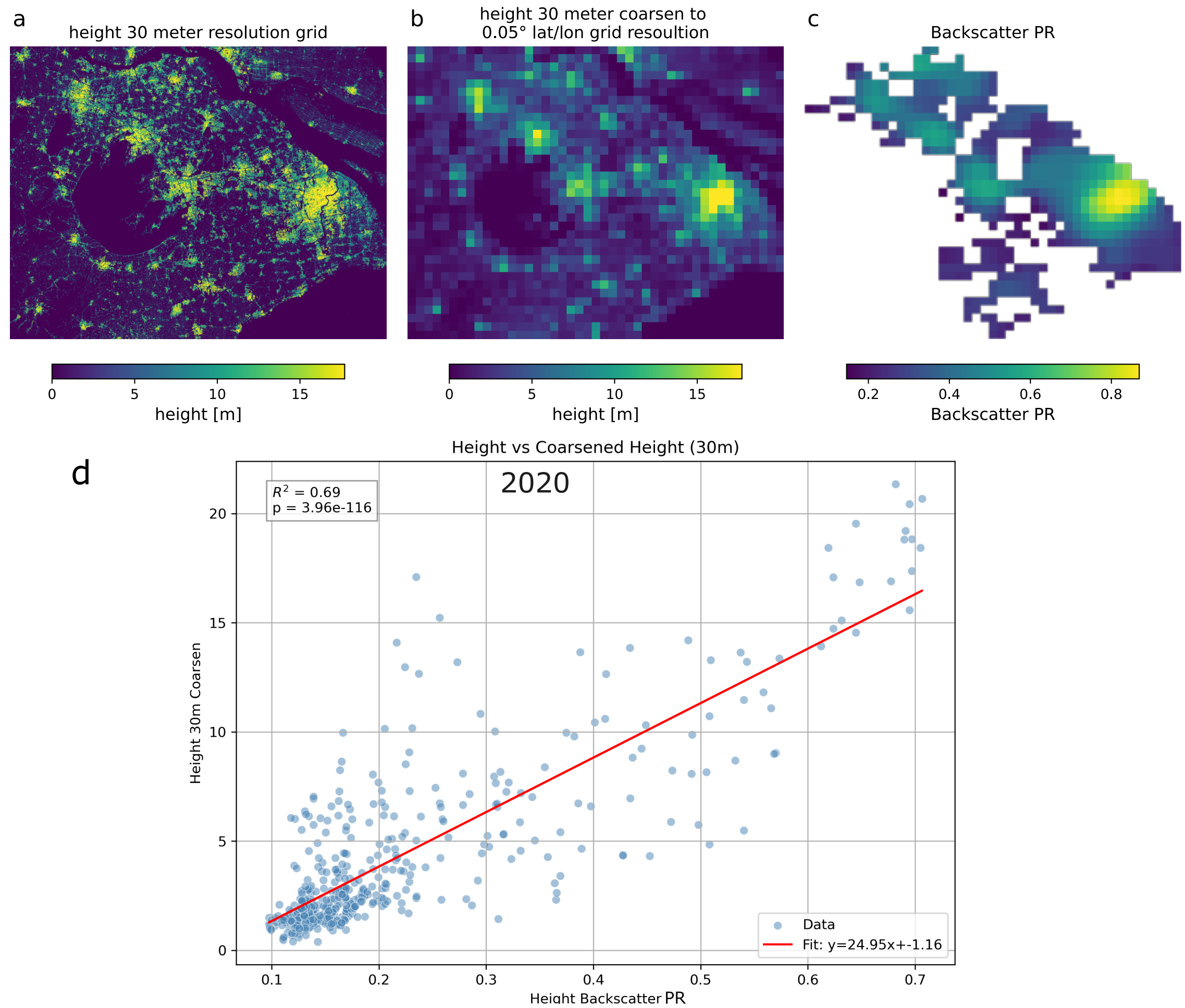

Supplementary Figure 9: Coarsening and cross-dataset comparison (Beijing) for year 2020. **(a)** Building height at 30 m resolution. **(b)** The same product aggregated to the 0.05° grid by averaging 30 m pixels within each cell (benchmark year 2020). **(c)** Radar backscatter power-return ratio (PR) at the 0.05° grid. The coarsening preserves large-scale spatial structure, and the radar field at analysis resolution shows plausible correspondence with aggregated building height. **(d)** PR–height in meters calibration for Beijing. Points are 0.05° grid cells within the MUA; PR is regressed against coarsened CMTBH-30 height.

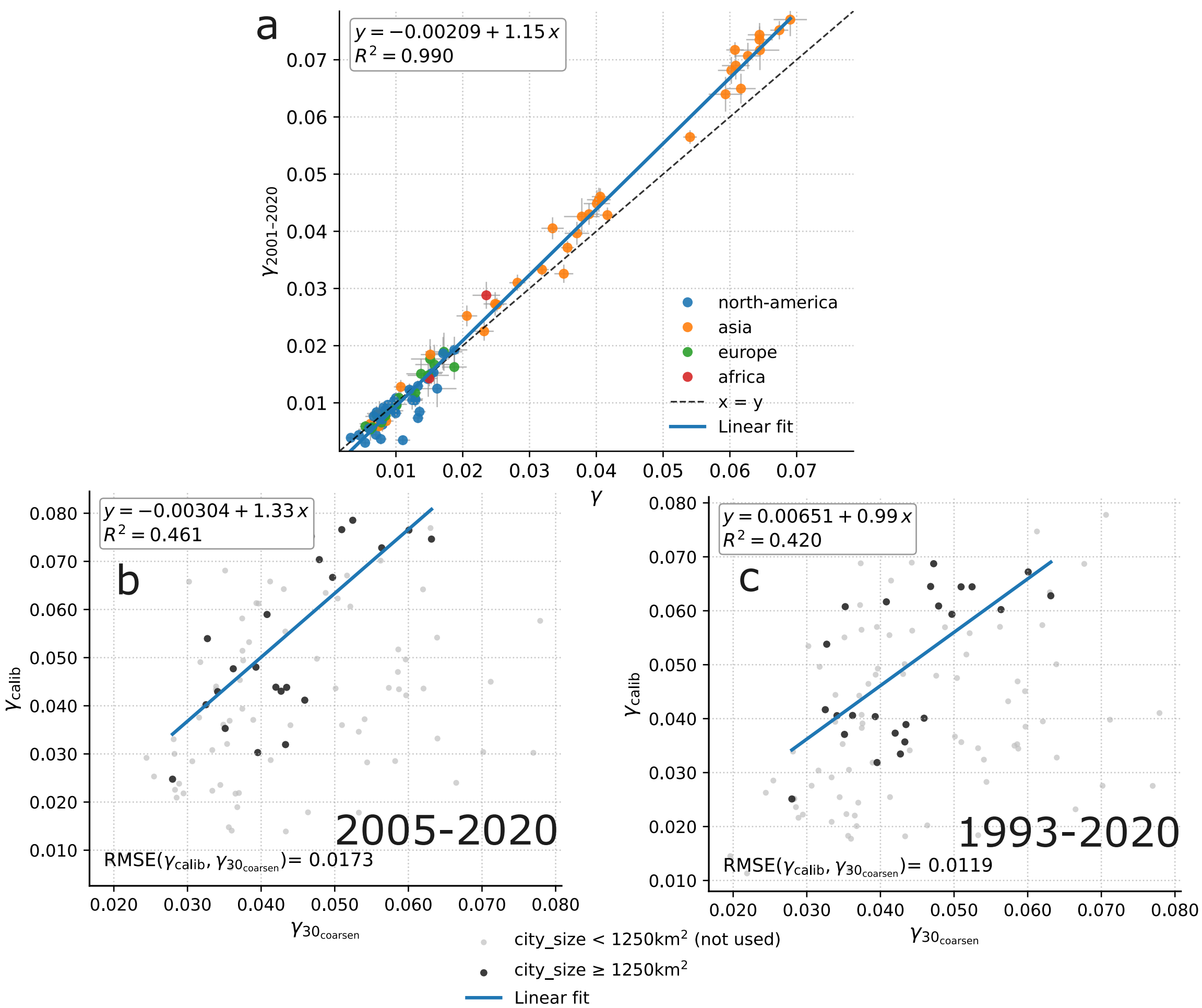

Supplementary Figure 10: Validation of city growth rates γ. **(a)** Comparison of γ from 1993–2020 (after intercalibration) versus 2001–2020 (after intercalibration), demonstrating robustness to excluding the ERS segment with short overlap. **(b)** Comparison of radar-based γ from 2005–2020 (after intercalibration) with γ computed from the 30 m dataset [7] coarsened to 0.05° for Chinese cities; the ranges agree well for cities larger than 1250 km^2 (approximately more than 50 grid cells). **(c)** Same as panel (b) but using radar γ from 1993–2020, yielding consistent conclusions.

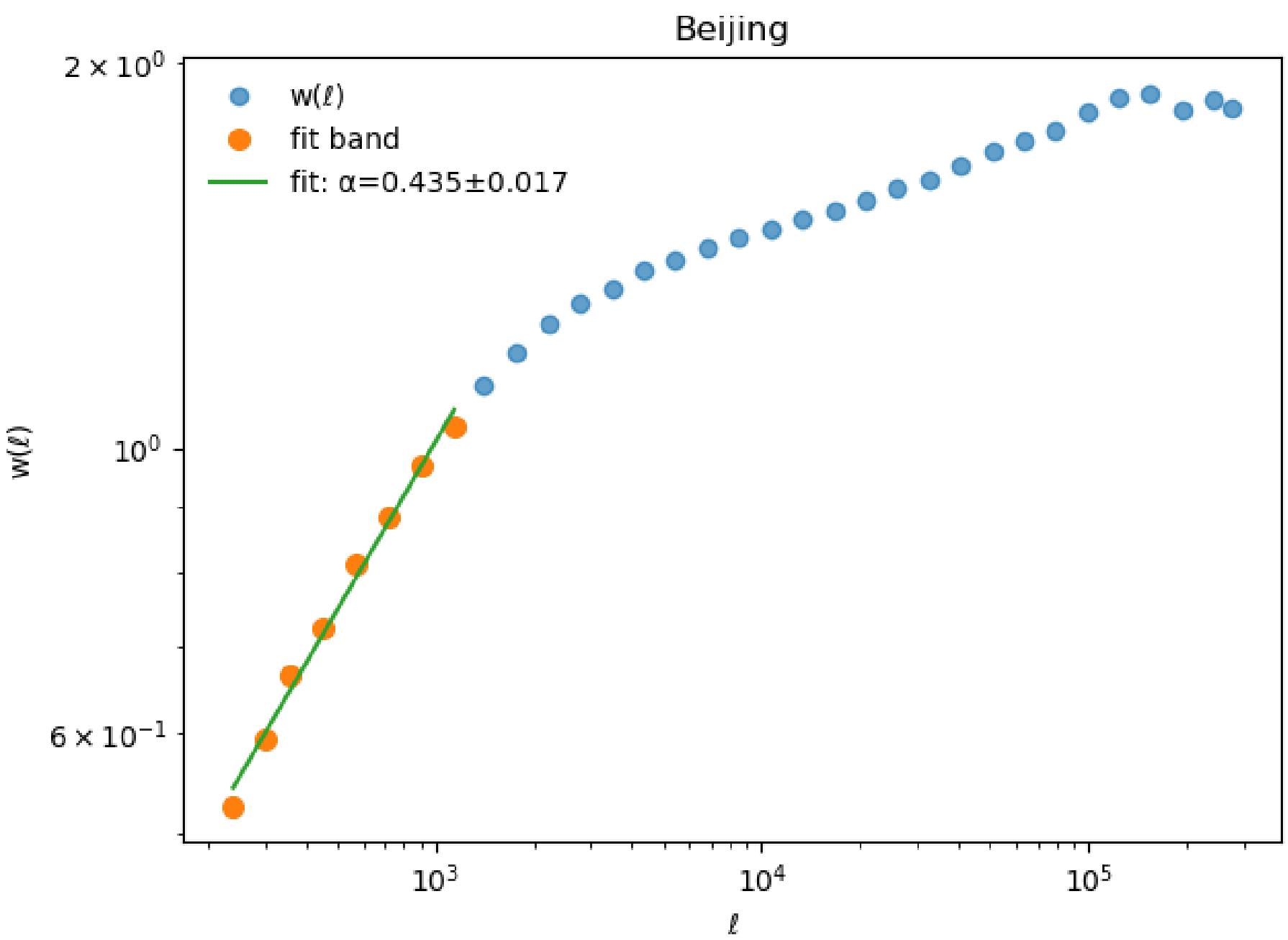

Supplementary Figure 11: Illustration of the pre-plateau computation of the scaling exponent α.

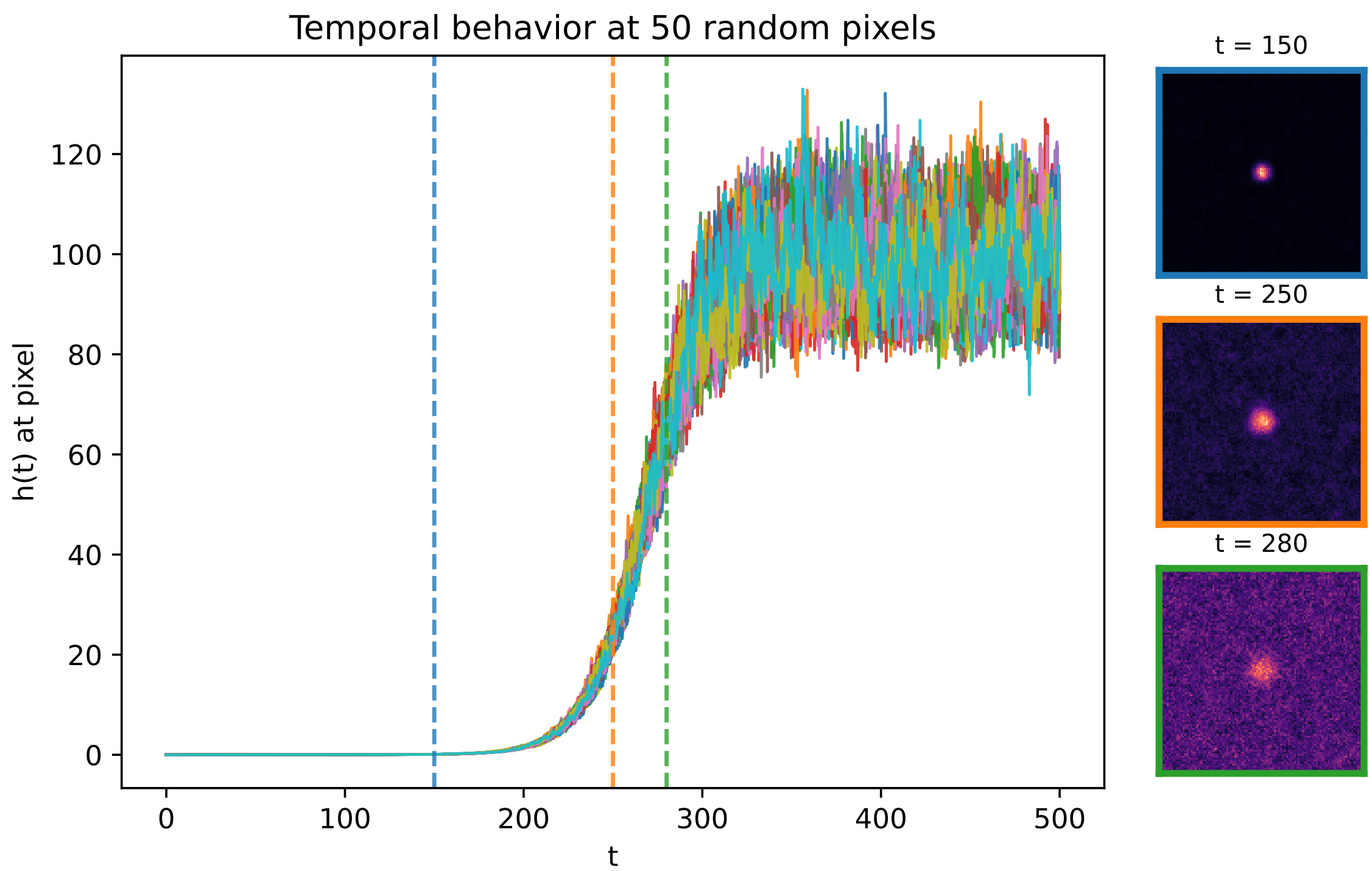

Supplementary Figure 12: Stochastic Fisher–KPP simulation (Eq. 18) on a 500×500 periodic lattice, initialized with a random field and a localized high-density seed at the domain center and with a capacity $K = 100$. The main panel shows the temporal evolution $h_i(t)$ at n randomly sampled pixels, exhibiting a characteristic (noisy) sigmoidal growth (GBM like behavior for early time) towards saturation due to stochasticity and diffusion. Colored dashed vertical lines mark the snapshot times. Insets (with matching colored frames) display the spatial structure $h(x, y, t)$ at the corresponding times, illustrating the spreading and pattern formation of the evolving field that tends then to be homogenous with height around the capacity $K = 100$.

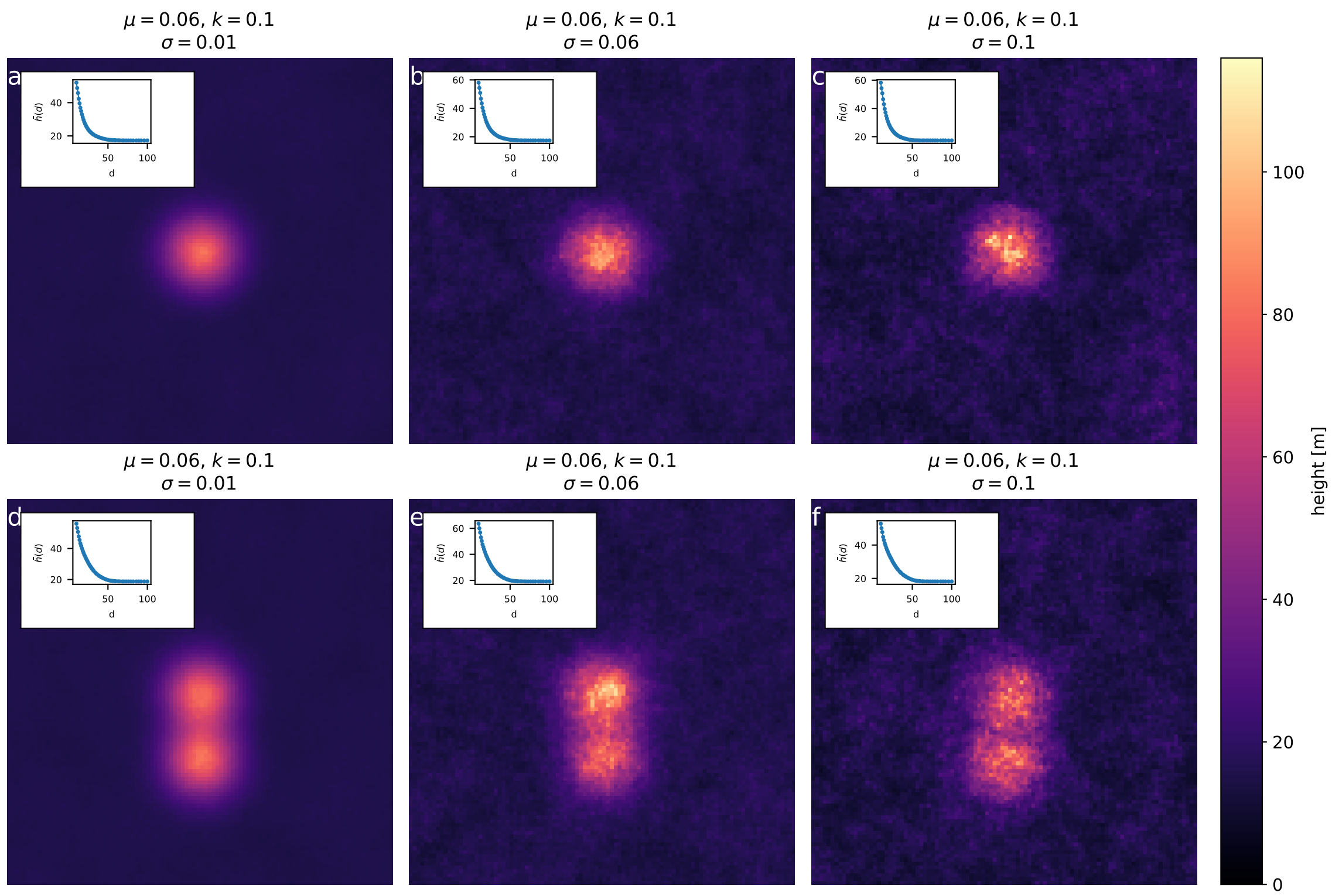

Supplementary Figure 13: Final height fields for different noise levels σ in monocentric (panels (a)–(c)) and polycentric (panels (d)–(f)) simulations. In each case the underlying dynamics use the same drift and coupling parameters; only σ is varied. The insets show the radially averaged building height $\overline{h}(d)$ as a function of the distance d to the centre of the domain, with a steeper decay in the monocentric case and a slightly flatter profile in the polycentric case.

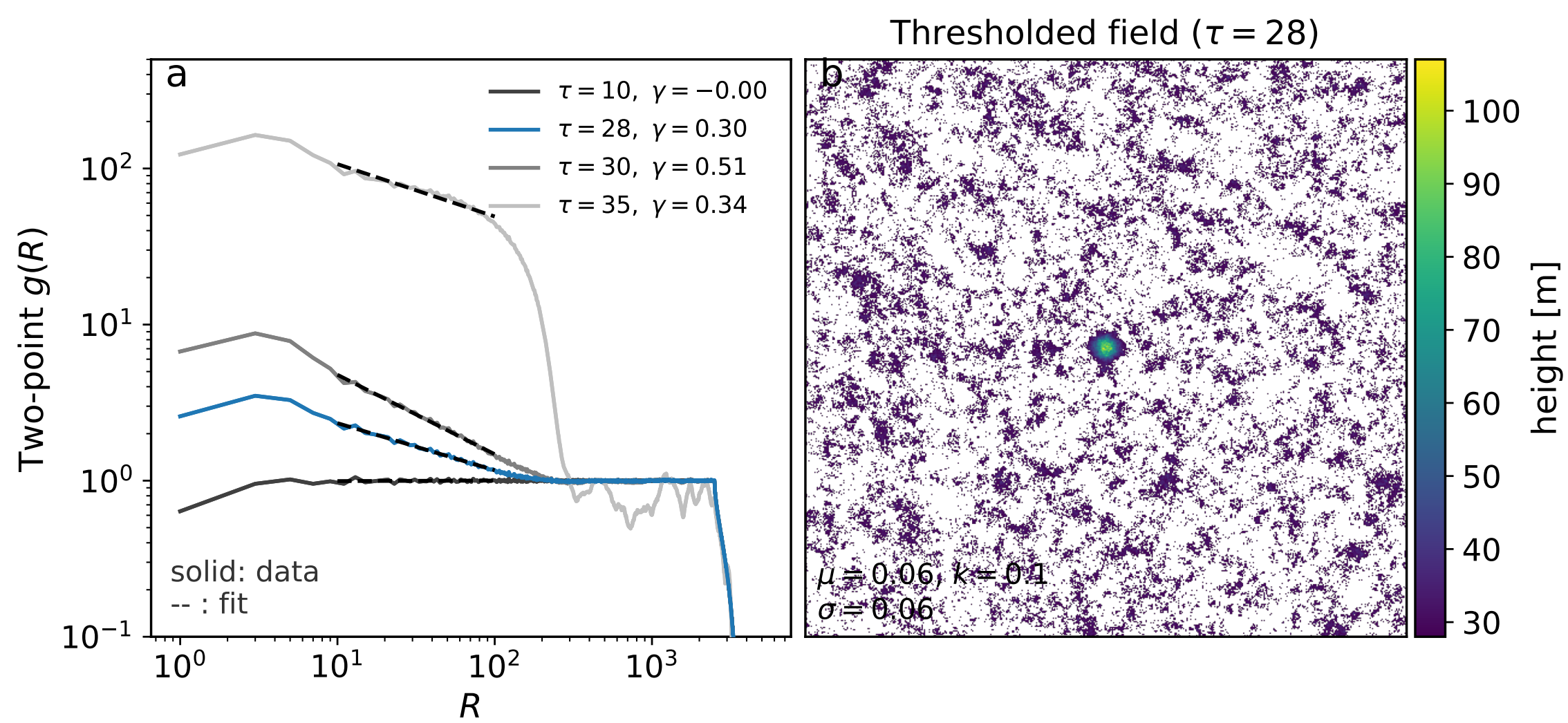

Supplementary Figure 14: (a) Pair-correlation $g(R)$ computed with the estimator of Eq. (13) in [3] on the locations of pixels whose height exceeds a given threshold τ in the long-time simulated field. (b) Corresponding spatial configuration of the thresholded set defined by $h > 28$ in the long-time field.

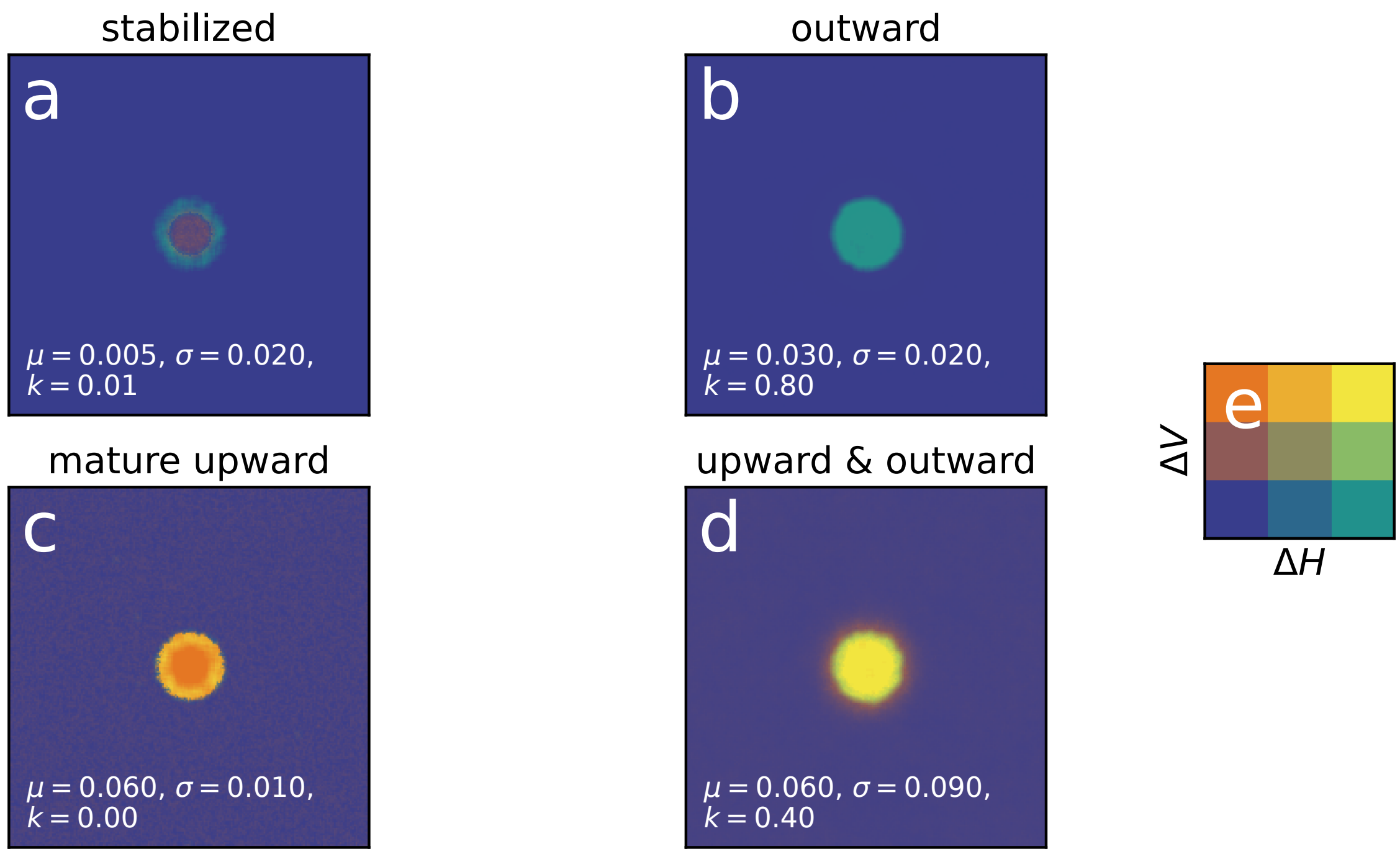

Supplementary Figure 15: Bivariate typology maps from the GBM+coupling model (Eq. 1). Panels (a–d) show the same simulation domain colored by a bivariate colormap encoding local vertical change ΔV (local growth in $\log(1+h)$ from the initial to the final state) and local horizontal change ΔH. The regimes are (a) stabilized, (b) outward, (c) mature upward, and (d) upward & outward. Panel (e) shows the bivariate colormap.

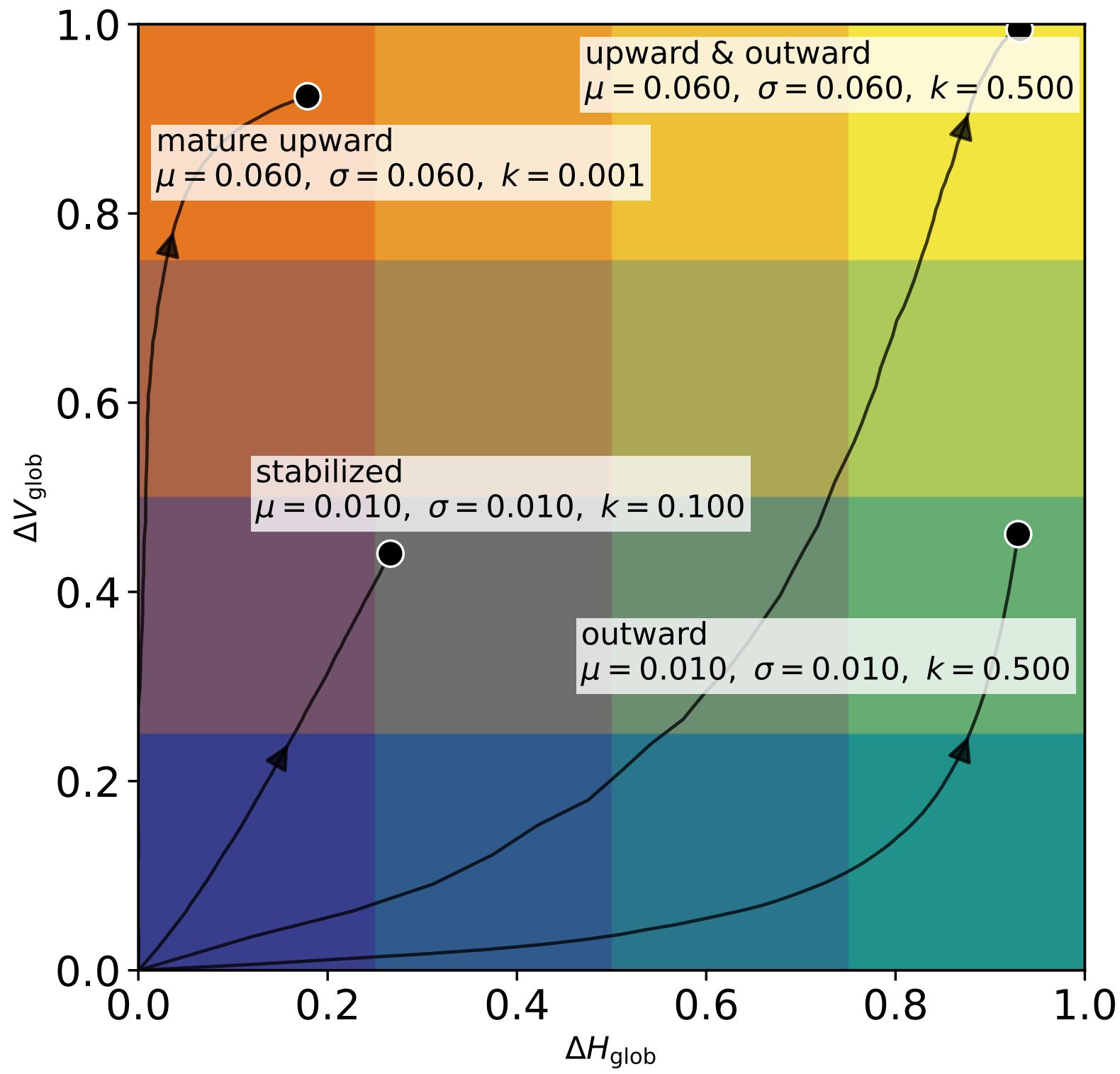

Supplementary Figure 16: Global growth trajectories in the $(\Delta H, \Delta V)$ plane. Each curve shows the time evolution of a simulated city under Eq. 1 for different set of parameters The arrows indicate the direction of the flow of time.

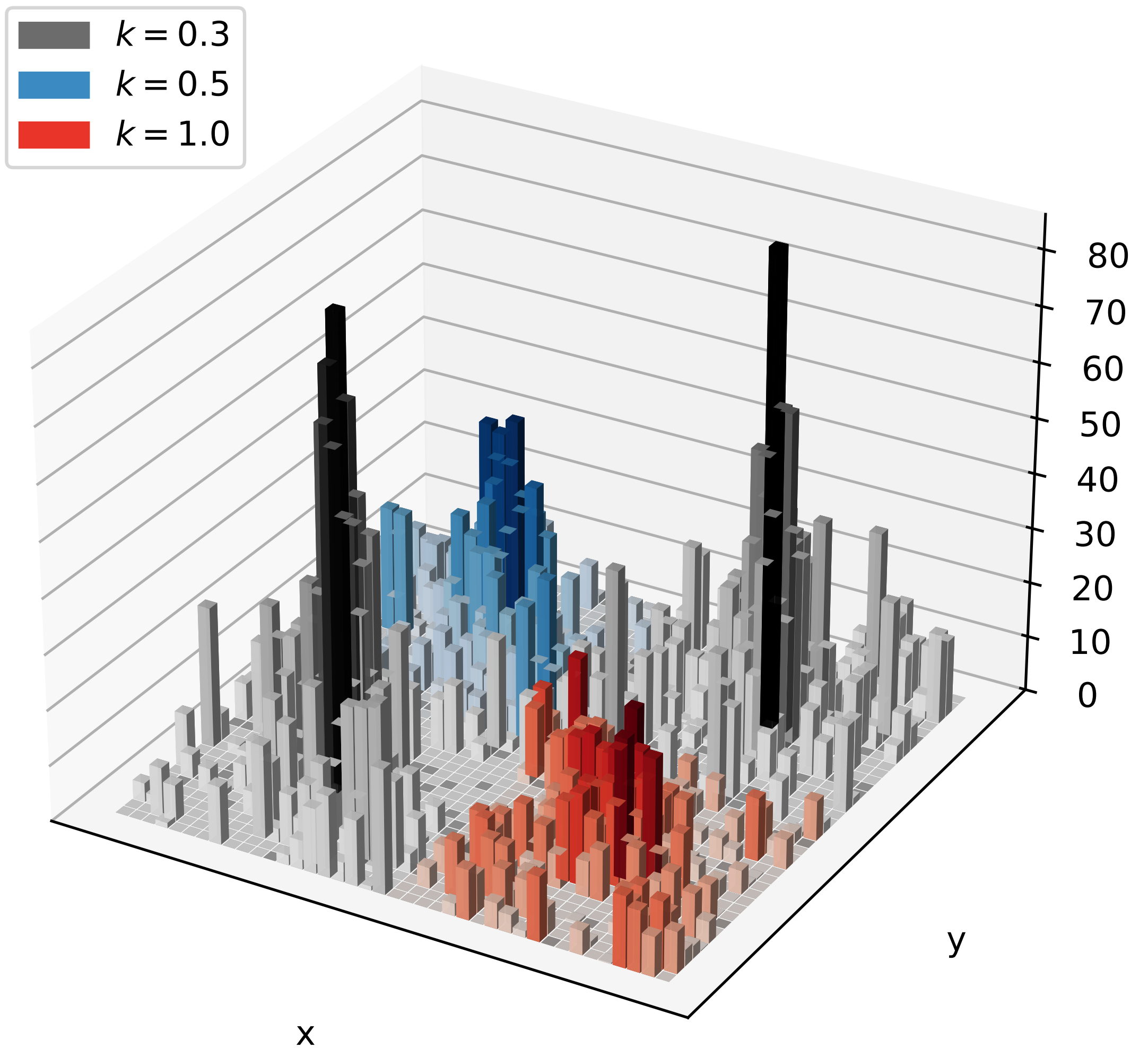

Supplementary Figure 17: Three-dimensional bar rendering of the simulated height field $h(x, T)$ on a coarse lattice, where bars represent local "building" heights and colors encode a simple zoning proxy through a piecewise-constant coupling field $k(x)$. The domain is partitioned into districts with $k \in \{0.3, 0.5, 1\}$ (grey/blue/red, respectively), and within each district the color intensity is scaled by the local height to preserve intra-zone contrasts. The plot illustrates how heterogeneous spatial coupling (interpretable as differential intra-urban connectivity or homogenisation across zones) produces visually distinct spatial structures under identical GBM parameters (μ, σ).

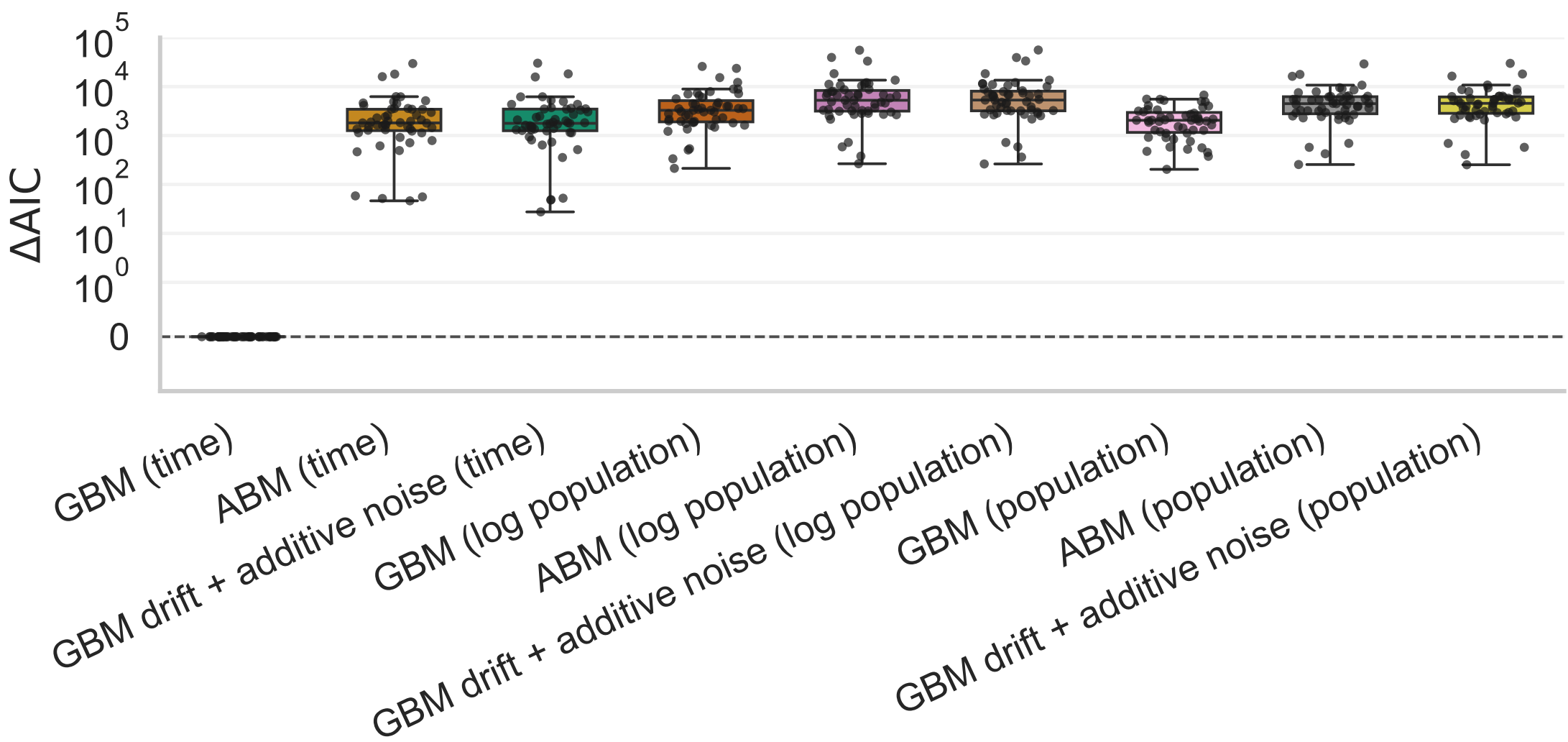

Supplementary Figure 18: For each city, we compute the AIC difference between each model and the best model in that city, $\Delta\text{AIC} = \text{AIC}_{\text{model}} - \text{AIC}_{\text{best, city}}$. GBM using calendar time as the clock is the best-fitting model and exhibits the lowest ΔAIC. Still using time as clock, the ABM model (which can be viewed as a simple "null" model with linear drift and additive noise) and the GBM with additive noise in time are the next best group. Models that use log-population and population as the clock perform systematically worse (both in GBM, ABM, and GBM+additive variants), indicating that population-driven clocks are not a good description of the observed building height dynamics.

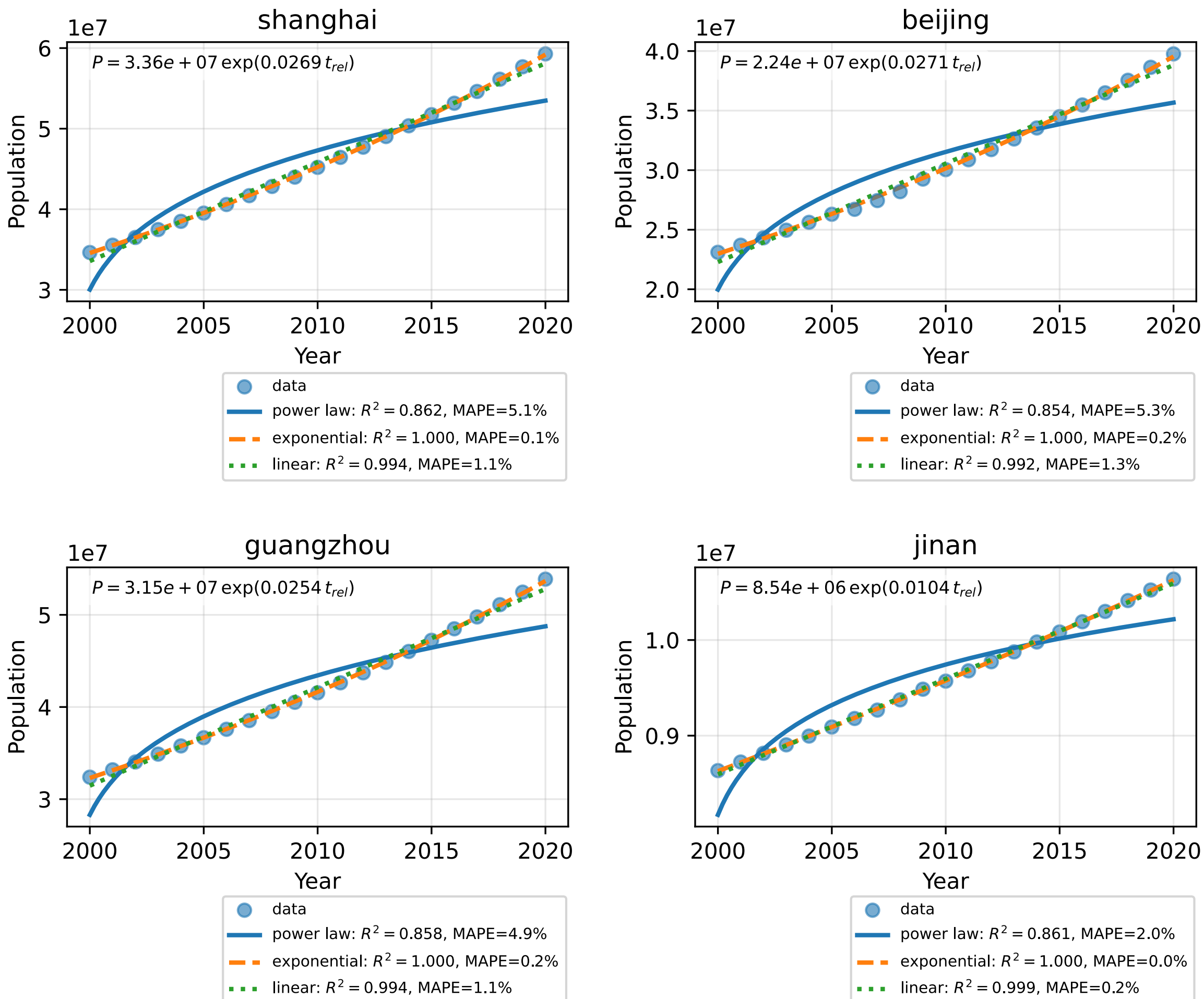

Supplementary Figure 19: Temporal evolution of the population the largest Chinese cities.

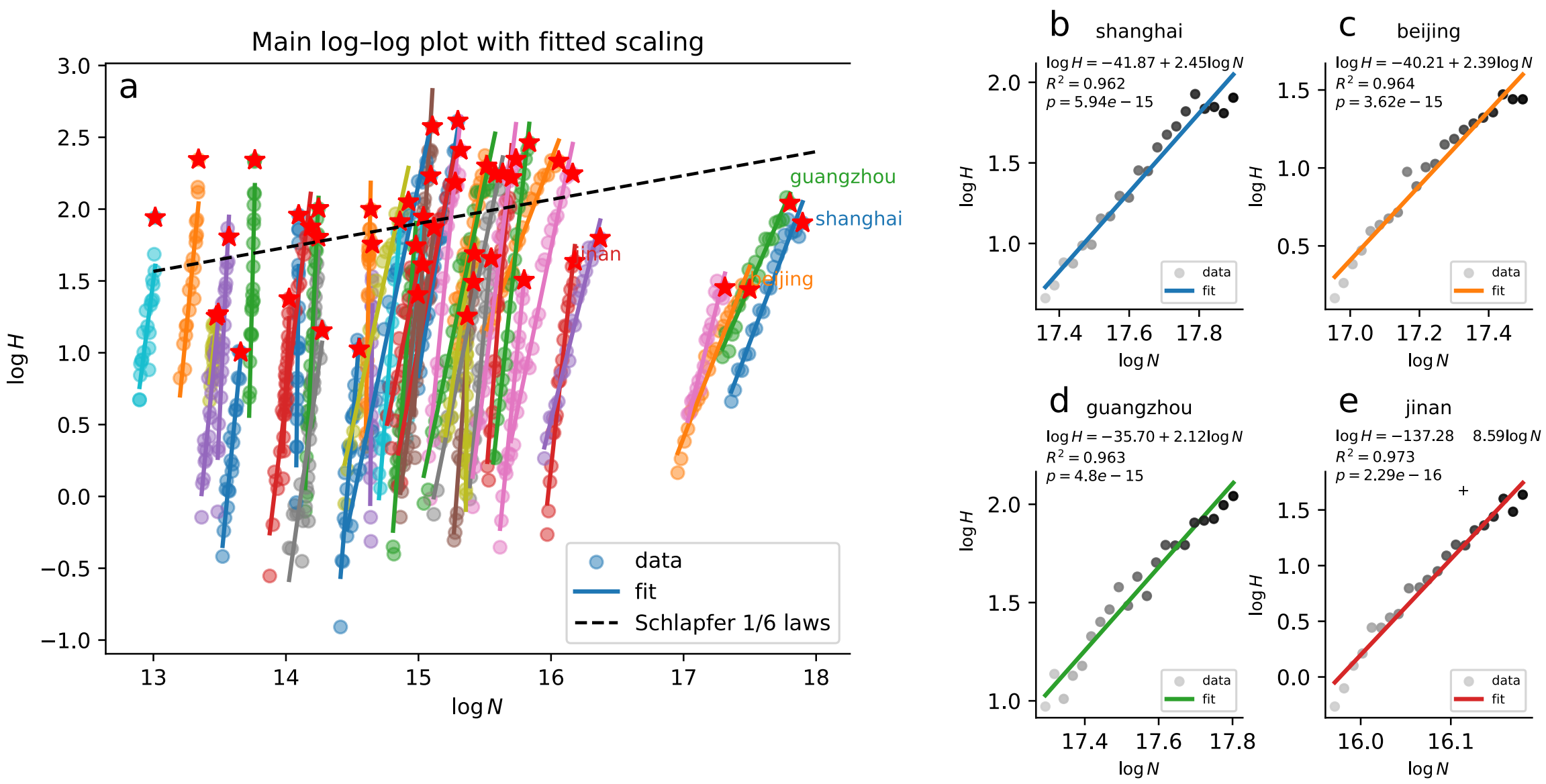

Supplementary Figure 20: (a) Log–log relation between mean building height $\log H$ and population $\log N$ for Chinese cities, with per-city power-law fits $\log H(t) = a_c + \beta_c \log N(t)$ shown in colour (longitudinal power-law). Red stars mark the 2020 mean values, and the dashed line indicates the Schläpfer et al. [1] scaling with exponent 1/6 (the cross-sectional). (b–e) Examples for four individual cities, showing their trajectories in $(\log N, \log H)$ over time (points shaded from light to dark with increasing year).

References

[1] Markus Schläpfer, Joey Lee, and Luís Bettencourt. Urban skylines: building heights and shapes as measures of city size. *arXiv preprint arXiv:1512.00946*, 2015.

[2] Gabriel M Ahlfeldt and Jason Barr. The economics of skyscrapers: A synthesis. *Journal of Urban Economics*, 129:103419, 2022.

[3] Michael Batty, Rui Carvalho, Andy Hudson-Smith, Richard Milton, Duncan Smith, and Philip Steadman. Scaling and allometry in the building geometries of greater london. *The European Physical Journal B*, 63(3):303–314, 2008.

[4] Richa Mahtta, Anjali Mahendra, and Karen C Seto. Building up or spreading out? typologies of urban growth across 478 cities of 1 million+. *Environmental Research Letters*, 14(12):124077, 2019.

[5] Joseph E Cavanaugh and Andrew A Neath. The akaike information criterion: Background, derivation, properties, application, interpretation, and refinements. *Wiley Interdisciplinary Reviews: Computational Statistics*, 11(3):e1460, 2019.

[6] Steve Frolking, Richa Mahtta, Tom Milliman, Thomas Esch, and Karen C Seto. Global urban structural growth shows a profound shift from spreading out to building up. *Nature Cities*, pages 1–12, 2024.

[7] Peimin Chen, Huabing Huang, Peng Qin, Xiangjiang Liu, Zhenbang Wu, Feng Zhao, Chong Liu, Jie Wang, Zhan Li, Xiao Cheng, et al. Characterizing dynamics of built-up height in china from 2005 to 2020 based on gedi, landsat, and palsar data. *Remote Sensing of Environment*, 325:114776, 2025.

[8] David Freedman and Persi Diaconis. On the histogram as a density estimator: L 2 theory. *Zeitschrift für Wahrscheinlichkeitstheorie und verwandte Gebiete*, 57 (4):453–476, 1981.

[9] Jianhua Lin. Divergence measures based on the shannon entropy. *IEEE Transactions on Information theory*, 37(1):145–151, 2002.
