# Supplementary material for: Dynamic roughening of cities driven by multiplicative noise
Source: Nat Commun. 2026 Jun 25;17:7974. doi: 10.1038/s41467-026-74456-4 (PMC13448524; doi:10.1038/s41467-026-74456-4)
Supplement: Supplementary file 3 [file 41467_2026_74456_MOESM3_ESM.pdf]

nature portfolio

Peer Review File

Dynamic roughening of cities driven by multiplicative noise

Corresponding Author: Dr Martin Hendrick

This file contains all reviewer reports in order by version, followed by all author rebuttals in order by version.

Attachments originally included by the reviewers as part of their assessment can be found at the end of this file.

Version 0:

Reviewer comments:

Reviewer #1

(Remarks to the Author)
This study proposes to model skyline height evolution as a zero-dimensional Geometric Brownian Motion (GBM). Utilizing remote sensing data, they calibrate their model on the data, which show good agreement in South East Asia, both at intra and inter-urban scales.
Moreover, in order to model the height-height correlations, they add a discrete Laplacian term to the GBM model, in order to incorporate spatial correlations into the model. This model, when taken in the mean-field (MF) approximation, 1) admits an analytical stationary distribution 2) is capable of reproducing morphologies 3) reduces to the Stochastic Heat equation (SHE). The authors argue that this minimal, tractable model, balanced by the interactions between multiplicative noise and spatial diffusion, offers a model allowing to understand the dynamics of urban height growth. They suggest
1) gross domestic product (GDP) and height are affinely correlated
2) GBM is a good proxy for the dynamics of both of these quantities, that are (typically) difficult to obtain from direct observations
3) that all cities long-time height distribution is given by the stationary distribution, only parametrized by r
4) this parameter r places where the city distributes on the ‘noise’ – ‘diffusion’ spectrum

The data processing, analysis (theoretical and computational) seems sound, and this surface growth physics-based approach to urban heights dynamics is new and relevant to the field. The technical details are remarkably well explained, both for the data processing and the analytical derivation. Despite this, I have several major conceptual and technical concerns, which I believe should be answered before the manuscript can be accepted for publication in Nature Communications.

Major comments :

1) the urban economics literature typically reports heterogenous urban height profiles, peaked at the economic center(s) and decreasing away from it/them (see e.g. [G. M. Ahlfeldt, J. Barr,
The economics of skyscrapers: A synthesis,
Journal of Urban Economics] and [M. Schlapfer et al., Urban Skylines: building heights and shapes as measures of city size, arxiv]). Is this stylized fact reproduced by the model, for instance in the case of a monocentric city ?

2) More generally, the model has 3 parameters (mu, sigma, k) and essentially reproduces 3-4 stylized facts of vertical dynamics : h(t), var(h)(t), and the morphological similarity (statically in the long-time limit given by p_\infty and dynamically through the fit presented in Fig.3) . The manuscript would benefit by checking whether other empirical facts known of urban morphologies and their dynamics are reproduced by the model (or not). For instance, [Batty M. Scaling and allometry in the building geometries of Greater London, The European Physical Journal B] reports long-range height-height correlations in London’s buildings heights (with an exponent $\gamma\approx0.23).

3) Urban science typically uses population as a natural clock, instead of time, ([Batty M. The size, scale, and shape of cities. Science.], [Luís M. A. Bettencourt,The Origins of Scaling in Cities.Science], [Marquis U., Barthelemy M., Modeling the spatial growth of cities, arxiv
]). This allows to describe cities characteristics, such as gross domestic product (GDP), road length, number of crimes/patents, built area... through cross-sectional relations like

Y \sim N^{\beta}. (1)
For instance, the M. Schlapfer reference mentioned above finds an exponent of \approx 1/6 for the average height. How does the longitudinal findings reported by the authors translate in 'urban scaling' vocabulary ?

4) The authors claim that their results align with the findings of ref.18 ([Kinetic roughening of the urban skyline...]). We cannot agree with this statement : ref.18 finds KPZ/EW scaling for h, while the manuscript shows KPZ scaling for \phi = \log h. Moreover, in the reviewer's view, the exponents measured in Fig.5a do not necessarily correspond to \alpha (the roughness exponent of surface growth physics), but rather to an \alpha_loc, describing the scaling of roughness at scales much smaller than the system size (see e.g [J.J Ramasco et al.,Generic Dynamic Scaling in Kinetic Roughening, PRL]). Could the authors clarify this point? Furthermore, for the sake of self-containment, an improved discussion on known universality classes in 2+1D kinetic roughening would help the reader (e.g known universality classes, exponents values).

5) Even though the proposed model allows both for inter- and intra- urban prediction, suggesting self-similarity of growth processes at different scales, it is unclear to the reviewer how this model (with 3 parameters) explains better the dynamics of intra-urban vertical growth than simpler models of 2+1d surface growth (e.g. solid-on-solid model, or other non-conservative equations). In particular, this model predicts (when \gamma>0) unbounded growth for \overline{h} when t \to \infty, which allows well to capture early time dynamics but is contradictory with the fact that the cost of buildings scale like degree-two polynomial function [see e.g. Ahfeldt and Barr and references therein] and hence that a cost-based reasoning would rather lead to conjecture an equilibrium between the benefits given by heights (density, economic productivity) and cost of building high – even at 'small' h.

6) The discussion on the (s)FKPP equation for 'mature cities' is unclear and would deserve a more nuanced and detailed discussion. FKPP fronts (eq.18 without the noise term) propagate at asymptotic speed 2\sqrt{D \mu}and, with a stable phase h = K invading a h=0 unstable phase. Hence this models cities heights dsitribution as essentially bimodal, with a very small front (as it typically decreases exponentially) separating the phases. This model does not seem at first very convincing and a discussion explaining better 1) its derivation 2) what is the effect of noise on the reaction-diffusion fronts (shape and speed) 3) why it describes cities would help improving this part of the manuscript.

7) what are the geometric consequences of this modelization ? How does the total volume scale, and how does the total ground surface scale ? What is the horizontal sprawl speed ?

Minor comments:

- in the introduction, the causes of urban expansions should be discussed more accurately (with references)
- discuss the values of RMSE (Fig.3) – how can I assess whether the fit provided by the model is good (Fig,3) ? Compare to a null/non-predictive model ?
- what about the effects of height restriction ? Typically discussed in urban economics, in the context of efficiency of cities.
- growth occurs on the horizontal-vertical axis according to a certain typology [Mahtta.R et al, Building up or spreading out? Typologies of urban growth across 478 cities of 1 million+, ERL] – where is this model situated on this typology?
- the authors mention 'KPZ' : the KPZ equation maps to various universality classes when the noise nature changes (annealed, quenched, columnar-quenched, spatio-temporaly correlated...). – it would be worth mentioning it and explaining the scaling differences between the various universality classes.
- What is the typical time scale of relaxation to the profile to p_\infty ?

Reviewer #2

(Remarks to the Author)

Version 1:

Reviewer comments:

Reviewer #1

(Remarks to the Author)
The authors responded all my comments and modified the manuscript accordingly; I recommend publication.

Reviewer #2

(Remarks to the Author)
I am satisfied with the improvement made by the authors. Therefore, I suggest the article be accepted for publication.

Open Access This Peer Review File is licensed under a Creative Commons Attribution 4.0 International License, which permits use, sharing, adaptation, distribution and reproduction in any medium or format, as long as you give appropriate credit to the original author(s) and the source, provide a link to the Creative Commons license, and indicate if changes were made.
In cases where reviewers are anonymous, credit should be given to 'Anonymous Referee' and the source.
The images or other third party material in this Peer Review File are included in the article's Creative Commons license, unless indicated otherwise in a credit line to the material. If material is not included in the article's Creative Commons license and your intended use is not permitted by statutory regulation or exceeds the permitted use, you will need to obtain permission directly from the copyright holder.
To view a copy of this license, visit https://creativecommons.org/licenses/by/4.0/

In this manuscript the authors follow cities' skyline as a moving front, which is followed through the evolution of the buildings' heights dynamics. They model this process as a zero-dimensional geometric Brownian motion with multiplicaltive noise, which is augmented with spatial coupling. They then show that this interplay of the two aforementioned effects drives the morphological changes and that the Kardar-Parisi-Zhang (KPZ) equation is recovered in the continuum limit.

The growth of the skyline has been previously shown to follow either the KPZ or the Edwards-Wilkinson (EW) universality classes [1], and in that respect the work is trying to build a ground-up model linking model parameters to economic productivity, which is an intersting take on explainability of the dynamics. The work is an attempt to explain an already observed feature of the skyline using a bottom-up model. The findings, though interesting, represent an extension rather than a breakthrough.

The following questions arise:

1- The KPZ is a universality class and thus it subsumes numerous alternative growth models. Subsequently, the current proposed model is one of many that could explain the dynamics and it would be valuable to compare the it with equally valid models such as restricted solid on solid (RSOS), Eden model, asymmetric simple exclusion (ASEP) [2]. A likelihood measure should be provided in support of this model as opposed to others.

2- There are multiple parameters in the model, each of which could have its own economic/regulatory correspondances. In particular k could reflect regulations imposed on building heights. For example, when the authors follow the mean-field formulation, the coupling k effectively multiplies hbar, which is a regulatory value on mean building height in the city. No comments on this were made.

3- It would be valuable if the authors could look at cities where k changes both temporally and spatially and follow the evolution of the GBM. Can the effect of a changing k be modeled? This would be a more intersting dynamical evolution of the growth.

4- It would be also valualble to link varrying k to zoning within cities? intracity clustering into residential, industrial, mixed…

The whole work is technically sound however the contribution does not seem to introduce fundamentally new concepts. I recommend revisiting the work and addressing the comments and questions provided to substantiate the work with more evidence and relevance to the urban complexity community.

[1] Najem, S., Krayem, A., Ala-Nissila, T., & Grant, M. (2020). Kinetic roughening of the urban skyline. *Physical Review E*, *101*(5), 050301.

[2] Barabási, A. L., & Stanley, H. E. (1995). *Fractal concepts in surface growth*. Cambridge university press.

Response to reviewers' comments

Martin Hendrick and Gabriele Manoli

March 1, 2026

Reviewer #1

Remarks to the Author: This study proposes to model skyline height evolution as a zero-dimensional Geometric Brownian Motion (GBM). Utilizing remote sensing data, they calibrate their model on the data, which show good agreement in South East Asia, both at intra and inter-urban scales. Moreover, in order to model the height-height correlations, they add a discrete Laplacian term to the GBM model, in order to incorporate spatial correlations into the model. This model, when taken in the mean-field (MF) approximation, 1) admits an analytical stationary distribution 2) is capable of reproducing morphologies 3) reduces to the Stochastic Heat equation (SHE). The authors argue that this minimal, tractable model, balanced by the interactions between multiplicative noise and spatial diffusion, offers a model allowing to understand the dynamics of urban height growth. They suggest 1) gross domestic product (GDP) and height are affinely correlated 2) GBM is a good proxy for the dynamics of both of these quantities, that are (typically) difficult to obtain from direct observations 3) that all cities long-time height distribution is given by the stationary distribution, only parametrized by r 4) this parameter r places where the city distributes on the 'noise' – 'diffusion' spectrum

The data processing, analysis (theoretical and computational) seems sound, and this surface growth physics-based approach to urban heights dynamics is new and relevant to the field. The technical details are remarkably well explained, both for the data processing and the analytical derivation. Despite this, I have several major conceptual and technical concerns, which I believe should be answered before the manuscript can be accepted for publication in Nature Communications.

Major comments:

C1:

> **Comment**
>
> the urban economics literature typically reports heterogenous urban height profiles, peaked at the economic center(s) and decreasing away from it/them (see e.g. [G. M. Ahlfeldt, J. Barr, The economics of skyscrapers: A synthesis, Journal of Urban Economics] and [M. Schlapfer et al., Urban Skylines: building heights and shapes as measures of city size, arxiv]). Is this stylized fact reproduced by the model, for instance in the case of a monocentric city ?

Reply. We thank the referee for pointing out this important connection to the urban economics literature on vertical land use. Indeed, both Ahlfeldt and Barr [2022] and Schläpfer et al. [2015] report height profiles that are peaked at the economic centre and decrease with

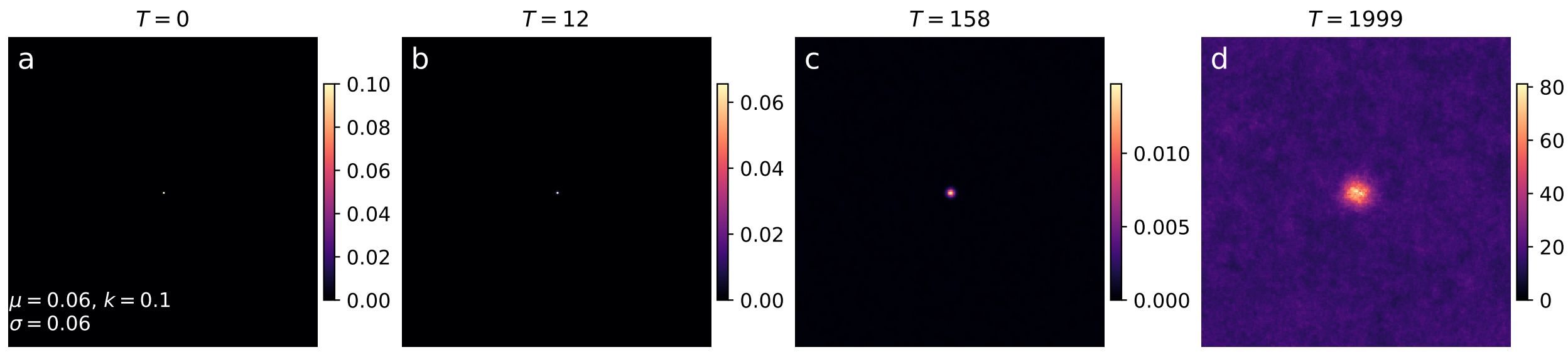

Figure 1: Monocentric simulation on a 2D lattice. One realisation of the stochastic height dynamics with parameters $\mu = 0.06$, $\sigma = 0.06$, and $k = 0.1$. Snapshots at increasing times (from left to right) show the emergence and persistence of a monocentric pattern, with taller buildings near the centre and decreasing heights with distance.

distance. We have checked that our model is able to reproduce this stylized fact in a simple monocentric (and polycentric) setting.

More precisely, we simulated the model on a two-dimensional lattice, using parameters of the same order as used in the paper ($\mu = 0.06$, $\sigma = 0.06$, $k = 0.1$). At time $t = 0$, we initialise the height field with small random fluctuations everywhere and impose a single higher value at the centre of the domain. Figure 1 shows a few snapshots of one realisation. As time evolves, we observe the emergence of a clear monocentric pattern: the tallest buildings concentrate near the centre, and the typical height decreases as one moves away from it. In Figure 2 we show several realisations for different values of σ; the inset reports the radial average of height as a function of the distance d to the centre. The average height profile decays smoothly with d, in qualitative agreement with the empirically observed monocentric height gradients. We also illustrate the polycentric case by running simulations with two nearby high initial values (two "seeds") instead of one. In this case, if we define the centre as the middle of the domain, the radial average of height still decays with distance, but with a slightly flatter profile than in the strictly monocentric case.

These numerical experiments are not a full calibration to the empirical gradients reported by Ahlfeldt & Barr or Schläpfer et al., but they demonstrate that the generic stochastic dynamics we propose are compatible with the standard monocentric height profiles documented in the urban economics literature.

We added a short paragraph to show this phenomena in the revised Supplementary Information (SI) document **(SI Lines 9-24)**.

C2:

Comment

More generally, the model has 3 parameters (mu, sigma, k) and essentially reproduces 3-4 stylized facts of vertical dynamics : h(t), var(h)(t), and the morphological similarity (statically in the long-time limit given by p_∞ and dynamically through the fit presented in Fig.3) . The manuscript would benefit by checking whether other empirical facts known

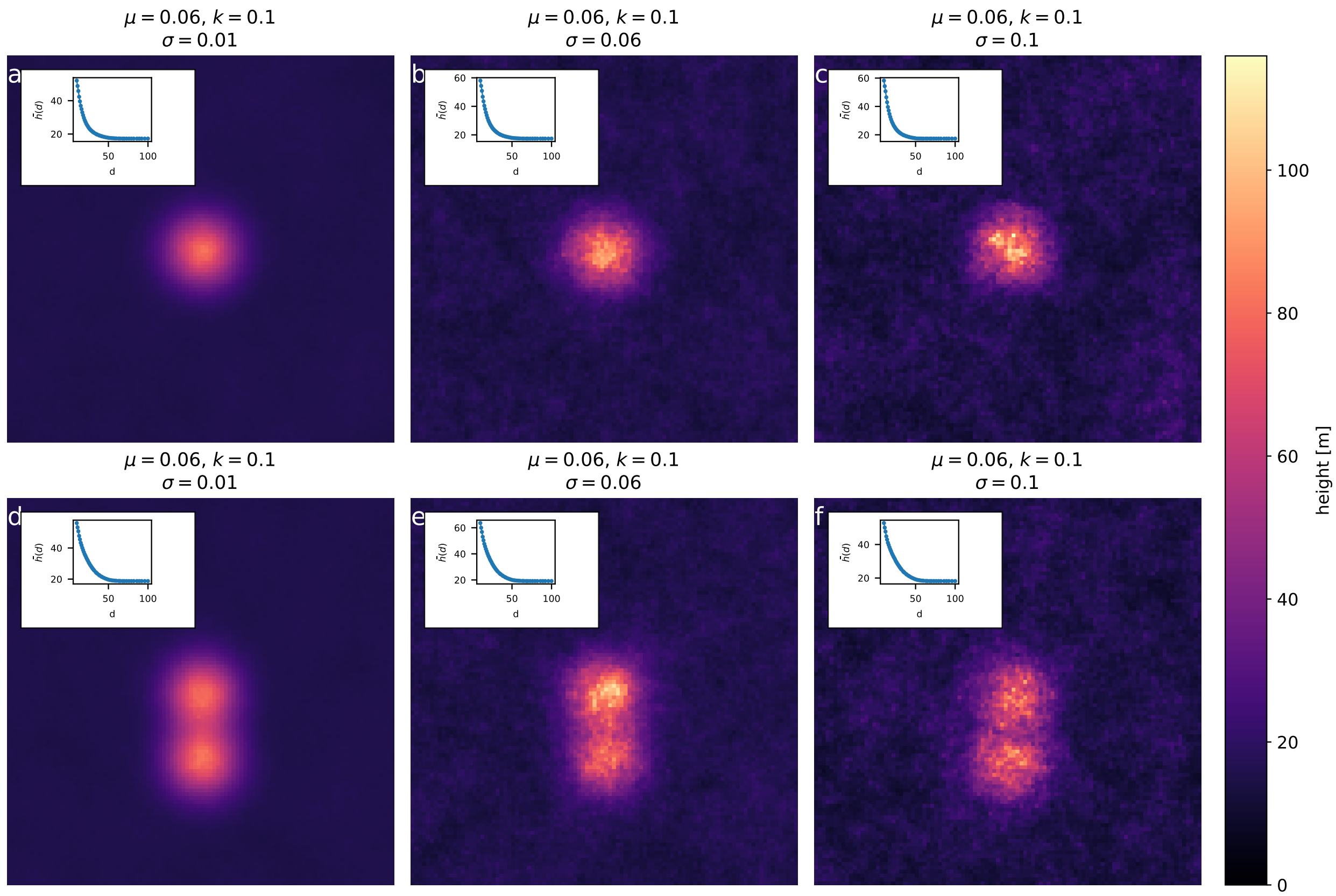

Figure 2: Final height fields for different noise levels σ in monocentric (panels (a)–(c)) and polycentric (panels (d)–(f)) simulations. In each case the underlying dynamics use the same drift and coupling parameters; only σ is varied. The insets show the radially averaged building height $\overline{h}(d)$ as a function of the distance d to the centre of the domain, with a steeper decay in the monocentric case and a slightly flatter profile in the polycentric case.

> of urban morphologies and their dynamics are reproduced by the model (or not). For instance, [Batty M. Scaling and allometry in the building geometries of Greater London, The European Physical Journal B] reports long-range height-height correlations in London's buildings heights (with an exponent $\gamma \approx 0.23$).

Reply. We thank the reviewer for this suggestion. We agree that validating additional empirical regularities beyond the first moments and morphological similarity is important.

In Batty et al. [2008], the two-point correlation function, $g(R)$ (Eq. 13 in Batty's paper), is evaluated on the locations of a selected subset of buildings (the $K = 10^5$ tallest), rather than on continuous height values. This selection is equivalent to introducing an implicit height cutoff τ. To ensure a direct comparison, we apply the same procedure to our simulations: we threshold the long-time simulated height field at a value τ and compute $g(R)$ on the resulting point pattern using Batty's annulus-based estimator.

For a representative simulation initialised from a random field with an additional higher-valued seed at the domain centre, and using model parameters in the range calibrated from East-Asian cities, we find threshold around $\tau = 28$ an intermediate-range decay compatible with an effective power law, $g(R) \sim R^{-\gamma}$ (Fig. 3). We can observe that depending on the chosen value of τ the effective power law varies, underlying the limited utility of power law fit without considering finite size and aggregation effects.

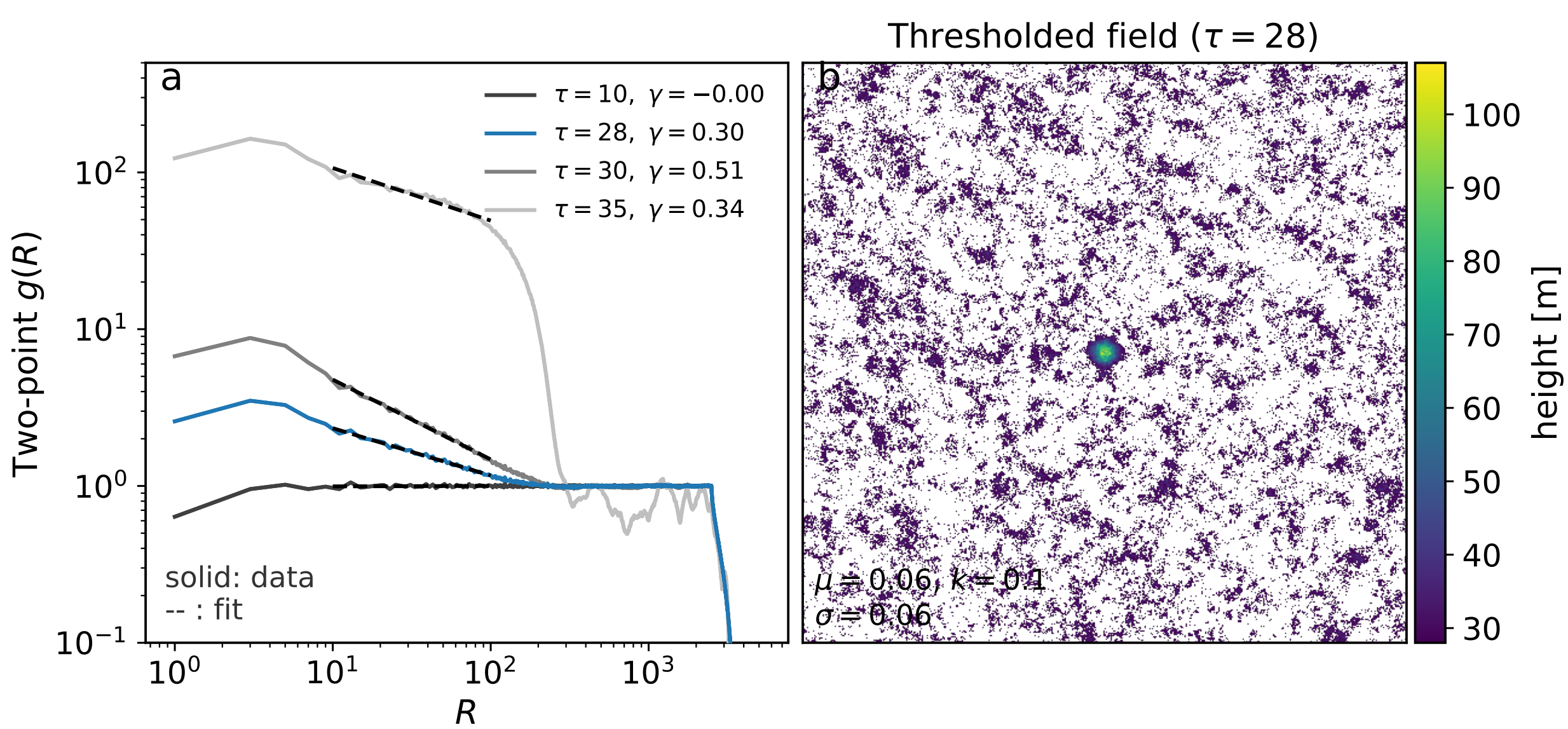

Figure 3: (a) Pair-correlation $g(R)$ computed with the estimator of Eq. (13) in Batty et al. [2008] on the locations of pixels whose height exceeds a given threshold τ in the long-time simulated field. (b) Corresponding spatial configuration of the thresholded set defined by $h > 28$ in the long-time field.

This observation is now included in the revised SI document **(SI Lines 25-36)**.

C3:

Comment

Urban science typically uses population as a natural clock, instead of time, ([Batty M. The size, scale, and shape of cities. Science.], [Luís M. A. Bettencourt,The Origins of Scaling in Cities.Science], [Marquis U., Barthelemy M., Modeling the spatial growth of cities, arxiv]). This allows to describe cities characteristics, such as gross domestic product (GDP), road length, number of crimes/patents, built area... through cross-sectional relations like Y $\sim N^{\beta}$. (1) For instance, the M. Schlapfer reference mentioned above finds an exponent of $\approx 1/6$ for the average height. How does the longitudinal findings reported by the authors translate in 'urban scaling' vocabulary ?

Reply. We thank the referee for this important remark. We fully agree that most of the urban scaling literature takes total population N as the natural "size" variable and studies cross-sectional relations of the form

$$Y \sim N^{\beta}$$

at a given date. Our contribution is complementary as we work on a longitudinal approach. So, a crucial question is to test which variable acts as the most appropriate "clock" for the stochastic dynamics of building heights within cities. Here are three arguments in favour of using time as clock:

1. **We explicitly tested population (and log-population) as alternative clocks.** For each city and each building trajectory we constructed height increments and compared a family of stochastic models where the "time increment" is respectively

   $$\Delta t, \qquad \Delta N, \qquad \Delta \log N.$$

   For each of these clocks we estimated GBM-type, ABM-type (Arithmetic Brownian motion), and "GBM with additive noise" models and compared them by their log-likelihood and AIC (Akaike Information Criterion Cavanaugh and Neath [2019]). For each city and each model we construct increments across all buildings (using the appropriate clock $X \in \{t, P, \log P\}$), estimate model's parameters by maximum likelihood, and record the maximized log-likelihood $\ell_{\text{city, model}}$. The AIC for a given city and model is

   $$\text{AIC}_{\text{city, model}} = 2k - 2\,\ell_{\text{city, model}},$$

   where k is the number of free parameters. And then, for each city we identify the best-fitting model as the one with the smallest AIC, and define

   $$\Delta\text{AIC}_{\text{city, model}} = \text{AIC}_{\text{city, model}} - \min_{\text{model'}} \text{AIC}_{\text{city, model'}}.$$

   By construction, the best model in each city has $\Delta\text{AIC} = 0$, and larger ΔAIC indicate worse penalized fit relative to the city-specific optimum.

   Across the ensemble of cities, the calendar-time specifications for height (or log-height) systematically achieve lower AIC than the corresponding population-clock or log-population-clock models. In other words, treating N as the clock gives a worse probabilistic description of the observed height increments than treating t as the clock. See Figure 4.

2. **Population is not an exogenous clock for height in our setting.** In the scaling literature, N is usually treated as a macro-state: one regresses city-level quantities on N

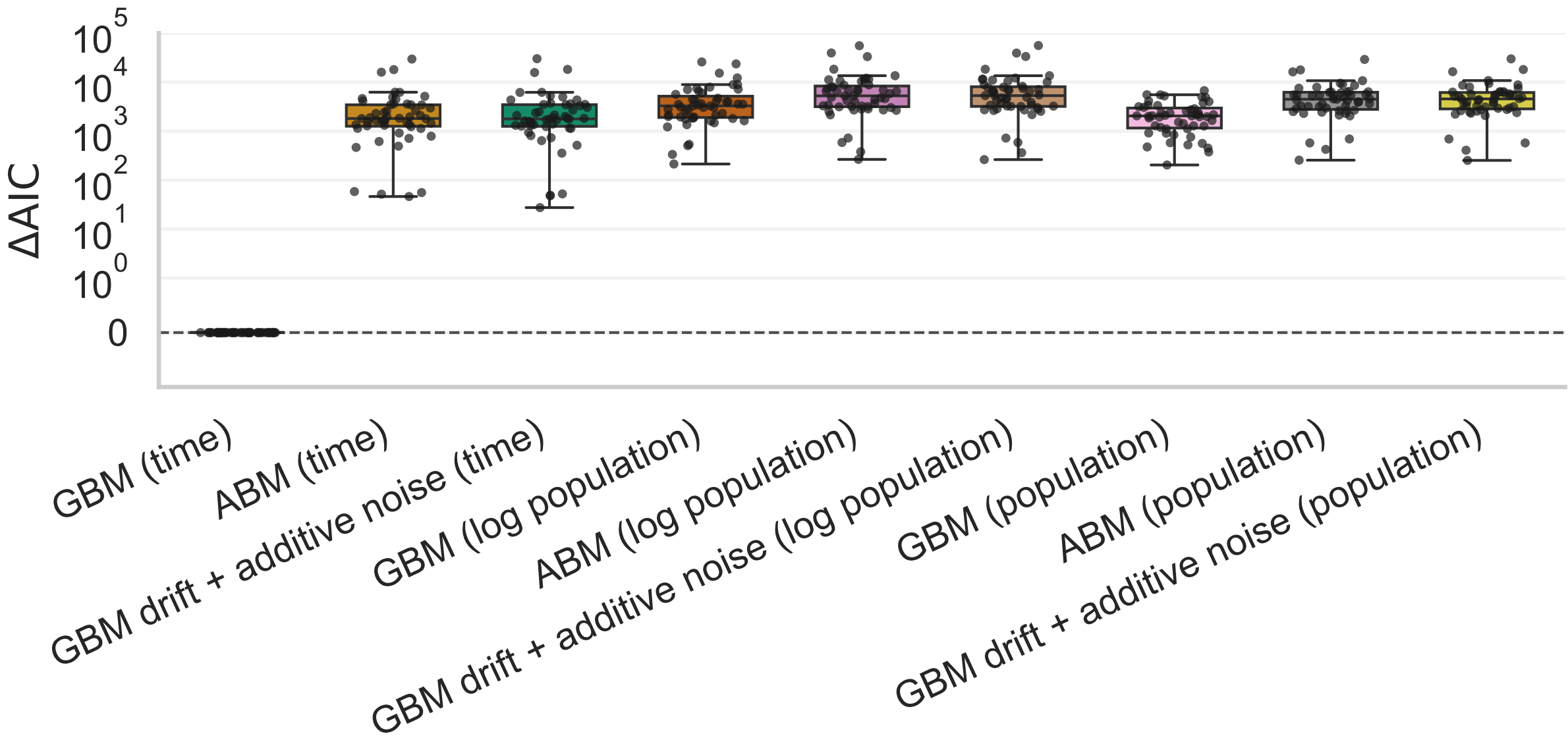

Figure 4: For each city, we compute the AIC difference between each model and the best model in that city, $\Delta\text{AIC} = \text{AIC}_{\text{model}} - \text{AIC}_{\text{best, city}}$. GBM using calendar time as the clock is the best-fitting model and exhibits the lowest ΔAIC. Still using time as clock, the ABM model (which can be viewed as a simple "null" model with linear drift and additive noise) and the GBM with additive noise in time are the next best group. Models that use log-population and population as the clock perform systematically worse (both in GBM, ABM, and GBM+additive variants), indicating that population-driven clocks are not a good description of the observed building height dynamics.

across many cities at a fixed time. In our dataset, however, $N_c(t)$ (the subscript c aims to label cities) is itself partly generated by the vertical built form, since population capacity depends on building heights and densities. In other words, $H_c(t)$ and $N_c(t)$ co-evolve and influence each other, so using N as a "clock" for H amounts to parametrizing one endogenous state variable by another, rather than by an external time parameter.

3. **Longitudinal exponents are purely kinematic and differ from cross-sectional scaling exponents.** Empirically, for each city c we observe that both mean height and population grow approximately exponentially in calendar time (Figure 5),

$$\bar{H}_c(t) \simeq H_{0,c}\, e^{\mu_c t}, \qquad N_c(t) \simeq N_{0,c}\, e^{\eta_c t},$$

with city-specific growth rates μ_c (for height) and η_c (for population). Eliminating time t along the trajectory of a single city then yields

$$\bar{H}_c(t) \propto N_c(t)^{\beta_c^{\text{traj}}}, \qquad \beta_c^{\text{traj}} = \frac{\mu_c}{\eta_c}.$$

The longitudinal exponent β_c^{traj} is thus a purely kinematic quantity: it is just the ratio of the city's height and population growth rates.

By contrast, the familiar cross-sectional scaling exponent β_{between} at a given date t_0 is defined as the slope of the regression of $\log \bar{H}_c(t_0)$ on $\log N_c(t_0)$ across cities,

$$\beta_{\text{between}}(t_0) = \frac{\text{Cov}_c\big(\log N_c(t_0),\, \log \bar{H}_c(t_0)\big)}{\text{Var}_c\big(\log N_c(t_0)\big)}.$$

Thus $\beta_{\text{between}}(t_0)$ is a property of the joint distribution of initial conditions and growth rates $(H_{0,c}, N_{0,c}, \mu_c, \eta_c)$ across cities at time t_0, and in general it is not equal to any particular city's ratio μ_c/η_c (nor to a simple average of these ratios). In other words, the longitudinal "trajectory" exponent β_c^{traj} and the cross-sectional scaling exponent β_{between} are fundamentally different objects: the former encodes the kinetics of individual city growth in calendar time, while the latter summarizes how cities of different size compare to each other at a given time slice. Figure 6 shows this β_{between} vs β_c^{traj} differences.

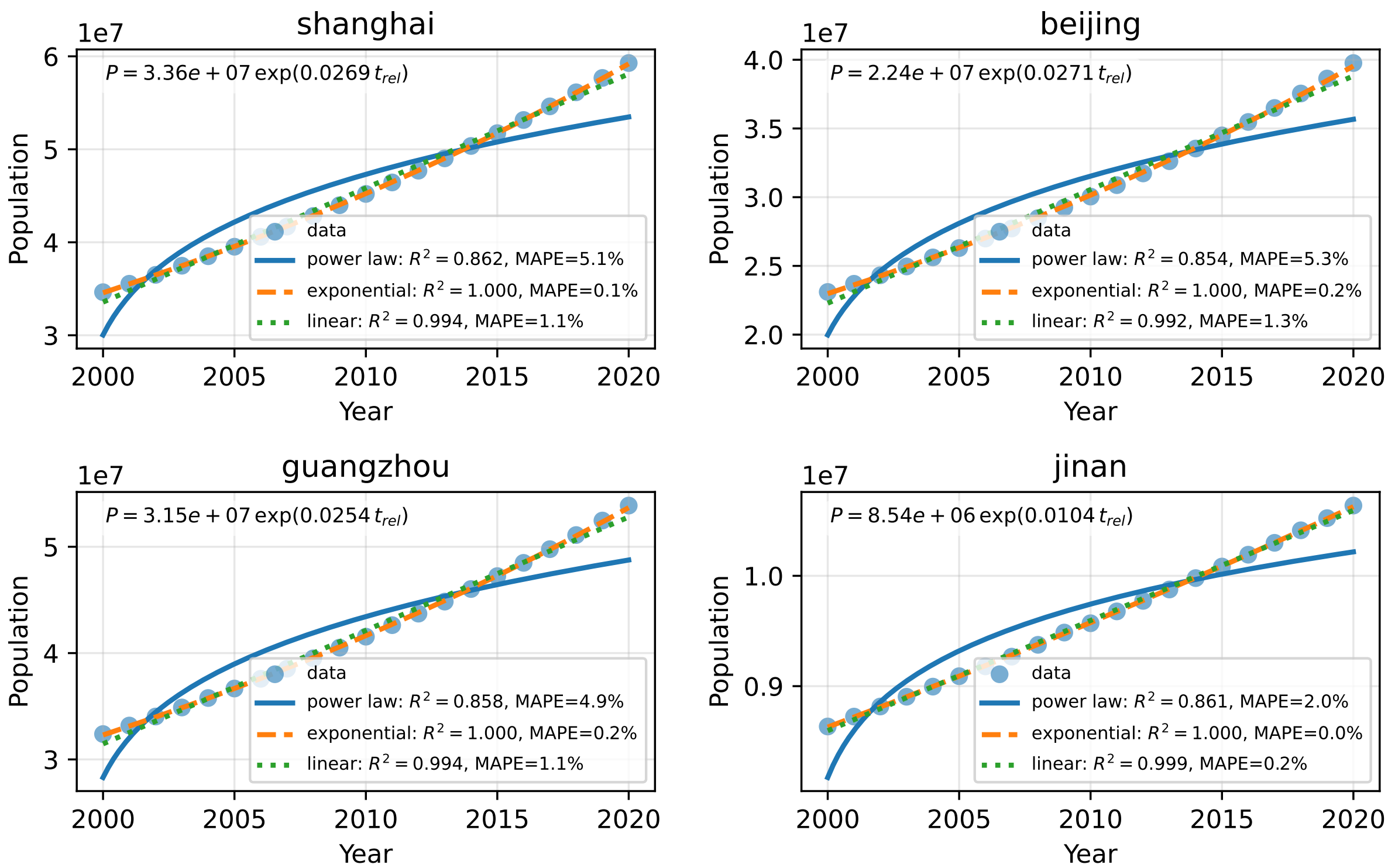

Figure 5: Temporal evolution of the population the largest Chinese cities.

4. **There is an asymmetry between changes in population and built form during periods of decline.** Population can adjust relatively quickly through migration and demographic change, so $N_c(t)$ may eventually stagnate or even decrease. By contrast, the vertical built form is highly inertial: once tall buildings are constructed, they typically remain in place for decades, even if they become partially vacant. In such regimes, the same value of $H_c(t)$ can correspond to very different values of $N_c(t)$, and the mapping $t \mapsto (N_c(t), H_c(t))$ is no longer approximately one-to-one in N. This hysteresis further illustrates that population is not a natural "clock" for height dynamics: N can move backward or fluctuate while the height field changes little, whereas calendar time continues to provide a simple, monotone parametrization of the evolution of the built environment. This mismatch is particularly clear in strongly supply-driven episodes of urbanization, such as the well-documented "ghost cities" in China (Shepard [2015]). In these cases, very large amounts of high-rise residential and commercial floor space are built in anticipation of future demand, often supported by financial and institutional incentives on the supply side. As a result, the height field $H_c(t)$ (and more generally the built volume) can increase rapidly while actual population $N_c(t)$ remains low for an extended period,

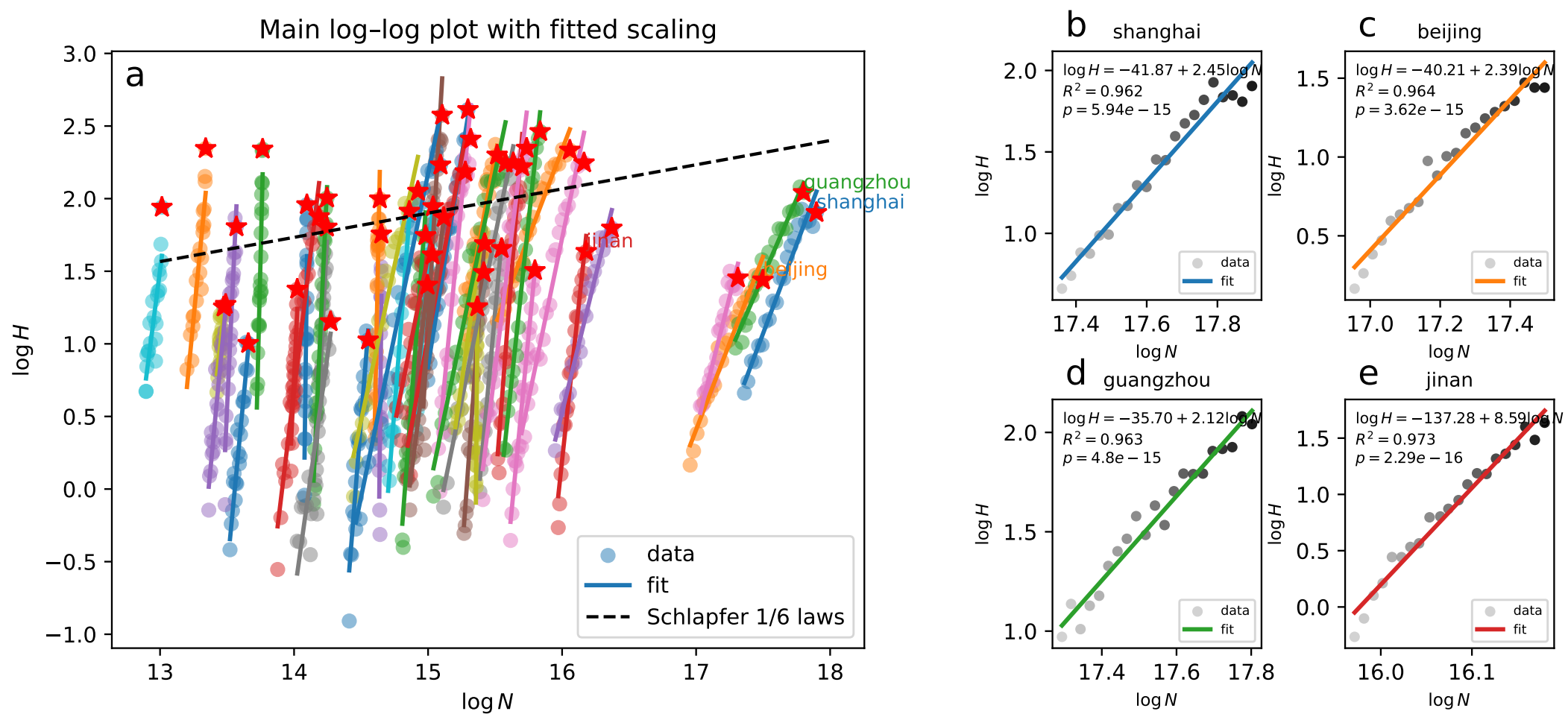

Figure 6: (a) Log–log relation between mean building height $\log H$ and population $\log N$ for Chinese cities, with per-city power-law fits $\log H(t) = a_c + \beta_c \log N(t)$ shown in colour (longitudinal power-law). Red stars mark the 2020 mean values, and the dashed line indicates the Schläpfer et al. [2015] scaling with exponent 1/6 (the cross-sectional). (b–e) Examples for four individual cities, showing their trajectories in $(\log N, \log H)$ over time (points shaded from light to dark with increasing year).

or even fails to materialize at all. Here, $\bar{H}_c$ is driven primarily by construction decisions and financing conditions rather than by contemporaneous N_c, and the same level of mean height may coexist with very different occupancy levels. Such "overbuilt" configurations vividly illustrate that the evolution of the vertical built form cannot be treated as a simple function of current population: the H–N relation is strongly path-dependent and dominated, in many regimes, by supply-side dynamics. This again supports the view that population is an appropriate size variable for cross-sectional comparisons, but not a natural clock for the longitudinal dynamics of building heights.

We have clarified all these points in the Discussion section **(Lines 239-265)** of the revised manuscript and added further details in the revised SI **(Lines 94-143)**.

C4:

Comment

The authors claim that their results align with the findings of ref.18 ([Kinetic roughening of the urban skyline...]). We cannot agree with this statement : ref.18 finds KPZ/EW scaling for h, while the manuscript shows KPZ scaling for $\phi = \log h$. Moreover, in the reviewer's view, the exponents measured in Fig.5a do not necessarily correspond to α (the roughness exponent of surface growth physics), but rather to an α_{loc}, describing the scaling of roughness at scales much smaller than the system size (see e.g [J.J Ramasco et al.,Generic Dynamic Scaling in Kinetic Roughening, PRL]). Could the authors clarify this point? Furthermore, for the sake of self-containment, an improved discussion on known universality classes in 2+1D kinetic roughening would help the reader (e.g known universality classes, exponents values).

Reply. We thank the referee for this important clarification. We fully agree that our previous

statement was misleading: Ref. Najem et al. [2020] reports kinetic-roughening behaviour for the height field h, whereas our analysis points to KPZ-like scaling for the transformed field $\phi = \log h$. In the revised manuscript we remove the claim of direct alignment and instead state more carefully that our modelling framework (multiplicative noise for h) naturally leads, via the Hopf–Cole transform, to an additive-noise KPZ-type description for $\phi = \log h$, which is a distinct statement from "h itself is KPZ".

Local vs. global roughness exponent. The referee is also correct that the exponents reported in our Fig. 5a are local roughness exponents, $\alpha_{\rm loc}$ in the sense of Ramasco et al. [2000], rather than global roughness exponents α obtained from finite-size scaling of the global width. Operationally, we compute the rms width of $\phi = \log h$ inside windows of linear size ℓ and fit

$$W(\ell) \sim \ell^{\alpha_{\rm loc}} \qquad (\ell \ll L),$$

over an intermediate range of scales. We do not extract a global scaling $W(L) \sim L^{\alpha}$ because city domains are finite, irregular, and heterogeneous (multiple centres, coastlines, discontinuities, non-universal masks), and the corresponding global-width fits are not robust across the sample.

Importantly, as emphasised by Ramasco et al. [2000], $\alpha_{\rm loc}$ need not coincide with the global α in the presence of anomalous roughening. Since our dataset does not allow a reliable estimation of the complementary exponents (growth exponent β and dynamic exponent z) needed to fully test Family–Vicsek versus anomalous scaling scenarios, we refrain from making a stronger universality-class identification based on $\alpha_{\rm loc}$ alone.

Universality classes in 2+1D kinetic roughening. Following the referee's suggestion, we have added a brief overview of the main universality classes commonly discussed in 2+1D (EW, KPZ, and quenched/disordered variants), including typical exponent values and a short discussion of local vs. global exponents and anomalous scaling. This provides context for our focus on $\phi = \log h$ and for the interpretation of the local roughness measurements.

We clarified all these points in the the revised manuscript (Discussion **Lines 330-351** and **Lines 390-406)**

C5:

Comment

Even though the proposed model allows both for inter- and intra- urban prediction, suggesting self-similarity of growth processes at different scales, it is unclear to the reviewer how this model (with 3 parameters) explains better the dynamics of intra-urban vertical growth than simpler models of 2+1d surface growth (e.g. solid-on-solid model, or other non-conservative equations). In particular, this model predicts (when $\gamma > 0$) unbounded growth for $\overline{h}$ when $t \to \infty$, which allows well to capture early time dynamics but is contradictory with the fact that the cost of buildings scale like degree-two polynomial function [see e.g. Ahfeldt and Barr and references therein] and hence that a cost-based reasoning would rather lead to conjecture an equilibrium between the benefits given by heights (density, economic productivity) and cost of building high – even at 'small' h.

Reply. We thank the referee for this important point. Our aim is not to propose a fully structural model of vertical development based on explicit cost–benefit optimisation, but rather a parsimonious stochastic description of observed intra-urban height dynamics over the finite time horizons covered by the data.

Our preference for the present model over standard 2+1d surface-growth equations is mainly empirical. A simple KPZ-type interpretation is difficult to reconcile with one of the most robust temporal signatures in the data, namely the evolution of the squared coefficient of variation. In $2+1$d KPZ with drift,

$$\mathbb{E}[h(t)] \approx v_\infty t, \qquad \mathrm{Var}(h(t)) \propto t^{2\beta}, \quad \beta \approx 0.24,$$

which implies

$$\frac{\mathrm{Var}(h(t))}{\mathbb{E}[h(t)]^2} \propto t^{2\beta-2} \approx t^{-1.52}.$$

Thus relative fluctuations should decay rapidly with time, whereas in our data $\mathrm{Var}(h(t))/\mathbb{E}[h(t)]^2$ tends instead toward an approximately constant value (Fig. 4c). In addition, multiplicative noise is natural for a positive accumulated quantity such as built height: it captures relative fluctuations in development intensity and preserves positivity without ad hoc constraints.

A second practical reason is the coarse resolution of the dataset. Each observation corresponds to a pixel of roughly $5\times5\,\mathrm{km}^2$, so we observe a coarse-grained height field rather than individual deposition events or interfaces. At this scale, a phenomenological stochastic equation is more directly connected to the observables that can be measured robustly in the data.

We agree that, taken literally as $t \to \infty$ with positive drift, the model implies unbounded growth, whereas urban-economic reasoning suggests finite optimal heights because construction costs rise more than proportionally with height Ahlfeldt and Barr [2022]. We therefore view the equation as an effective finite-horizon description, not as a literal asymptotic theory of city heights. This is similar to many growth models in statistical physics, whose long-run divergence is not interpreted literally when the relevant empirical regime is finite-time.

These two perspectives can in fact be reconciled. The equilibrium framework of Ahlfeldt and Barr [2022] describes how technology, land values, regulation, and demand determine finite location-specific heights. Our stochastic model can then be read as a reduced-form dynamics of the observed building stock around such evolving equilibria. In that interpretation, a purely time-dependent drift $\mu = \mu(t)$, with k and σ kept constant, already provides a simple way to represent changing macro growth conditions: if $\mu(t)$ declines over time, vertical amplification weakens, mimicking a slowdown in construction activity, and if $\mu(t) \to 0$ the model can capture near-stagnation in city-building growth. For longer-run applications, one can also introduce explicit saturation, for example through a logistic capacity term reflecting economic or regulatory constraints. By contrast, allowing all three fields (μ, σ, k) to vary freely in space and time would quickly create over-parameterisation and identifiability issues relative to the resolution of the available data.

In the revised manuscript we added clarifications in the Discussion **(Lines 216-238)** and in the Method more motivation about our modelling choice **(Lines 489-496)**.

C6:

Comment

The discussion on the (s)FKPP equation for 'mature cities' is unclear and would deserve a more nuanced and detailed discussion. FKPP fronts (eq.18 without the noise term) propagate at asymptotic speed $2\sqrt{D\mu}$ and, with a stable phase $h = K$ invading a $h = 0$ unstable phase. Hence this models cities heights dsitribution as essentially bimodal, with a very small front (as it typically decreases exponentially) separating the phases. This

model does not seem at first very convincing and a discussion explaining better 1) its derivation 2) what is the effect of noise on the reaction-diffusion fronts (shape and speed) 3) why it describes cities would help improving this part of the manuscript.

Reply. We thank the referee for this very pertinent comment. We agree that our previous discussion of the (s)FKPP equation in the "mature cities" paragraph was too brief and could give the misleading impression that we are literally proposing a travelling-front description of urban skylines. Our actual use of an FKPP-type term is more modest: we start from a stochastic heat equation with multiplicative noise (or GBM+interaction model) and then introduce a soft saturation scale K via a logistic drift by generalizing the drift parameter μh by $\mu h(1 - h/K)$. The intention is to showcase how our framework can be extended to impose limits on the maximum building height - in line with the previous comment/reply. We have now clarified this point in the revised manuscript (Method section **(Lines 499-500 and 504-517)**)

About the role of noise and absence of a single coherent front: in line with our general approach of viewing cities as emergent patterns arising from the amplification of fluctuations, we deliberately start from a spatially disordered height field: a random $h(x, 0)$ with small fluctuations everywhere, rather than from a pre-imposed sharp interface separating $h \approx 0$ and $h \approx K$. Therefore, the multiplicative noise term $\sigma\, h\, \eta(x, t)$ continuously injects fluctuations and roughens the field, so any interface between higher and lower regions is highly irregular, fragmented and space–time dependent rather than a smooth, coherent FKPP front. As a result, there is no single coherent FKPP front in our simulations; instead one observes a patchy landscape in which higher and lower regions interact and gradually saturate towards $h \leq K$, with local interfaces that fluctuate and wander (Figure 7).

The sFKPP form is therefore used as a phenomenological way to introduce an effective saturation scale K into the continuum description - and we can further assume that $K(x)$ should depend on city zoning (and also potentially vary in time). The logistic term can then be interpreted as an outer constraint that prevents unbounded growth and biases the dynamics towards $h \leq K$ in already urbanised regions.

A fully micro-founded description of city-wide expansion fronts, including an explicit determination of K and the shape and speed of invasion into genuinely undeveloped territory, would go beyond the scope of the present work. We now state these limitations **(Lines 362-382)** explicitly and present the sFKPP term as an exploratory extension that encodes saturation rather than as a literal front-forming model for the entire city.

C7:

Comment

what are the geometric consequences of this modelization ? How does the total volume scale, and how does the total ground surface scale ? What is the horizontal sprawl speed ?

Reply. We thank the referee for raising these natural questions about the geometric implications of our stochastic height model.

Total built volume. Let Ω denote the (fixed) ground domain over which we evolve the height

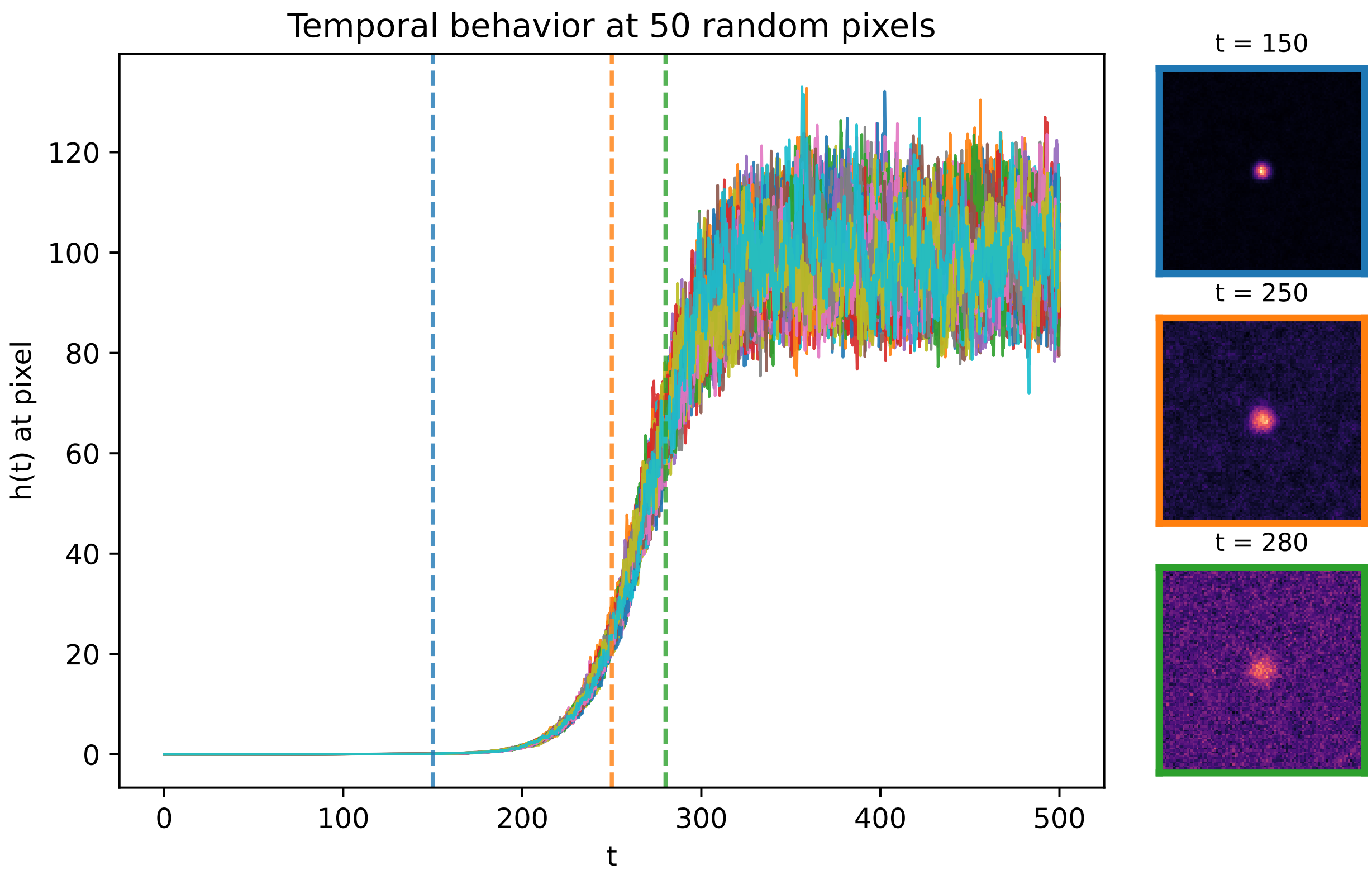

Figure 7: Stochastic Fisher–KPP (sFPP) simulation on a 500×500 periodic lattice, initialized with a random field and a localized high-density seed at the domain center and with a capacity $K = 100$. The main panel shows the temporal evolution $h_i(t)$ at n randomly sampled pixels, exhibiting a characteristic (noisy) sigmoidal growth (GBM like behavior for early time) towards saturation due to stochasticity and diffusion. Colored dashed vertical lines mark the snapshot times. Insets (with matching colored frames) display the spatial structure $h(x, y, t)$ at the corresponding times, illustrating the spreading and pattern formation of the evolving field that tends then to be homogenous with height around the capacity $K = 100$.

field, and define the total volume

$$V(t) \;=\; \int_{\Omega} h(x,t)\,\mathrm{d}x.$$

Therefore, the average is $\mathbb{E}[V(t)] \propto e^{\mu_{\mathrm{eff}} t}$, i.e. on a fixed ground area the total built volume grows approximately exponentially in calendar time, consistent with the empirical exponential trends in mean height that we document.

A natural way to quantify horizontal sprawl in our framework is to threshold the height field and work with the occupancy indicator

$$\chi(x,t) \;=\; \mathbf{1}_{\{h(x,t)>h_\star\}}.$$

Because $\chi(\cdot,t)$ is a nonlocal object (see for example Fig. 8), we characterise sprawl through the geometry of the largest connected component attached to a seed point (e.g., the historical core), denoted $\mathcal{C}_\star(t)$. As $h(x,t)$ grows and spatially redistributes, $\chi(x,t)$ expands and fragments, while $\mathcal{C}_\star(t)$ typically propagates outward in a rough, possibly anisotropic manner. The "urbanisation front" is typically defined as the external boundary of $\mathcal{C}_\star(t)$.

This construction is naturally connected to dynamic correlated percolation: since $h(x,t)$ is spatially correlated (via the interaction term and shared stochastic history), the thresholded field $\chi(x,t)$ is not an i.i.d. percolation pattern, but generates correlated clusters whose geometry evolves in time. Accordingly, we measure the built ground surface as

$$A_{\text{built}}(t) = \int_{\Omega} \chi(x,t)\,dx, \qquad A_{\text{LCC}}(t) = \int_{\Omega} \mathbf{1}_{\{x\in\mathcal{C}_\star(t)\}}\,dx,$$

and we report in Fig. 9 the time evolution of $\mathcal{C}_\star(t)$ together with $A_{\text{LCC}}(t)$ and the associated sprawl speed. In particular, $A_{\text{LCC}}(t)$ typically exhibits a sigmoid-like growth: the sharp increase corresponds to the percolation-like event where the seed-attached component spans the entire domain, after which the apparent front motion saturates due to finite-size effects.

Minor comments:

C8:

Comment

in the introduction, the causes of urban expansions should be discussed more accurately (with references)

Reply. We added a paragraph about that in the Introduction in the revised manuscript **(Lines 35-56)**.

C9:

Comment

discuss the values of RMSE (Fig.3) – how can I assess whether the fit provided by the model is good (Fig,3) ? Compare to a null/non-predictive model ?

Reply. We have added a discussion **(Lines 163-177)** about the performance of the model by comparing it to a null model (the Arithmetic Brownian Motion model which is a linear stochastic model) and revised Fig. 3 accordingly.

C10:

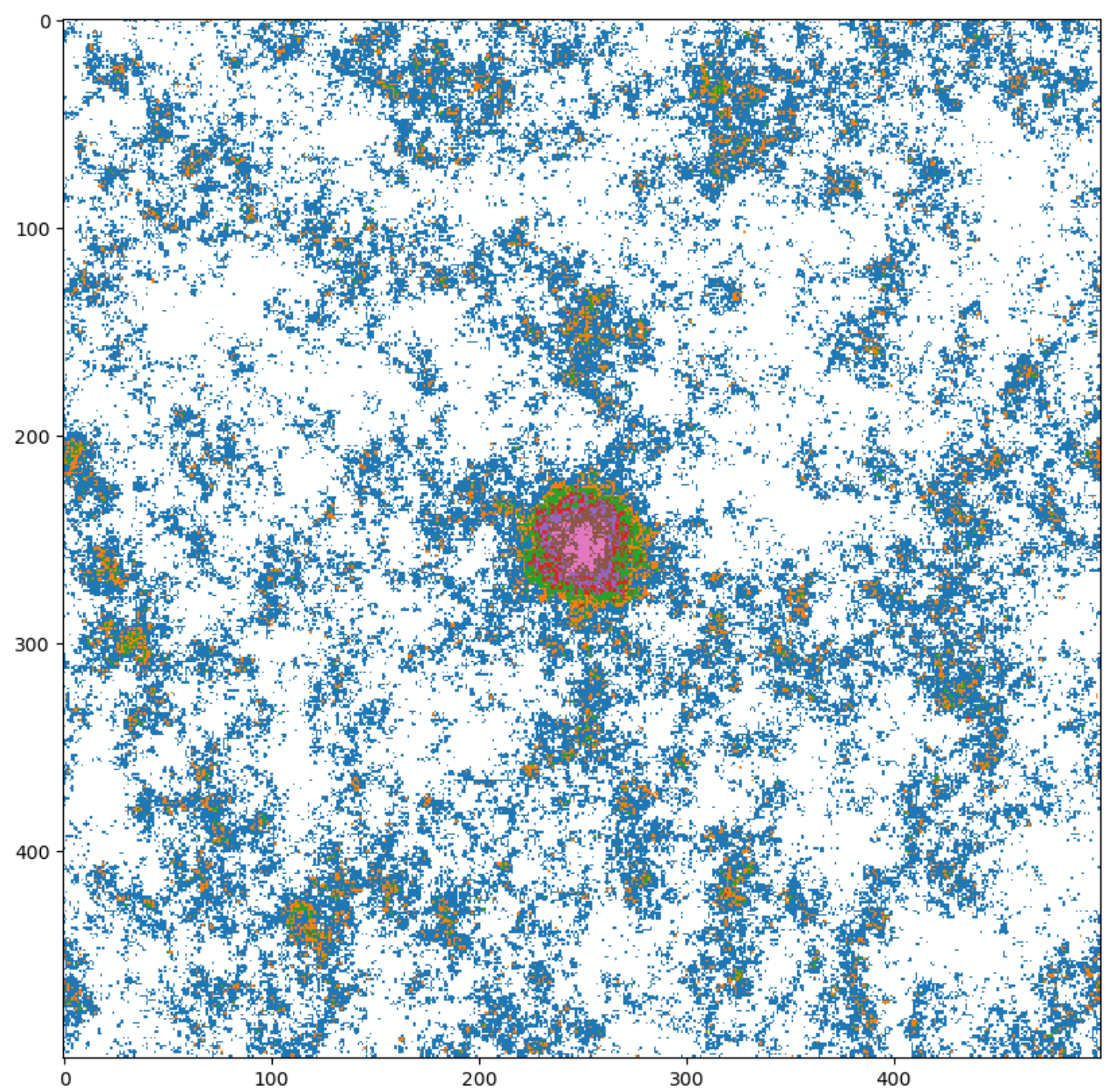

Figure 8: Illustration of the field $\chi(x,t)$ Eq. C7 for a given (fixed) $h_\star$. The colors corresponds to different time t. Pink at the centre correspond to early time, blue correspond to final time.

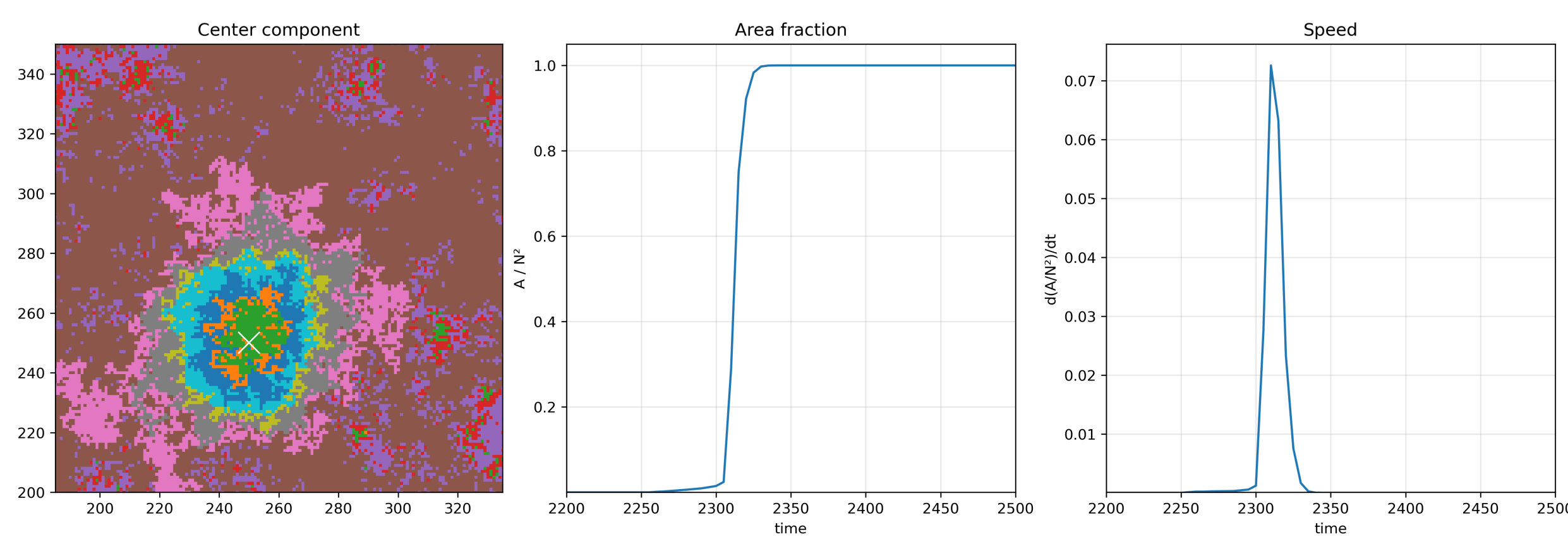

Figure 9: Geometric observables of horizontal sprawl extracted from the simulated height field via thresholding. (a) superlevel-set occupancy $\chi(x,t) = \mathbf{1}_{\{h(x,t)>h_\star\}}$ restricted to the largest connected component attached to a central seed, shown at several times (colors encode time). (b) built ground-surface fraction $A_{\mathrm{LCC}}(t)/N^2$, where $A_{\mathrm{LCC}}(t)$ is the area (number of lattice sites) of the seed-connected component above the threshold. (c) corresponding sprawl "speed" measured as the time derivative $\mathrm{d}(A_{\mathrm{LCC}}/N^2)/\mathrm{d}t$.

Comment

what about the effects of height restriction ? Typically discussed in urban economics, in

the context of efficiency of cities.

Reply. We thank the referee for this important point. In our GBM+interaction framework, the coupling parameter k could be interpreted as a regulation of dispersion (or inequality) in heights. k smooths gradients and reduces the persistence of highly localised peaks, thereby regulating the spatial heterogeneity of the skyline.

To model height restrictions in the urban-economics sense (i.e. saturation due to zoning limits, shadow constraints, aviation safety, or cost/engineering constraints), a natural extension is to introduce a saturating drift, e.g. $\mu h(1 - h/K)$, where K that leads to the stochastic Fisher–KPP (sFKPP) type equation that we already introduced in the manuscript, providing bounded long-run heights while preserving the early-time GBM-like regime when $h \ll K$. We now clarify this interpretation in the revised manuscript **(Lines 362-372)**.

Quantitatively calibrating such restrictions across cities and over time would require a dedicated global dataset on height regulations and their temporal evolution, which is (to our knowledge) not currently available at the scale of our study.

C11:

Comment

growth occurs on the horizontal-vertical axis according to a certain typology [Mahtta.R et al, Building up or spreading out? Typologies of urban growth across 478 cities of 1 million+, ERL] – where is this model situated on this typology?

Reply. We thank the referee for this helpful reference. The typology of Mahtta et al. classifies pixels by clustering a 4D descriptor

$$(\text{initial horizontal},\ \text{initial vertical},\ \Delta\text{horizontal},\ \Delta\text{vertical}),$$

and then interpreting clusters as "stabilized", "outward", "mature upward", "budding outward", or "upward & outward" growth modes.

Within this 4D space, our GBM+interactions model suggests a clear qualitative mapping controlled by the initial condition ("nascent" vs. already "mature" urban fabric), and the parameters (μ, k, σ):

- **Initial condition (nascent vs. mature) matters.** Because the typology explicitly conditions on initial horizontal/vertical state, the same dynamics can be classified differently depending on whether one starts from a small seeded built core (low initial horizontal/vertical) or from an already extensive/tall configuration (high initial horizontal/vertical), which is particularly relevant when observational windows begin after a city is already "large".
- **Increasing μ primarily moves pixels along the vertical axis.** The drift μ controls the mean multiplicative amplification of h and therefore increases Δvertical for pixels that are already developed; in the typology vocabulary this shifts mass toward "mature upward" (if horizontal change is weak) or "upward & outward" (if horizontal change is also strong).

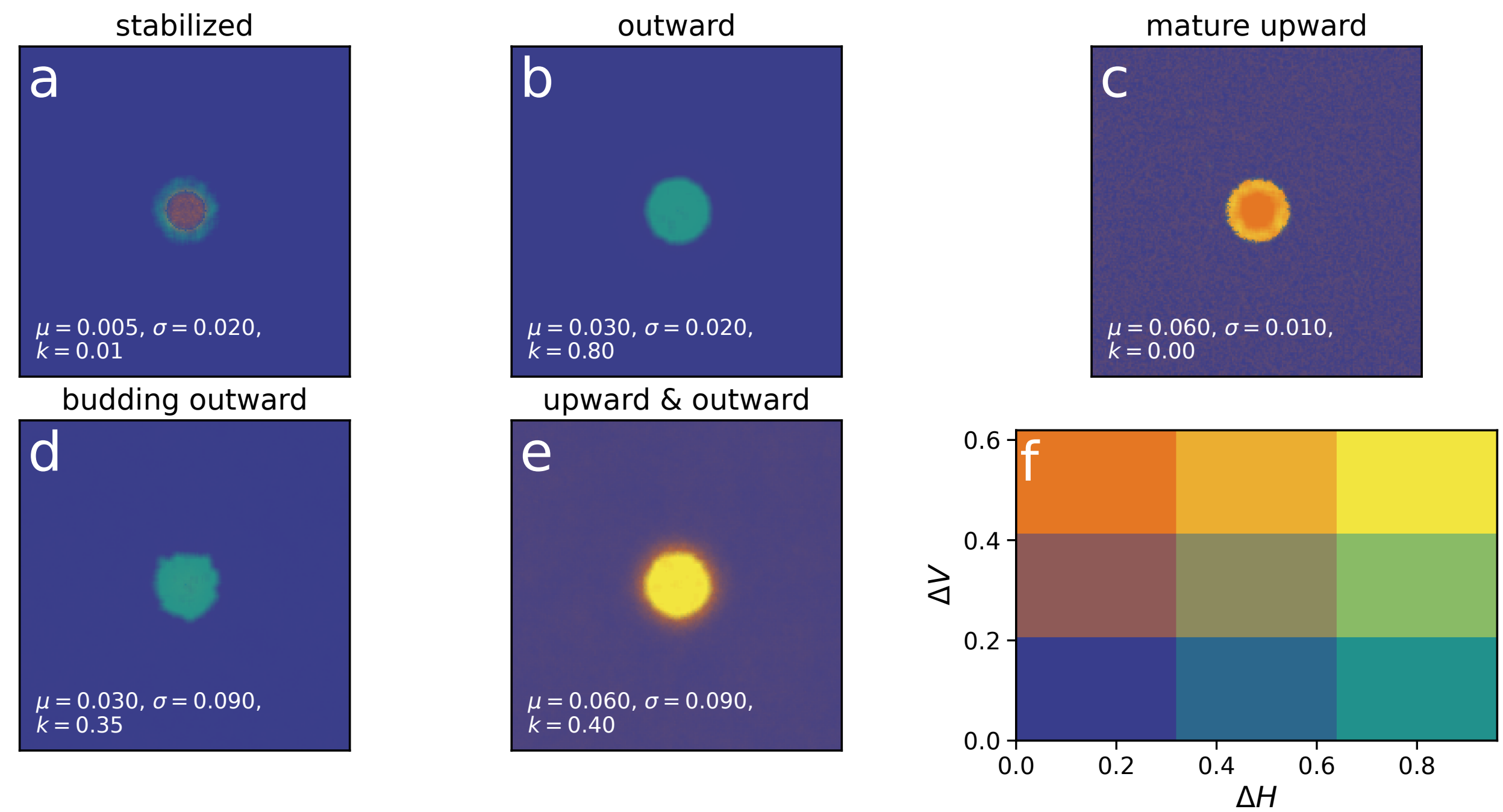

Figure 10: Bivariate typology maps from the GBM+coupling model. Panels (a–e) show the same simulation domain colored by a continuous bivariate colormap encoding (i) vertical change ΔV (local growth in $\log(1+h)$ from the initial to the final state) and (ii) horizontal change ΔH. The regimes are (a) stabilized, (b) outward, (c) mature upward, (d) budding outward, and (e) upward & outward. Panel (f) shows the bivariate color key mapping $(\Delta H, \Delta V)$ to color.

- **Increasing k primarily moves pixels along the horizontal axis.** The coupling term redistributes height to neighbours (smoothing gradients and propagating development) and thus increases Δhorizontal. In the typology vocabulary this promotes "outward"-type growth (or "upward & outward" when combined with large μ), with the important nuance that k tends to homogenise within connected regions.
- **Increasing σ increases intermittency and patchiness.** The multiplicative noise controls the magnitude of local fluctuations and thus the degree of heterogeneous, bursty development. In the typology language, this tends to produce more irregular, "budding"-like outward patterns (fragmented clusters and localised nucleation), especially when the initial condition is nascent.

Figure 10 illustrates how model parameters affects the vertical and horizontal changes. We added this new results and methodology explanation in the revised SI of the paper **(Lines 37-85)** and refer to them in the Discussion **(Lines 319-329)**.

C12:

Comment

the authors mention 'KPZ' : the KPZ equation maps to various universality classes when the noise nature changes (annealed, quenched, columnar-quenched, spatio-temporaly correlated...). – it would be worth mentioning it and explaining the scaling differences between the various universality classes.

Reply. We agree; in the revised manuscript we add a dedicated paragraph in the Discussion **(Lines 390-406)**, summarizing how the universality class and scaling exponents change when the KPZ/EW noise is annealed, quenched (qKPZ, qEW) or spatio-temporally correlated.

C13:

> **Comment**
>
> What is the typical time scale of relaxation to the profile to p_∞ ?

Reply. In the mean-field approximation it is natural to study relaxation in terms of the normalized variable $u(t) := h(t)/\overline{h}(t)$, whose stationary distribution is $p_\infty(u)$. Although the full time-dependent Fokker–Planck equation is not solvable in closed form, the approach to stationarity can be read directly from the moment dynamics. In particular, for $k > 0$ and $r = \sigma^2/(2k) < 1$, the relative variance

$$w(t) := \frac{\mathrm{Var}[h(t)]}{\overline{h}(t)^2}$$

relaxes exponentially fast to its asymptotic value $w_\infty = r/(1-r)$ as

$$w(t) = w_\infty \Big(1 - e^{-2k(1-r)t}\Big).$$

This identifies a characteristic relaxation timescale

$$\tau_{\mathrm{rel}} \sim \frac{1}{2k(1-r)} = \frac{1}{2k-\sigma^2}, \qquad (r < 1),$$

i.e. stronger coupling k accelerates relaxation, whereas stronger multiplicative noise σ slows it down and the timescale diverges as $r \to 1^-$. We added a sentence explaining this in the Methods section of the revised manuscript **(Lines 459-466)**.

References

Gabriel M Ahlfeldt and Jason Barr. The economics of skyscrapers: A synthesis. *Journal of Urban Economics*, 129:103419, 2022.

Markus Schläpfer, Joey Lee, and Luís Bettencourt. Urban skylines: building heights and shapes as measures of city size. *arXiv preprint arXiv:1512.00946*, 2015.

Michael Batty, Rui Carvalho, Andy Hudson-Smith, Richard Milton, Duncan Smith, and Philip Steadman. Scaling and allometry in the building geometries of greater london. *The European Physical Journal B*, 63(3):303–314, 2008.

Joseph E Cavanaugh and Andrew A Neath. The akaike information criterion: Background, derivation, properties, application, interpretation, and refinements. *Wiley Interdisciplinary Reviews: Computational Statistics*, 11(3):e1460, 2019.

Wade Shepard. *Ghost cities of China: The story of cities without people in the world's most populated country.* Bloomsbury Publishing, 2015.

Sara Najem, Alaa Krayem, Tapio Ala-Nissila, and Martin Grant. Kinetic roughening of the urban skyline. *Physical Review E*, 101(5):050301, 2020.

José J Ramasco, Juan M López, and Miguel A Rodríguez. Generic dynamic scaling in kinetic roughening. *Physical review letters*, 84(10):2199, 2000.

Response to reviewers' comments

Martin Hendrick and Gabriele Manoli

March 1, 2026

Reviewer #2

General remarks: In this manuscript the authors follow cities' skyline as a moving front, which is followed through the evolution of the buildings' heights dynamics. They model this process as a zero-dimensional geometric Brownian motion with multiplicaltive noise, which is augmented with spatial coupling. They then show that this interplay of the two aforementioned effects drives the morphological changes and that the Kardar-Parisi- Zhang (KPZ) equation is recovered in the continuum limit.

The growth of the skyline has been previously shown to follow either the KPZ or the Edwards-Wilkinson (EW) universality classes [1], and in that respect the work is trying to build a ground-up model linking model parameters to economic productivity, which is an intersting take on explainability of the dynamics. The work is an attempt to explain an already observed feature of the skyline using a bottom-up model. The findings, though interesting, represent an extension rather than a breakthrough.

Reply. We thank the referee for this careful summary and for raising the question of novelty with respect to earlier work on kinetic roughening of skylines, in particular Najem et al. [2020]. We agree that our starting point is close in spirit to Najem's work, namely to interpret the skyline as a fluctuating interface and ask whether its roughening is compatible with EW/KPZ universality. At the same time, we believe that our contribution goes beyond a straightforward extension in several substantive ways.
(i) KPZ for $\log h$ *rather than for* h *itself.* Najem et al. focus on the roughness of the height field h and classify cities by comparing the measured roughness exponents to those of the EW and KPZ equations. In contrast, our empirical analysis shows that the natural coarse-grained field satisfying the KPZ equation is the log-height $\phi = \log h$, not h itself. At the level of single-pixel trajectories we find that vertical growth is well described by Geometric Brownian Motion (GBM), with multiplicative noise and a city-specific drift. When we add spatial coupling, the continuum limit of our model is the stochastic heat equation for h with multiplicative noise, which via the Hopf–Cole transform corresponds to a KPZ-type equation for $\phi = \log h$. This is conceptually different from assuming a priori the KPZ equation for h itself, and is supported by several empirical signatures (e.g., the behaviour of $\mathrm{Var}(h)/\mathbb{E}[h]^2$ in time) that are not consistent with a KPZ-on-h interpretation but are consistent with multiplicative noise and an additive-noise KPZ description for $\log h$.
(ii) From a national, essentially static analysis to a global, explicitly dynamical one. Najem et al. work with an impressive dataset for the Netherlands and focus on roughness exponents to classify cities at a given time. In our case we exploit a global, multi-decadal dataset of building heights and follow the evolution of $h(x,t)$ over ~ 30 years in hundreds of cities worldwide. This allows to estimate dynamic parameters (drift, volatility, correlation length) of a stochastic evolution

equation city by city, and to relate them to macroeconomic indicators. In other words, we move from a primarily cross-sectional classification of universality classes in one country to a genuinely spatiotemporal, global analysis with an explicit dynamical model.

(iii) An explicit coarse-grained stochastic model tied to economic observables. Whereas Najem et al. [2020] measure roughness exponents and then point to existing discrete models (such as RSOS) as possible microscopic analogues, they do not propose or calibrate a specific stochastic evolution equation for the skyline itself. By contrast, we start from a zero-dimensional GBM calibrated on building-height time series, augment it with spatial coupling, and show that this GBM+interaction model reproduces not only roughness exponents (for ϕ) but also temporal statistics and cross-city variability. The resulting parameters (drift μ, volatility σ, coupling k) can be related to economic productivity (e.g. GDP growth), providing a transparent link between the physics-style interface description and standard urban-economic quantities.

(iv) New observables beyond standard allometric laws. Most of the urban-scaling literature works with cross-sectional allometries of the form $Y \sim N^{\beta}$ (where N is the total population and Y is a generic property) between city-level aggregates. In addition to roughness exponents, we introduce and analyse dynamical observables that are, to our knowledge, not commonly used in urban science as distributions of log-returns of height, temporal behaviour of $\mathrm{Var}(h)/\mathbb{E}[h]^2$. These quantities are directly tied to the GBM/KPZ framework and help identify the appropriate coarse-grained dynamics rather than just a static scaling exponent.

(v) A predictive framework for the full height distribution (and beyond). Finally, our GBM+spatial-coupling framework is not only descriptive but also predictive: once parameters are estimated, it yields explicit predictions for the distribution of building heights across cities and its evolution in time, rather than only for a single roughness exponent. We also show that the same GBM-based approach performs well for other urban quantities (e.g., city GDP), suggesting that multiplicative-noise dynamics moderated by local interactions may be a more general mechanism in urban growth.

In this sense, we fully agree that our work builds on the insight of Najem et al. that urban skylines can be understood within standard kinetic roughening. However, we believe that (a) identifying $\log h$ as the KPZ field, (b) extending the analysis to a global, explicitly time-resolved dataset, and (c) constructing and calibrating an explicit stochastic model linked to economic observables and capable of predicting full height distributions represents a substantial step beyond the existing literature, rather than a purely incremental extension. Explicitly connecting urban growth with the theory of surface growth and universality classes has been highlighted as a promising venue for research by a recent review on the topic (Barthelemy and Marquis [2026]) - and this is precisely the breakthrough presented here.

The following questions arise:

C1:

> **Comment**
>
> The KPZ is a universality class and thus it subsumes numerous alternative growth models. Subsequently, the current proposed model is one of many that could explain the dynamics and it would be valuable to compare the it with equally valid models such as restricted solid on solid (RSOS), Eden model, asymmetric simple exclusion (ASEP) [2]. A likelihood measure should be provided in support of this model as opposed to others.

Reply. We thank the referee for this thoughtful comment. We fully agree that KPZ is a universality class and that many distinct microscopic models (RSOS, Eden, ASEP, etc.) can lead to the same large-scale exponents. Our goal in this paper is therefore not to identify

a unique microscopic rule, but rather to determine which field and idealy which universality class best describe urban height dynamics, and to propose the simplest analytically tractable coarse-grained model consistent with the observables that our data actually resolve.

A key practical constraint is the resolution of the dataset. Each pixel in our global building-height dataset aggregates the vertical structure of an area of order $5\times5\,\mathrm{km}^2$. At this scale we do not observe individual interfaces or deposition events, but a coarse-grained height field $h(x,t)$ that already integrates over many microscopic degrees of freedom. Directly fitting RSOS, Eden or ASEP to such data would require specifying an ad hoc mapping from site-level variables to pixel-averaged heights and embedding large-scale simulations into a likelihood for each city. This is not only computationally prohibitive, but also conceptually underdetermined: many different microscopic rules could reproduce the same coarse-grained field.

Because of this scale mismatch, we instead focus on robust observables we can measure from the data (log-returns of height, temporal growth of moments, spatial roughness, etc.) and ask: *what is the simplest continuum stochastic equation that reproduces these observations and can be analysed analytically?* This leads us to a GBM with diffusive coupling for h. Then, via the Hopf–Cole transform, this is equivalent to the KPZ equation for the field $\phi = \log h$. Thus, KPZ-like behaviour holds for log-height $\log h$, not for h itself; this is, in our view, a key novelty of the present work.

We have also checked that a naive "KPZ-on-h" interpretation is inconsistent with one of the simplest temporal signatures that we can measure: the evolution of the squared coefficient of variation. In $2+1$D KPZ with a drift,

$$\mathbb{E}[h(t)] \approx v_\infty t, \qquad \mathrm{Var}(h(t)) \propto t^{2\beta}, \quad \beta \approx 0.24(\text{in 2+1 d}),$$

so that

$$\frac{\mathrm{Var}(h(t))}{\mathbb{E}[h(t)]^2} \approx \frac{C\,t^{2\beta}}{(v_\infty t)^2} = \text{const} \times t^{2\beta-2}.$$

Thus one has:

$$\frac{\mathrm{Var}(h(t))}{\mathbb{E}[h(t)]^2} \propto t^{-1.52},$$

i.e. the squared relative fluctuations of h should decay rapidly with time. In our empirical data, by contrast, we find that $\mathrm{Var}(h(t))/\mathbb{E}[h(t)]^2$ tends to a constant as t increases (as shown in Figure 4c in the manuscript). This behaviour is naturally explained by a multiplicative (GBM-like) dynamics for h.

Concerning the request for a likelihood measure: at the coarse-grained level accessible to our data, we performed, for the revised manuscript (in the revised SI **(Lines 95-133)**), likelihood-based comparisons between several stochastic models (GBM, Arithmetic Brownian Motion (ABM), GBM with additive noise) and report log-likelihoods and AIC (Akaike Information Criterion; Cavanaugh and Neath [2019]) values. These are processes for which the transition densities of the observed increments are available in closed form, so that writing and maximising the likelihood is well-posed. For microscopic RSOS/Eden/ASEP dynamics, by contrast, there is no simple analytic likelihood for the pixel-aggregated heights, and any comparison would require nested Monte Carlo simulations at the lattice scale for each parameter set and each city, which is well beyond the scope of this paper.

Finally, modelling h with multiplicative noise is conceptually natural for a positive accumulated field like building height. Random shocks are more plausibly relative (percentage

fluctuations in development intensity, investment, demand, etc.) than additive meter-scale increments, implying scale-dependent fluctuation (larger built stocks exhibit larger absolute fluctuations) and preserving positivity of h without ad hoc boundary conditions. The connection with additive-noise KPZ/EW-type fluctuations emerge more naturally for the transformed field $\phi = \log h$.

We now stress this point more clearly in the revised manuscript, especially in the Discussion **(Lines 216-238)** and in the Methods **(Lines 489-496)**.

C2:

> **Comment**
>
> There are multiple parameters in the model, each of which could have its own economic/regulatory correspondances. In particular k could reflect regulations imposed on building heights. For example, when the authors follow the mean-field formulation, the coupling k effectively multiplies hbar, which is a regulatory value on mean building height in the city. No comments on this were made.

Reply. We thank the referee for this insightful comment. We agree that the parameters of our coarse-grained model should admit natural economic and regulatory interpretations, and that this deserves to be discussed more explicitly in the text. We consider these parameters as effective quantities: they summarise, at the pixel scale of $5 \times 5\,\mathrm{km}^2$, the combined effect of many underlying mechanisms (markets, technology, regulations, geography), and we are cautious about attributing them one-to-one to a single cause. That said, we agree that a qualitative mapping is useful and we have added a short discussion and a summary table in the revised manuscript.

Concerning the spatial coupling k in particular: Intuitively, this coupling tries to reduce local height differences and drive h_i towards a locally averaged height $\bar{h}$; in mean-field, deviations from the city-wide mean are damped at a rate proportional to k. Economically, this "relaxation to the local mean" can be seen as the effective strength of forces that homogenise height: regulatory constraints (zoning, maximum height, floor–area ratio rules), but also market mechanisms (land and construction costs, competition for views, agglomeration externalities) and technical constraints (shared infrastructure, engineering limits). In more tightly regulated or more spatially integrated contexts one would expect a larger effective k; in looser or more heterogeneous environments, a smaller k.

We would like to stress, however, that in our formulation $\bar{h}$ is an endogenous average (local or city-level), not an exogenously imposed regulatory ceiling. We do not impose a height limit into the dynamics; instead, $\bar{h}$ emerges from the collective outcome of many buildings, and the coupling k measures how fast individual pixels "feel" and adjust to this emergent profile.

However, when we introduce a saturation scale K like in the sFKPP-like extension **(Lines 497-517)**, this plays a role closer to a soft capacity constraint, but even there it is modelled as a smooth logistic drift rather than as a hard maximum. If one wanted to model explicit regulatory caps, one would indeed need to add non-linear terms that enforce a maximum height more sharply.

We agree that a systematic cross-city study of how k correlates with independent measures of regulatory stringency (zoning indices, floor–area ratio limits, etc.) would be highly valuable, but this would require assembling a separate, global database of land-use and building-code indicators, which is beyond the scope of the present paper.

Regarding the other parameters: In an urban interpretation, μ acts as a local mean growth rate (economic attractiveness, demand, profitability, land rents), σ controls local volatility (speculative dynamics, heterogeneous development intensity, boom–bust fluctuations), while k sets the strength of spatial coupling (how quickly local shocks diffuse to neighbours; shaped by transport connectivity, technical constraints, and potentially regulation). We added in the revised version of the manuscript a discussion **(Lines 305-319)** about how to interpret model's parameters.

C3:

> **Comment**
>
> It would be valuable if the authors could look at cities where k changes both temporally and spatially and follow the evolution of the GBM. Can the effect of a changing k be modeled? This would be a more interesting dynamical evolution of the growth.

Reply. We agree with the referee that allowing the coupling parameter k to vary in space and time is conceptually very interesting. In our interpretation, k is an effective homogenisation strength, summarising the combined effect of zoning, land-use mix, infrastructure, and market integration. In principle one could introduce a space–time dependent coupling $k(x,t)$, with the continuum limit

$$\partial_t h(x,t) = \mu\, h(x,t) + \nabla \cdot \big(k(x,t)\, \nabla h(x,t)\big) + \sigma\, h(x,t)\, \xi(x,t),$$

and study how locally stronger or weaker coupling modifies the morphology of the skyline.

In practice, however, our ability to estimate $k(x,t)$ from the current data is very limited. First, we only observe building heights at a relatively coarse spatial resolution, and we do not have a consistent global dataset of intra-urban zoning or building regulations over a 30-year period. Inferring a full $k(x,t)$ field would require, at minimum, high-resolution time-resolved information on land-use types (residential / commercial / industrial / mixed), changes in height limits, floor-area ratio rules, etc., for each city. Such data are not currently available at the global scale, and even for single metropolitan areas they are hard to assemble in a homogeneous way. For this reason, in the present paper we restrict attention to a city-level effective k that captures the overall strength of spatial interaction in a given urban system.

That said, from a modelling standpoint the extension to heterogeneous coupling is straightforward, and we now include in the Supplementary Information **(Lines 86-94)** an illustrative simulation in which k varies in space (Fig. 1).

More generally, one could also accommodate heterogeneous local growth and local volatility by allowing $\mu = \mu(x,t)$ and $\sigma = \sigma(x,t)$. In particular, even a purely time-dependent drift $\mu = \mu(t)$, with k and σ kept constant, provides a simple way to represent changing macro growth conditions: if $\mu(t)$ decreases over time, the model exhibits progressively weaker vertical amplification, mimicking a slowdown in construction activity, and if $\mu(t)$ approaches zero it can capture near-stagnation in city-building growth. However, allowing all three fields (μ, σ, k) to vary freely in space and time would introduce substantial over-parameterisation and identifiability issues.

C4:

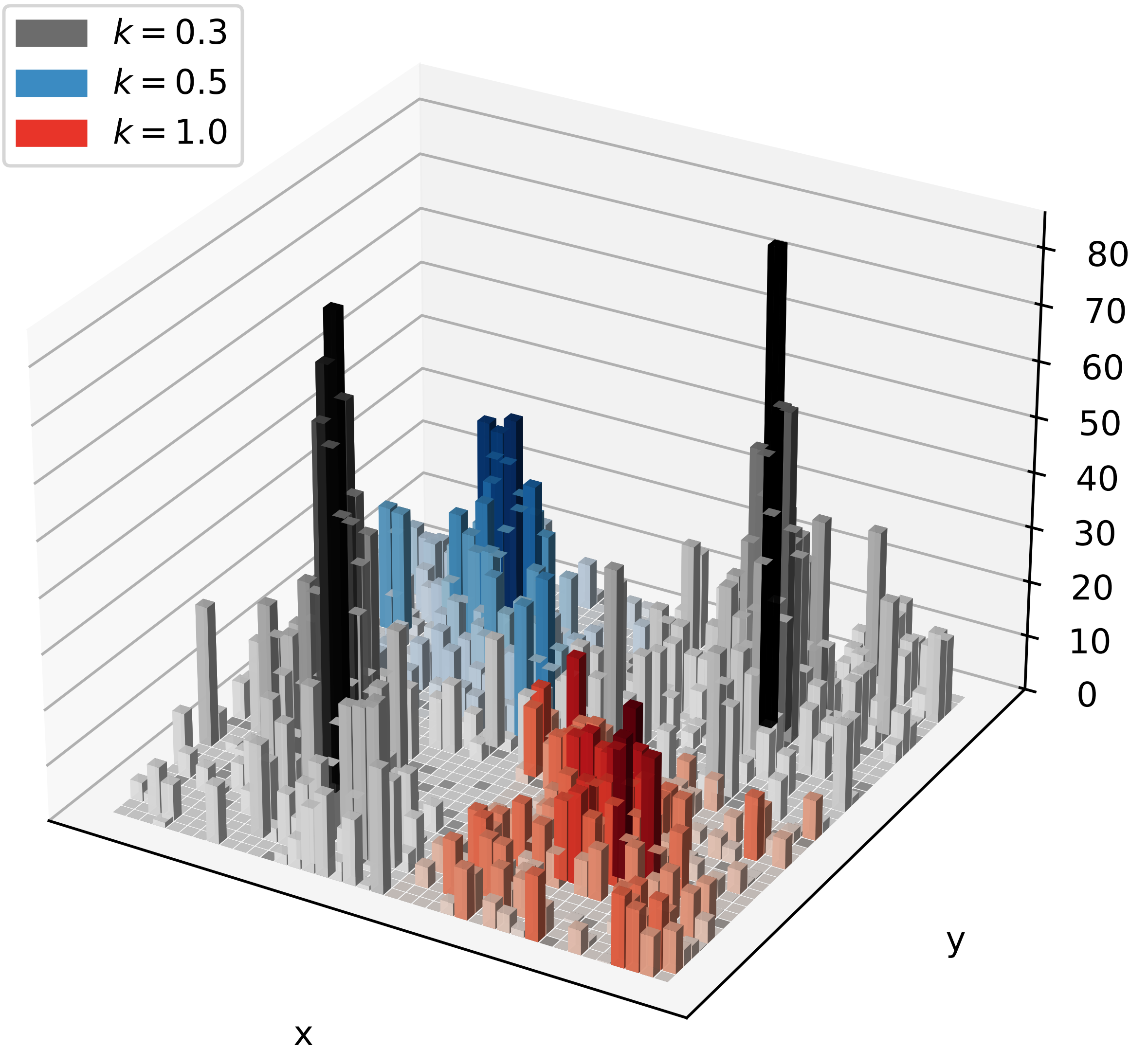

Figure 1: Three-dimensional bar rendering of the simulated height field $h(x, T)$ on a coarse lattice, where bars represent local "building" heights and colors encode a simple zoning proxy through a piecewise-constant coupling field $k(x)$. The domain is partitioned into districts with $k \in \{0.3, 0.5, 1\}$ (grey/blue/red, respectively), and within each district the color intensity is scaled by the local height to preserve intra-zone contrasts. The plot illustrates how heterogeneous spatial coupling (interpretable as differential intra-urban connectivity or homogenisation across zones) produces visually distinct spatial structures under identical GBM parameters (μ, σ).

Comment

It would be also valualble to link varrying k to zoning within cities? intracity clustering into residential, industrial, mixed...

Reply. Please refer to the previous reply.

C5:

Comment

The whole work is technically sound however the contribution does not seem to introduce fundamentally new concepts. I recommend revisiting the work and addressing the comments and questions provided to substantiate the work with more evidence and relevance to the urban complexity community.

Reply. We thank the referee for the positive assessment of the technical soundness and for the encouragement to better position our contribution. We have revised the manuscript in response to the detailed comments above, and we hope that the relevance of our findings for the urban complexity community is now clearer.

In particular, in the revised manuscript we have:

- highlighted the novelty of our work, clarified the modelling rationale for multiplicative noise, and emphasized the inference constraints imposed by the coarse spatial resolution of the global height dataset;
- expanded the discussion on parameter interpretation, stressing their effective (coarse-grained) meaning and providing qualitative economic/regulatory correspondences;
- strengthened the connection to standard urban-science observables by adding additional morphological validations (e.g., height profiles, spatial correlations, and links to growth typologies);
- illustrated natural extensions of the framework to heterogeneous environments by allowing parameters to vary in space (and, in principle, time), with a toy example motivated by zoning-like heterogeneity.

Rather than proposing an entirely new universality class, our aim is to provide a clear, data-driven and analytically tractable link between established interface-growth universality (in the appropriate field) and urban form and dynamics at the global scale. We hope that the clarifications and additional results now included in the manuscript address the referee's concern about evidence and relevance.

References

Sara Najem, Alaa Krayem, Tapio Ala-Nissila, and Martin Grant. Kinetic roughening of the urban skyline. *Physical Review E*, 101(5):050301, 2020.

Marc Barthelemy and Ulysse Marquis. Mathematical modelling of urban sprawl. *Spatial Economic Analysis*, pages 1–21, 2026.

Joseph E Cavanaugh and Andrew A Neath. The akaike information criterion: Background, derivation, properties, application, interpretation, and refinements. *Wiley Interdisciplinary Reviews: Computational Statistics*, 11(3):e1460, 2019.
